# Orb-weaving spider *Araneus ventricosus* genome elucidates the spidroin gene catalogue

**DOI:** 10.1038/s41598-019-44775-2

**Published:** 2019-06-10

**Authors:** Nobuaki Kono, Hiroyuki Nakamura, Rintaro Ohtoshi, Daniel A. Pedrazzoli Moran, Asaka Shinohara, Yuki Yoshida, Masayuki Fujiwara, Masaru Mori, Masaru Tomita, Kazuharu Arakawa

**Affiliations:** 1 Institute for Advanced Biosciences, Keio University, 246-2 Mizukami, Kakuganji, Tsuruoka, Yamagata 997-0052 Japan; 2 Spiber Inc., 234-1 Mizukami, Kakuganji, Tsuruoka, Yamagata 997-0052 Japan; 3 Graduate School of Media and Governance, Keio University, 5322 Endo, Fujisawa, Kanagawa 252-0882 Japan

**Keywords:** Genome, Proteomics

## Abstract

Members of the family Araneidae are common orb-weaving spiders, and they produce several types of silks throughout their behaviors and lives, from reproduction to foraging. Egg sac, prey capture thread, or dragline silk possesses characteristic mechanical properties, and its variability makes it a highly attractive material for ecological, evolutional, and industrial fields. However, the complete set of constituents of silks produced by a single species is still unclear, and novel spidroin genes as well as other proteins are still being found. Here, we present the first genome in genus *Araneus* together with the full set of spidroin genes with unamplified long reads and confirmed with transcriptome of the silk glands and proteome analysis of the dragline silk. The catalogue includes the first full length sequence of a paralog of major ampullate spidroin *MaSp3*, and several spider silk-constituting elements designated SpiCE. Family-wide phylogenomic analysis of Araneidae suggests the relatively recent acquisition of these genes, and multiple-omics analyses demonstrate that these proteins are critical components in the abdominal spidroin gland and dragline silk, contributing to the outstanding mechanical properties of silk in this group of species.

## Introduction

Large nocturnal spider, *Araneus ventricosus* (family: Araneidae, superfamily: Araneoidea), is a common orb-weaving spider found throughout Japan and East Asia that builds vertical webs (perpendicular to the ground). Silks of *A. ventricosus* have served high extensibility, toughness, and strength^1,2^; there is considerable interest in industrial applications of synthetic spider silks.

Araneoids have seven specialised types of abdominal silk glands and use them differently in various situations throughout their lives^3–9^. Interestingly, many of the silk proteins produced in each gland are encoded by different orthologue groups of the spidroin gene family^10,11^ that likely diverged before the divergence of spider families. Furthermore, paralogues within these orthologue groups have been reported. For instance, two types of tubuliform genes (CySp or TuSp) used as the outer shell of the egg case were found in *Argiope bruennichi*^12^. Moreover, eight types of dragline silk genes, major ampullate spidroin (MaSp), were reported in *Nephila clavipes*^13^. Proteomic studies of spider silks are also identifying protein constituents other than spidroins, and the full catalogue of silk-related genes is yet to be uncovered.

The main difficulty in the study of spidroins is due to the unique organisation of these genes. Spidroin genes are very long, typically on the order of 10 k bp, and are almost entirely comprised of repetitive sequences between conserved non-repetitive N/C-terminal domains^11,14–18^. Such highly repetitive sequence structure poses a great challenge in sequence assembly based on short reads (including Sanger sequencing), and PCR amplification often results in chimeric artefacts. A common approach of target capture-based sequencing avoids misamplification, but is not optimal in finding novel spidroins. Previous approaches in genomic sequencing used PCR amplification, which can obtain a comprehensive list of spidroins, but the sequences tend to be partial, incomplete, or chimeric. Babb and colleagues exemplified the importance of genomic data to fully understand the diversity of spidroin genes^13^, using the draft genome data of *N. clavipes* and long read sequencing of spidroins but with long-range PCR amplicons. Many of their sequences still contain gaps and are thus not complete, and even if a spidroin-like gene sequence is obtained, the expression and presence of the protein products in actual silks should be further confirmed. Hence, the finding or isolation of the new spidroin gene requires multi-omics confirmation based on a high quality genome assembled with unamplified single molecule long read sequencing.

To this end, here, we present the draft genome of *A. ventricosus*, including full spidroin gene sets with a hybrid sequencing approach. These data sets will be a powerful reference to study the full extent of spidroin diversity and evolution. Using the draft genome, and transcriptomic as well as proteomic analyses, we reveal the unexpected complexity of *A. ventricosus* spider silk genetics.

## Results

### Genome sequence of *A. ventricosus*

We report the genome of *A*. *ventricosus* sequenced using a hybrid sequencing with a combination of Nanopore, 10x GemCode and Illumina technologies. Nanopore sequencing produced approximately 5.5 million long reads with a N50 length of 7.4 kbp (Table S1), and the latter produced over 500 million GemCode barcoded 150-bp paired-end reads. These sequenced reads were assembled into 300,730 scaffolds (Longest 9.34 Mbp, N50 scaffold size: 59,619 bp) comprising a 3.66 Gb genome (Table 1). The genome size estimated from the kmer distribution was 2.16 Gbp, with 37.4% repeat length and 2.6% heterozygosity with GenomeScope^19^ (Table S2). The extent of the repeat and thus the total genome size seems to be underestimated since our repeat analysis identified 51.1% to be the total repetitive content (Table S3). Although the genome seems to still contain at maximum 11.2% of uncollapsed heterozygosity as suggested by the BUSCO duplication rate, we consider the genome assembly to be comprehensive, in light of the cDNA-seq mapping rate (96.8% ± 0.7).

**Table 1:** Summary statistics of *A. ventricosus* draft genome.

Genome	
Scaffold number	3,00,721
Total scaffold length (bp)	3,65,66,29,030
Average scaffold length (bp)	12,159
Longest scaffold length (bp)	93,35,346
Shortest scaffold length (bp)	609
N50 (bp) (# of scaffolds in N50)	59,619 (#9,086)
N90 (bp) (# of scaffolds in N90)	4,039 (#126,643)
BUSCO^a^	
Complete BUSCOa (%)	90.10
BUSCO^b^	
Complete BUSCOs (%)	91.18
Complete and single-copy BUSCOs (%)	80.20
Complete and duplicated BUSCOs (%)	11.00
Fragmented BUSCOs (%)	3.80
Missing BUSCOs (%)	5.00
**Genes**	**v3**
Number of ORF	2,78,945
Estimated gene number^c^	29,380
Genes with BLAST matches to Uniprot & Pfam domain (E-value < 1.0e-5)^c1^	20,735
Number of expressed genes (TPM > 0.1)^c2^	23,412
Genes with Gene Ontology terms	19,974
tRNAs	10,558
rRNAs	248
BUSCO completeness (%)^a^	91.75
BUSCO completeness (%)^b^	93.06

^a^Eukaryota database, ^b^Arthropoda database.

^c^Union of BLAST hit genes (c^1^) and expressed genes (^c2^).

The gene content within the *A. ventricosus* genome was analyzed using cDNA sequencing. The cDNA was constructed from RNA samples from five independent whole bodies and six silk abdominal silk glands (Table S4). Approximately 35 million 150-bp paired-end reads were sequenced in each sample. Based on a gene model constructed using cDNA sequencing data, 277,986 open reading frames (ORFs) were predicted, and up to 29,380 (conservatively 14,767) protein-coding genes were estimated based on the expression level and functional annotation (Figs S1, S2). The quality of the predicted gene set was estimated by the BUSCO completeness score, and the test with the Arthropoda gene model showed 93.06% (Table 1).

### Full spidroin gene set in *A. ventricosus*

**Figure 1:**
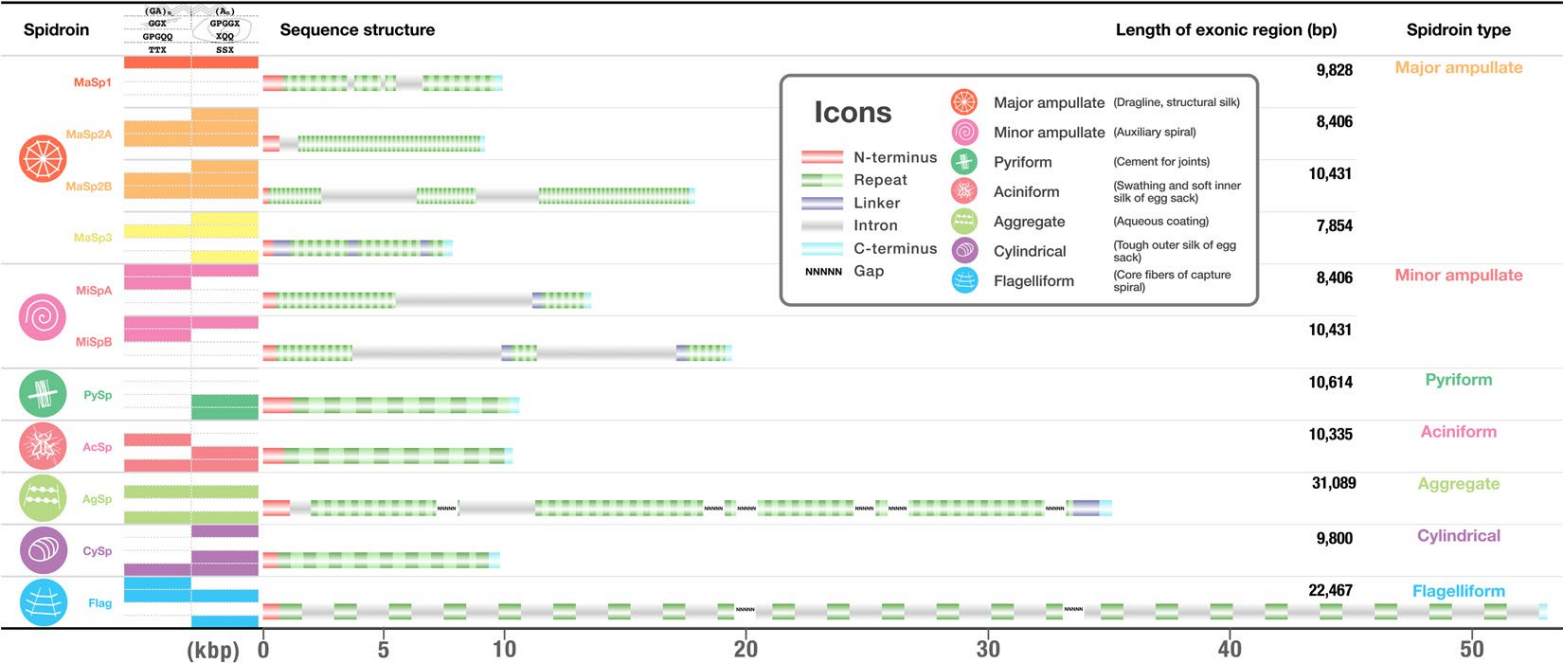
Catalogue of spidroins in *A. ventricosus*. This summary table shows the spidroin genic characters and structures obtained from the *A. ventricosus* genome. The icons in the first column represent spidroin type, and the specific colour is used for each type. The colour panel at second column represents the motif variety in the repetitive domain. The motif box includes β-sheet ((GA)_n_ and A_n_), β-turn (GPGGX, GPGQQ and XQQ), 3_10_ helix (GGX), and spacer. Sequence structure column shows the N/C-terminal and repetitive domains, and each size is drawn to scale. The number of stripe in the repetitive domain also reflects the number of repeats.

First candidates of the spidroin gene were computationally screened based on sequence similarity. Candidates were then manually curated using the cDNA (see methods), unamplified long nanopore genomic DNA reads, and direct-RNA sequencing^20^ without reverse transcription or amplification steps. The final gene set was summarised into eleven spidroin genes belonging in seven orthologue groups (Fig. 1 and Table S5).

With the exception of *Flag* and *AgSp* gene, the full-length of all spidroin genes without gaps were newly determined. Identified spidroins were highly diverse in the sequence length, exon-intron architecture, and repetitive structures (Fig. 1). The majority of the spidroin genes contained intronic regions, and linker domains were also found between repetitive units. The longest spidroin gene is the *Flag* gene constituted by three contigs, and the length of the total exonic regions is approximately 22.5 kb. Even the shortest *MiSpB* gene is 7.5 kb in length. Two paralogues were found for both *MaSp2* and *MiSp*, with different gene structures and the number of repetitive units (Fig. 1). Furthermore, *MaSp2A* and *MaSp2B* were tandemly arranged within the same contig (Fig. S3). As previously described^21–23^, distinct repetitive motifs were reconfirmed in each spidroin gene (Fig. 1). There were no common repetitive motifs in all spidroin gene family, and this variety presumably reflects the diverse functionality of different spidroins.

The comparison with previously isolated full or partial sequences in *A. ventricosus* supported the accuracy of our spidroin gene set. The *AcSp* gene sequence almost entirely matched the previously reported one^24^ (Accession no. MG021196; Fig. S4). Regarding the *Flag* gene, the only known C-terminus region^25^ (Accession no. EF025541) was clearly aligned with our isolated gene sequence (Fig. S5). The *CySp* and *MiSp* genes had slightly longer sequences than known sequences obtained from the PCR approach^26,27^ (Accession no. MF192838 for *CySp*; Fig. S6, JX513956 for *MiSp*; Fig. S7), reconfirming the problem of PCR-based amplicon sequencing of spidroins, and the advantage of unamplified single molecule approach.

### Full length sequence of a novel spidroin gene *MaSp3*

In addition to the above classical spidroin genes, the first full length of *MaSp3* gene, originally named by Collin and colleagues^28^ partially reported in *Argiope argentata* and *L. hesperus* was also isolated.

**Figure 2:**
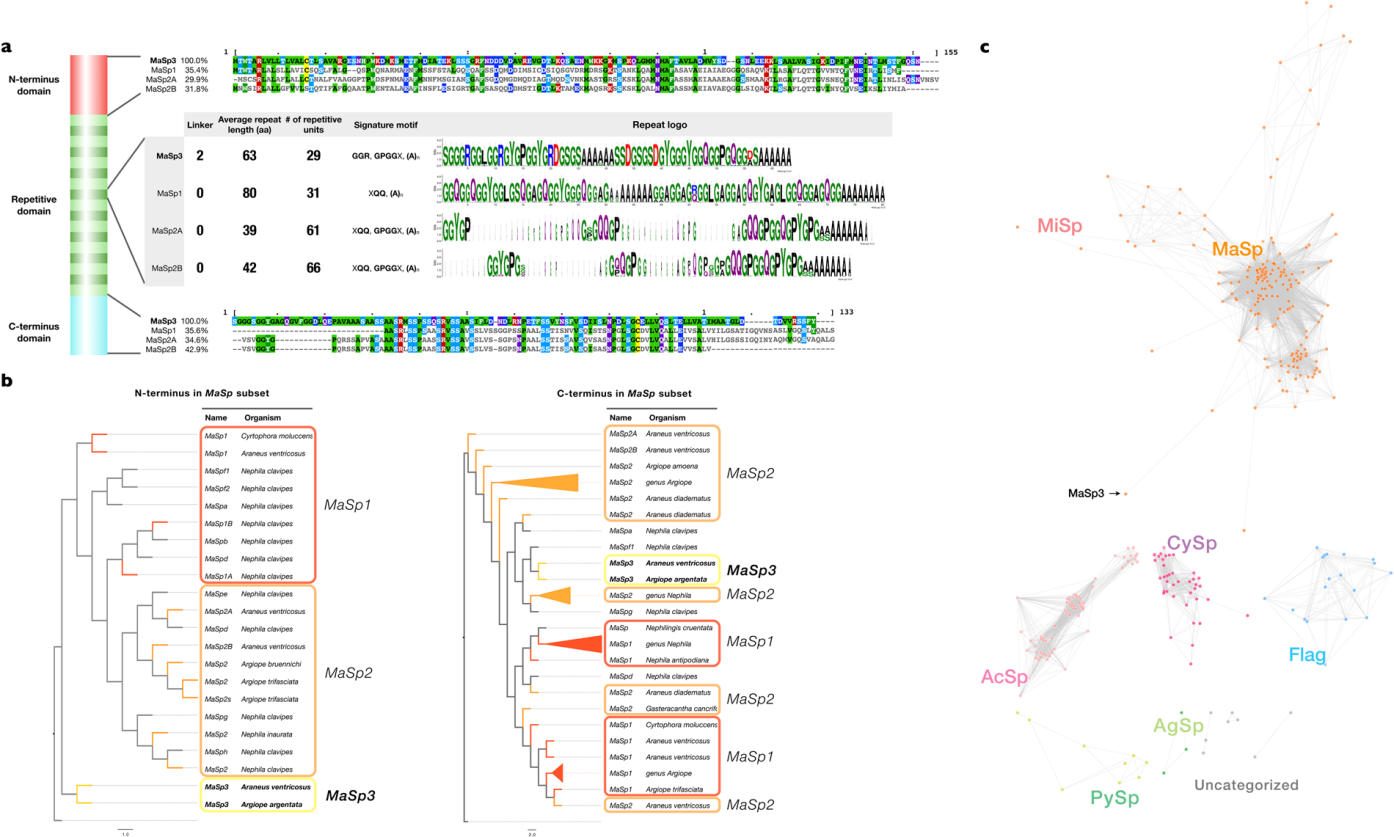
Characteristics of *MaSp3* gene in various scales from gene family to global spidroin category. (**a**) Alignment result and the sequence logo among *MaSp* subsets. (**b**) Clustering of the N/C-terminus among *MaSp* subsets in closely related spiders. The similarity of N-terminus demonstrated clearer clusters than did the C-terminus domain. **(c)** Global spidroin category in the superfamily Araneoidea. This spectral clustering was performed using every partial and complete sequence of the spidroin genes. Node colours represent the spidroin gene subsets.

The N/C-terminus domain sequences of the *MaSp3* gene show only limited homology to other *MaSp* family genes in *A. ventricosus*, suggesting distinct divergence in this paralogue (Fig. 2a). When the terminal domains were clustered among the published *MaSp* genes in the family Araneidae, the N-terminus domains clearly cluster into three *MaSp* paralogs, independent of taxonomy (*MaSp1, MaSp2, and MaSp3*, Fig. 2b). Such distinct clustering was not observed for the C-terminus domain of all three paralogues, not just *MaSp3*. Although previous reports suggested the lack of common *MaSp* motifs (A_n_ and GPG) in the *MaSp3* repetitive domain in *A. argentata*^28^, the *A. ventricosus MaSp3* actually possesses these motifs. In contrast, its repetitive domain has a highly frequent arginine motif “GGR”, and the motif has never been reported as a spidroin motif (Fig. 2a).

To further confirm the distinction of *MaSp3* as a paralog of *MaSp*, we prepared a global view of spidroins in the superfamily Araneoidea. A full length of our spidroin gene set was clustered with previously reported spidroin genes (Table S6) by spectral clustering^29^ based on a combination of multiple local sequence similarities to capture the combined sequence similarity/divergence of the N-terminus, repeats, and C-terminus regions (Table S7). Again, the result of spectral clustering confirms that *MaSp3* is a subset of the *MaSp* category (Fig. 2c).

### Phylogenetic origin of *MaSp3*

**Figure 3:**
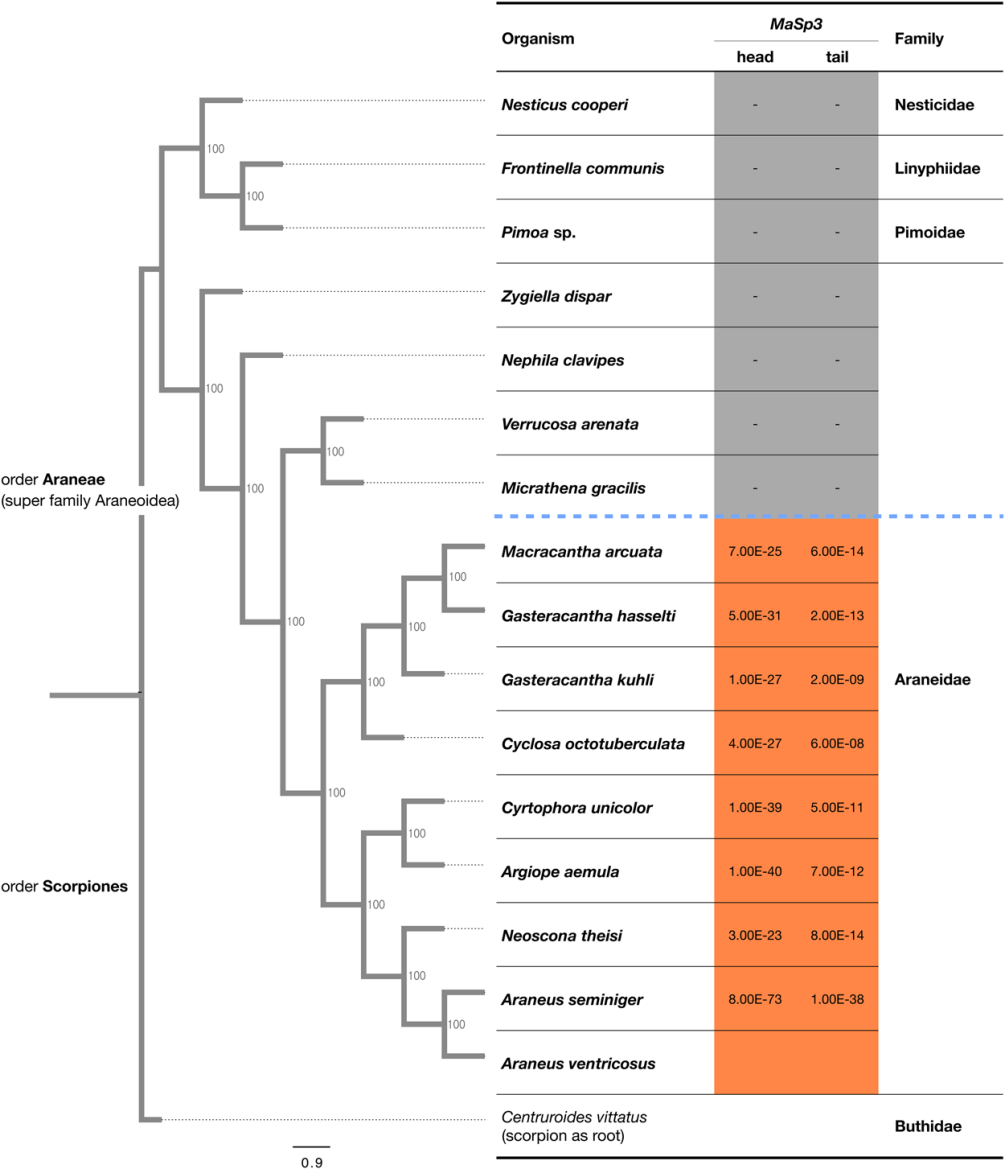
Phylogenetic location of *MaSp3* gene around Araneoidea. Phylogenetic tree based on the protein sequence of 4,934 orthologous genes in closely related spiders in superfamily Araneoidea. A scorpion was used as root. The e-values at the head and tail represent the result of BLAST search using N-terminus (head) and C-terminus (tail) 170 residues of *MaSp3* as the query. The orange boxes represent e-value < 1.0e-5.

To investigate when the *MaSp3* gene was evolutionarily acquired, we implemented a phylogenomic conservation analysis. A phylogenetic tree, including our *A. ventricosus* genome, was constructed based on a core orthologue gene set^30^, which was identified from assembled contigs with Araneids transcriptome data obtained from the NCBI SRA database (http://www.ncbi.nlm.nih.gov/sra). Moreover, to achieve higher resolution in the family Araneidae, we performed additional transcriptome analyses for five other spiders belonging to the family Araneidae (Tables S8–S10). We then constructed a phylogenetic tree expanding the family Araneidae and properly reflecting various previously reported trees^30,31^ (Fig. 3). The phylogenetic conservation of *MaSp3* was then mapped on the phylogenetic tree using a homology search of 170 amino acids of the N/C-terminus region. Furthermore, a conservation analysis among 163 spider transcriptome data showed that the *MaSp3* presents only in a part of Araneidae (Table S11), as the result of phylogenomic analysis, and strikingly, the *MaSp3* is only conserved in a subset clade within the family Araneidae. Therefore, the *MaSp3* gene in Araneidae seems to be acquired after the branching event from *Nephila*, *Micrathena*, and *Verrucossa* (Fig. 3). In light of these phylogenetic conservation patterns and the lack of GGR motif, partial sequence previously reported to be *MaSp3* in *L. hesperus* (family Theridiidae) is likely to be a different paralogue of *MaSp* family.

### Proteome and expression profiling within dragline silk

**Figure 4:**
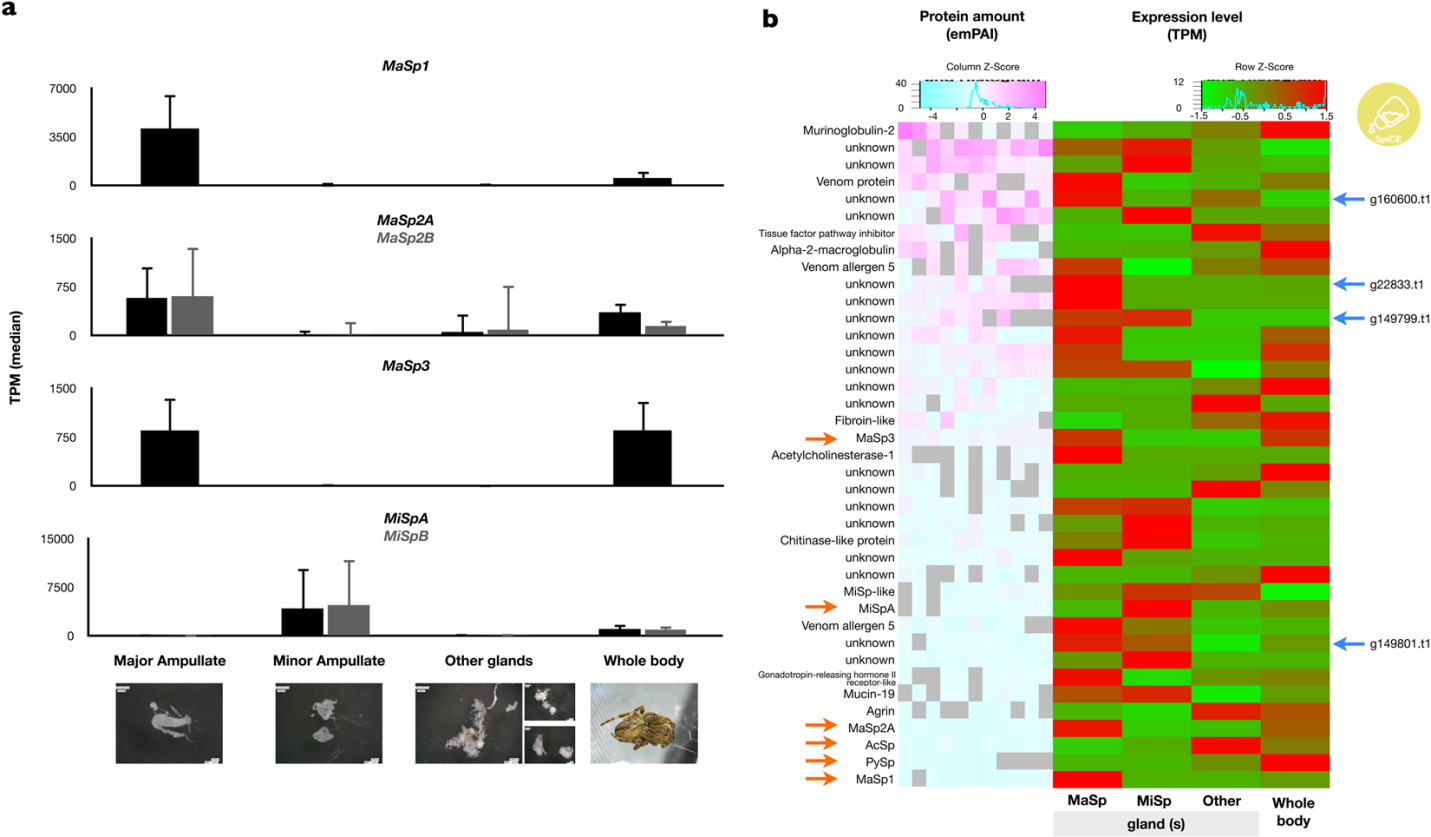
Expression and proteome analysis in dragline silk. (**a**) Gene expression level of the spidroin genes in the whole body and each abdominal silk gland with three biological replicates per sample. The pictures in each graph are representative images of the samples. Other glands include multiple silk glands other than major ampullate and minor ampullate. The expression profiles of other spidroin genes were described at Fig. S8. (**b**) The left heat map of proteome of dragline silk in *A. ventricosus*. Orange arrows indicate the spidroin proteins. Right: heat map of expression of corresponding genes. Blue arrows indicate the SpiCEs.

The contribution of *MaSp3* to the composition of dragline silk was investigated by transcriptomic and proteomic approaches. The expression profiling was performed from cDNA-Seq samples in five whole bodies and abdominal silk glands (major ampullate, minor ampullate, and others). As expected, the *MaSp3* gene was highly expressed in the major ampullate gland but is hardly observed in other glands (Figs 4a and S8, S9). This expression pattern was very similar to other profiles in *MaSp* family genes (*MaSp1*, *MaSp2A*, and *MaSp2B*).

The proteomic analysis was performed using dragline silks directly collected from five female *A. ventricosus* (Table S4). The spectra obtained from nanoLC-MS/MS analysis were annotated by a MASCOT search against our *A. ventricosus* genome database. Proteome analysis revealed that dragline silk yielded approximately 100 proteins on average (Table S12). Proteins shared in all samples were summarised at Fig. 4b. As expected from a previous study^32^, we confirmed the presence of the MaSp and AcSp proteins in dragline silk (Fig. 4b). Proteome analysis demonstrated that the peptides from MaSp3 specific linker domain were detected (Fig. S10), and the MaSp3 peptides were the most abundant in the dragline silks (Fig. 4b).

### Relationship between the mechanical property and protein composition

**Figure 5:**
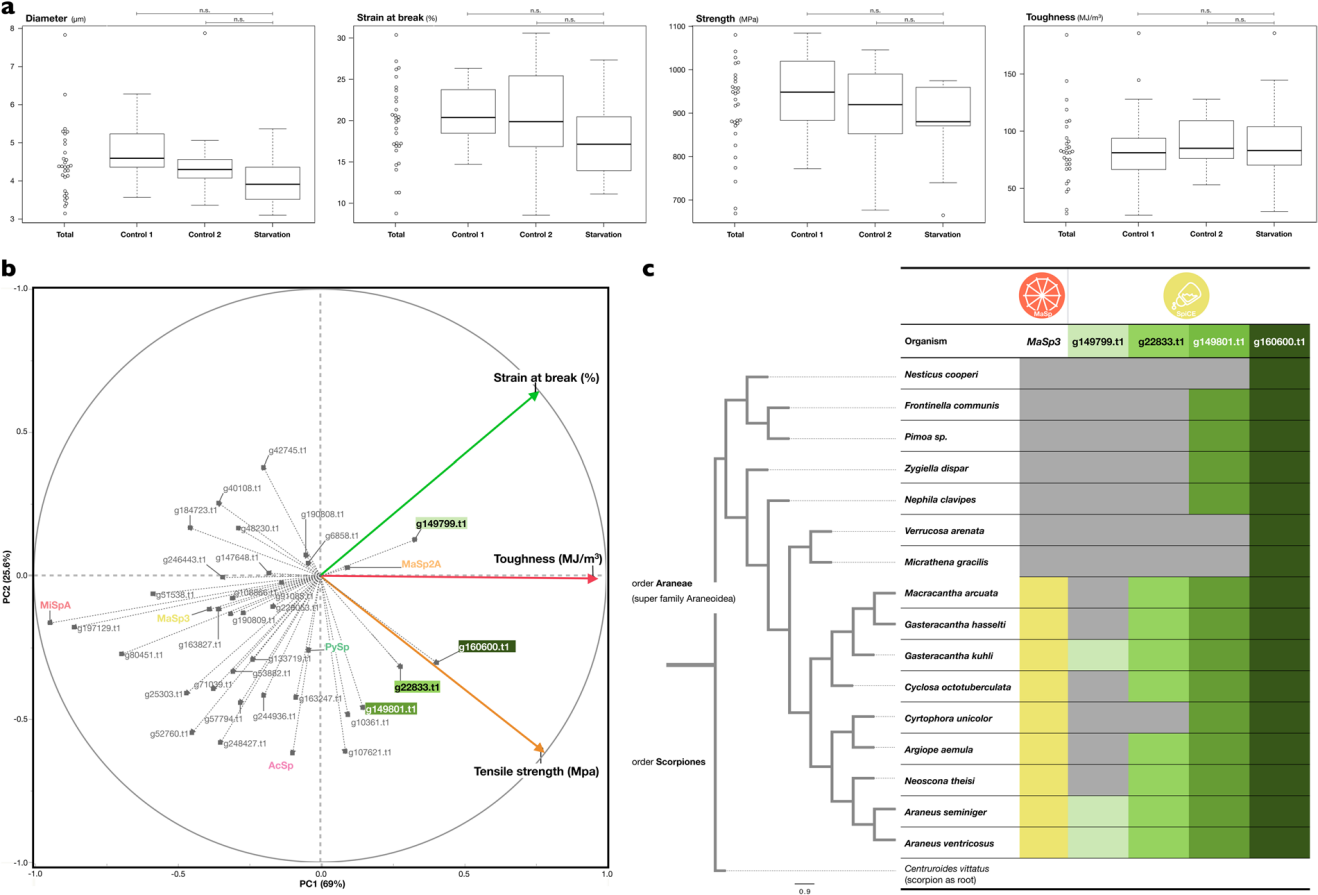
Mechanical properties of dragline silk in *A. ventricosus* and phylogenetic trait. (**a**) Relationship between the mechanical properties and nutrition conditions. There are no significant differences. (**b**) PCA score plot of the mechanical properties (Strain at break, toughness, and tensile strength) and proteins included in dragline silk. (**c**) Phylogenetic position of *MaSp3* and four genes especially associated with the mechanical property. Coloured boxes represent *MaSp3* and SpiCEs found in transcriptome data of organisms.

We then investigated the impact of dragline components on dragline mechanical properties. Dragline silks used for mechanical property testing were obtained from 10 specimens of female *A. ventricosus* with various nutrition conditions. In this study, the following mechanical properties of the silks were measured: thread diameter (µm), strain at breaking (%), ultimate strength (MPa), and toughness (MJ/m^3^). These physical properties of dragline silk were measured on an Instron 3342 materials tester (Instron, Inc.) and summarised in Fig. 5a. As shown in Fig. 3b, the average values were diameter = 4.47 ± 0.96 µm, strain at breaking = 19.55 ± 5.02%, ultimate strength = 906.9 ± 100.9 MPa, and toughness = 84.28 ± 31.91 MJ/m^3^. However, there was high variance between the samples. Previous studies have reported that prey variation alters the silk composition^15,33,34^. Therefore, we first examined the effect of the nutrition condition on silk mechanics (Fig. S12), but the mechanical properties did not seem to be affected by these nutrition conditions (Fig. 5a and Tables S12, S13). Furthermore, the nutritional manipulations did not have an effect on protein composition in dragline silk (Table S12). Since the silk mechanical properties nor its components were independent of the drastic change in the nutrition conditions from being starved or directly after feeding, we investigated the direct relationship between silk components and mechanical properties.

The MaSp3 was constitutively the most abundant in dragline silk among six spidroins (represented by orange arrow in Fig. 4b), and PCA did not show direct contribution of MaSp3 to the mechanical properties of dragline silk (Fig. 5b). Therefore, genetic approaches to knock down MaSp3 expression or synthetic approaches would be necessary to elucidate the specific contribution of MaSp3 within *A. ventricosus*, due to the little intraspecies differences of MaSp3 abundances. On the other hand, among family Araneidae species, dragline silks in genus *Araneus* and *Argiope* with MaSp3 have higher toughness and tensile strength than the one in genus *Nephila* without it^35^, thus it is suggested that the MaSp3 may account for the interspecies differences. On the other hand, low molecular weight novel spider silk-constituting element, named SpiCE (Figs 4 and 5), was shown to be pivotal in intraspecies differences. SpiCE proteins contributing to toughness or tensile strength were found, and these four proteins (coded by g22833.t1, g160600.t1, g149801.t1, and g149799.t1) were highly expressed exclusively in the major ampullate gland (Fig. 4b, blue arrows). The conservation pattern of these silk-related proteins was investigated among the superfamily Araneidae based on sequence homology. Although g160600.t1 and g149801.t1 were widely conserved, two other genes were not uniformly conserved in the whole body transcriptomes of closely related spiders. Of note, the conservation pattern of g22833.t1 gene was very similar to that of the *MaSp3* gene (Fig. 5c), suggesting a possible association between the two proteins.

## Discussion

The extreme length and repetitive structure of the spidroin genes posed a challenge for comprehensively sequencing of these genes within a genome due to limitations in the short read based assembly and difficulty in correct amplification of long repeats with PCR. By combining multiple sequencing approaches, including nanopore long reads of unamplified DNA and RNA single molecules, this paper first demonstrated a working strategy to obtain the full spidroin gene set. Using the obtained genomic information, we successfully identified the first full length sequence of *MaSp3* and several other SpiCE proteins that possibly contribute to the mechanical properties of dragline silk through a multiple omics approach.

The *A. ventricosus* genome data enabled comparative genomics in Araneoids. Our hybrid sequencing could present the approximately 3.5 Gb *A. ventricosus* genome with the full spidroin gene set, at high BUSCO coverage and comprehensive cDNA-Seq mapping rates. The 10X GemCode barcoded synthetic long read assembly provided accurate and comprehensive foundations of genome assembly, while this technology alone was not able to complete the repetitive spidroin regions. Hybrid sequencing using nanopore long reads from unamplified single molecule genomic DNA and direct-RNA sequencing^20^ without reverse transcription finally allowed the completion of the full length spidroin genes (Table S1). Current molecular biology techniques on DNA mostly rely on PCR; however, PCR amplification of long repetitive spidroins very often result in chimeric sequences or amplicons of different lengths. Comparison of previously reported sequences (*CySp* and *MiSp* of *A. ventricosus*) using spidroin amplicons with our unamplified single molecule sequences clearly shows the difficulty of obtaining accurate full-length sequence by amplicon-based approach. We believe it is critical to use single molecule long read sequencing techniques without amplification for such difficult sequences, including but not limited to spidroins. Genome sequencing and gene prediction analysis revealed that there are seven spidroin gene orthologue groups in common with other Araneoids. Because of the better continuity of our assembly, we can accurately identify the existence of multiple paralogs, locate the introns within the spidroins, and study the gene order of the spidroins. Interestingly, the two paralogs of *MaSp2* are tandemly co-localised within the *A. ventricosus* genome, and such localisation may have implications on spidroin expression regulation.

One of the key findings from the genome sequencing was the identification of new paralogs of *MaSp* type spidroin *MaSp3*. The existence of this paralogue has been suggested from partial terminal domains obtained from target capture sequencing^36^, but the full length sequence was not previously reported. According to the phylogenetic analysis with N/C-terminus domains (Fig. 2b), while the N-terminus domains were distinct in *MaSp1-3*, the C-terminus domains were not clearly separated. Therefore, it had been difficult to recognise the *MaSp3* gene and its N-terminus through partial sequencing of target captured cDNA. Phylogenetic analysis around the superfamily Araneoidea based on orthologous genes, with novel transcriptome data of five spiders generated in this work to increase the resolution of the phylogeny, revealed that *MaSp3* might be relatively recently acquired after the branching event from genus *Nephila*. The genus *Nephila* was formerly classified into another family (Nephilidae), and Blackledge and colleagues previously showed that the genus *Araneus* and *Nephila* were strictly categorised into different clades in the aspect of web architecture^31^. The conservation pattern of *MaSp3* in a subclade of Araneidae excluding *Nephila* mirrors such observation, and this synapomorphy may provide clues to the different orb web characters and mechanical properties in Araneidae. Furthermore, our proteome and transcriptome analyses showed that *MaSp3* is one of the most important constituents of dragline silk (Figs 2 and 4). A detailed biochemical analysis regarding the unique repeat structure of *MaSp3* would be interesting future work in this direction.

New spidroin subgroup candidates have been reported in previous studies^13,28,37^, but thus far, the classifications have been based on inter-species comparison of terminal domains or repeats. Although many unknown spidroin-like genes were also found in *N. clavipes*^13^, many remained unknown due to very weak conservation of the N-terminal domain, and the observation is based on amplicon-based sequencing. Since we now have the first plausible complete sequence set of a single species, we can utilise these data for global clustering, considering local sequence similarity information in both terminal sequences, repeats, and linkers by means of spectral clustering (Fig. 2c). This clustering approach clearly distinguishes different spidroin subtypes and demonstrates that some subtypes such as *AcSp* tend to subcluster among taxonomic neighbours, *i.e*., clade-specific adaptation is observed. Additionally, *MaSp, MiSp*, and *CySp* are more conserved. The new spidroin found in this work, *MaSp3*, was classified into the *MaSp* group but was sufficiently distant from the *MaSp1* and *MaSp2* paralogue subclusters. Based on this clustering, previously reported unknown fibroin-like proteins (Sp-907 and Sp-74867)^13^ in *N. clavipes* did not belong to any categories. This global clustering approach seems to clearly show the subtypes of spidroins and may be utilised as a complemental method to existing classifications.

Our high quality genome sequence also served as a reference for sensitive and reproducible proteome analysis. This study detected and confirmed many proteins within dragline that have been previously reported, such as alpha-2-macroglobulin 2, which was found as an exclusive protein in major ampullate silk glands in a western black widow spider (*L. hesperus*)^32^. Peroxidasin has been found at the boundary of a peripheral layer and the silk fibre core in the caddisfly silk fibre^38^, and its presence in spider dragline may serve similar functionality. On the other hand, we did not confirm the contribution of CRP (Cysteine Rich Protein), which has been reported as a protein to form structural constituents of fibres in a cobweb spider (family Theridiidae)^39,40^. Four CRP-like proteins were found in our *A. ventricosus* genome, and they were similar to CRP5 protein (Accession ADV40350.1). Although they were expressed in the whole body or abdominal spidroin glands (Fig. S12), the peptides were not found in dragline silk.

The presence of our curated spidroins was clearly confirmed in the proteome analysis, and we identified new genes possibly associated with the extraordinary mechanical properties of dragline silk. These genes are much shorter than the spidroins, and they are not similar to spidroins (Fig. S13). However, these genes were both highly expressed specifically in the major ampullate gland and are highly abundant as protein constituents within the dragline. The abundance of these proteins showed correlation with the toughness of the dragline, and one of them, g22833.t1, showed similar clade-specific conservation patterns with *MaSp3* (Fig. 5c). The abundance estimate of the protein in this work was based only on the calculated emPAI values of the nanoLC-MS/MS analysis and should be further confirmed by other forms of direct quantification such as Western blotting. However, spider silk may be more highly complex than previously expected, with clade-specific duplication, the divergence of spidroin paralogues and the presence of other SpiCEs.

Our *Araneus* genome assembled by the hybrid of synthetic and single molecule long reads revealed the full length of a new spidroin. Due to the recent development of genomic technologies, many unknown spidroin genes have started to be discovered from spider genome or transcriptome data. We consider that such novel spidroin finding may occur among all spider clades. The MaSp3 found in genus *Araneus* and closely related spiders is a clade-specific spidroin, presumably correlating with the ability of these clade of spiders to produce large orb webs, requiring the extra toughness they exhibit. We have also identified non-canonical silk constituents that do not show homology to existing spidroins that we termed SpiCE, and according to our mechanical property analysis (Fig. 5), these proteins also contribute to the overall toughness of the silk. It is also interesting that the conservation patterns of many SpiCE proteins mirror that of MaSp3. This perspective suggests the possibility for a greater diversity of spidroin evolution and their related proteins that may be clade-specific to suit specific ecological adaptations (Fig. S14). Although MaSp is especially apt to be the research target and many paralogues have been observed^13,28^, the variety is also observed in prey capture thread. Some cribellate spiders have characteristic prey capture threads such as cribellate silk^41^ and pseudoflagelliform silk^42^, and recent studies have shown that these genes are categorized into specific spidroin types^43,44^. Therefore, the spidroin repertoire represents the behavioral and ecological variety of spiders, and more new spidroins or other silk constituents are likely to be found in the future.

## Methods

### Spider sample preparation

Spider specimens were initially identified based on morphological characteristics, and further confirmed by the transcriptome assembly of cytochrome c oxidase subunit 1 (*COX1*) in the Barcode of Life Data System (BOLD: http://www.barcodinglife.org). *A. ventricosus* (L. Koch, 1878) samples were collected from Akita, Yamagata, and Kumamoto Prefecture, Japan (December 2015). The samples were stored in a centrifuge tube and transported live back to the laboratory. *A. ventricosus* was kept in plastic containers PAPM340 (RISUPACK CO., LTD.) inside the laboratory with an average room temperature of 25.1 °C and 57.8% of humidity for approximately 2 weeks before the experiment. Light was controlled by an automatic system under a 12-h light/dark cycle. *A. ventricosus* was fed one cricket (*Gryllus bimaculatus* - commercially purchased from mito-korogi farm) once every 2 days. Water was provided once every day by softly spraying inside the plastic container. According to a previously reported standardised protocol of field sampling^45^, immediately upon arrival at the laboratory, *A. ventricosus* were immersed in liquid nitrogen (LN2) for whole body cDNA, RNA, genome sequencing and stored at −80 °C. Each gland tissue sample was dissected after anaesthetising with CO2, washed with phosphate buffered saline (PBS) and stabilised in RNAlater (Life Technologies). Photos of the dissected glands were taken immediately using VHX-5000 (Keyence), and the samples were flash frozen at −80 °C. Three biological replicates were separately prepared for all gland samples. *Neoscona theisi*, *Gasteracantha kuhli*, *Argiope aemula*, *Cyrtophora unicolor*, *Acanthepeira* sp., and *Zygiella dispar* samples were used only for cDNA sequencing. Sampling location data are described in Table S4.

### HMW (high molecular weight) gDNA extraction

gDNA was extracted from four adult *A. ventricosus* whole bodies using Genomic-tip 20/G (QIAGEN) basically following the manufacturer’s protocol. To keep the HMW quality, every step was performed as gently as possible. Flash frozen spider specimens were separated into each body segment, and gDNA was extracted from the cephalothorax and legs. The specimens with the abdomen removed were homogenised using BioMasher II (Funakoshi) and mixed with 2 ml of Buffer G2 (QIAGEN), including 200 µg/ml RNase A. After addition of 50 µL Proteinase K (20 mg/mL), the lysate was incubated at 50 °C for up to 12 h on a shaker (300 rpm). The lysate was centrifuged at 5,000 × g for 5 min at 4 °C to pellet the debris, and the aqueous phase was loaded onto a pre-equilibrated QIAGEN Genomic-tip 20/G (QIAGEN) by gravity flow. The QIAGEN Genomic-tip 20/G (QIAGEN) was then washed three times and the DNA was eluted with high-salt buffer (Buffer QF) (QIAGEN). The eluted DNA was desalted and concentrated by isopropanol precipitation and resuspended in 10 mM Tris-HCl (pH 8.5). Extracted gDNA was quantified using a Qubit Broad Range (BR) dsDNA assay (Life Technologies) and qualified using TapeStation 2200 with genomic DNA Screen Tape (Agilent Technologies).

### Library preparation for genome sequencing

For synthetic long-read sequencing, 10 ng purified HMW gDNA was used. The library preparation was performed with GemCode using a Chromium instrument and Genome Reagent Kit v2 (10X Genomics) following the manufacturer’s protocol. Library quality was estimated by TapeStation 2200 with D1000 Screen Tape (Agilent Technologies).

For nanopore long-read sequencing, the libraries were completed following the 1D library protocol (SQK-LSK108, Oxford Nanopore Technologies). The HMW gDNA applied to library preparation was purified by >10 kb size selection using a BluePippin (Sage Science) with 0.75% Agarose Gel Cassette.

### Total RNA extraction

Total RNA was extracted using a spider transcriptome protocol, as previously described^45^. Flash frozen spider specimens were immersed in 1 mL TRIzol Reagent (Invitrogen) and homogenised with a metal cone using the Multi-Beads Shocker (Yasui Kikai). Following phase separation with the addition of chloroform, the upper aqueous phase containing extracted RNA was further purified using a RNeasy Plus Mini Kit (Qiagen) automated with QIACube (Qiagen). The quantity of purified total RNA was measured with NanoDrop 2000 (Thermo Scientific) and Qubit Broad Range (BR) RNA assay (Life Technologies), and the integrity was estimated by electrophoresis using TapeStation 2200 with RNA Screen Tape (Agilent Technologies).

### Library preparation for cDNA and direct-RNA sequencing

The cDNA library was constructed using a standard protocol of the NEBNext Ultra RNA Library Prep Kit for Illumina (New England BioLabs). Approximately 100 µg total RNA was used for mRNA isolation by NEBNext Oligo d(T)_25_ beads (skipping wash step with Tris buffer). The first and second strands of cDNA were synthesized using ProtoScript II Reverse Transcriptase and NEBNext Second Strand Synthesis Enzyme Mix. Synthesized double-stranded cDNA was end-repaired using NEBNext End Prep Enzyme Mix and ligated with a NEBNext Adaptor for Illumina. After the USER enzyme treatment, cDNA was amplified by PCR with the following conditions (20 *μ*L cDNA, 2.5 *μ*L Index Primer, 2.5 *μ*L Universal PCR Primer, 25 *μ*L NEBNext Q5 Hot Start HiFi PCR Master Mix 2X; 98 °C for 30 s and 12 cycles each of 98 °C for 10 s, 65 °C for 75 s and 65 °C for 5 min). When the total RNA volume was less than 10 ng, the library was prepared using SMART-Seq v4 Ultra Low Input RNA Kit for Sequencing (Clontech) according to the manufacturer’s protocol, with subsequent fragmentation and Illumina library preparation with Hyper Plus Kit (Kapa Biosystems). For direct-RNA sequencing, 500 ng of mRNA was prepared using the NucleoTrap mRNA Mini Kit (Clonetech) and the libraries were completed following manufacturer’s protocol (SQK-RNA001, Oxford Nanopore Technologies).

### Sequencing

The GemCoded genome library was prepared with Chromium (10X Genomics), and cDNA sequencing was performed with a NextSeq 500 instrument (Illumina, Inc.) using a 150-bp paired-end read with a NextSeq 500 High Output Kit (300 cycles). Sequenced reads were assessed with FastQC (v0.10.1: http://www.bioinformatics.bbsrc.ac.uk/projects/fastqc/).

Nanopore genome and direct-RNA sequencing was performed using a MinION device with a total of eight v9.4 SpotON MinION flow cells (FLO-MIN106, Oxford Nanopore Technologies). The data sets obtained from this study were deposited and are available at the DNA Data Bank of Japan (DDBJ: http://www.ddbj.nig.ac.jp/) Sequence Read Archive with Accession no. DRA006821 and DRA006933.

### *De novo* genome assembly and error correction

The NextSeq reads prepared by Chromium were assembled with Supernova (v. 2.0.0). Supernova assembly was further scaffolded and gap closed using the MinION reads with PBJelly^46^ and corrected using the NextSeq reads with two rounds of Pilon^47^.

To validate the genome assembly, we calculated genomic coverage and genomic completeness. First, the DNA-Seq data was mapped to the genome with BWA MEM (Burrows-Wheeler Alignment v0.7.12-r1039)^48^, and after Sequence Alignment/Map (SAM) to BAM conversion with SAMtools (v 1.3)^49^, the genome coverage was calculated with QualiMap bamqc^50^ v2.2. Second, the genomic completeness of the Supernova assembly was validated with BUSCO (Benchmarking Universal Single-Copy Ortholog, Eukaryote and Arthropoda lineage gene set, -m genome) version 2.01^51^.

### Gene prediction and annotation

The gene model created by the cDNA-seq data mapping with HISAT2 version 2.1.0^52^ and BRAKER version 1.9^53^ was used for gene prediction. To annotate the predicted gene models, we submitted the amino acid sequences to similarity searches using BLAST against UniProt (Swiss-Prot and TrEMBL)^54^, and HMMER version 3.1b2^55^ searches against Pfam-A^56^. The protein-coding gene number was estimated using the intersection or union of transcript abundance (see below) and the functional annotations of UniProt and Pfam (Fig. S1). The tRNA and rRNA genes were also predicted with tRNAscan-SE version 1.3.1^57^ and Barrnap (https://github.com/tseemann/barrnap), and conducted repeat identification with RepeatModeler (http://www.repeatmasker.org/RepeatModeler/) and RepeatMasker (http://www.repeatmasker.org).

### Spidroin gene curation based on the hybrid assembly

The spidroin gene curation was carried out by the hybrid assembly with the short and long reads. The conceptual diagram is shown in Fig. S15. Short reads obtained by Illumina sequencing is typically assembled using a de Bruijn graph, but such assembly is not feasible with the repetitive region. Therefore, we developed an original SMoC (Spidroin Motif Collection) algorithm. The SMoC algorithm first picks up the spidroin gene N/C-terminus candidates (non-repetitive region) with BLAST search from assembled genomic contigs, and repetitive regions from transcriptome assembly. These candidates are used as seed sequences for a screening of the short reads harboring an exact match of extremely large k-mer (approximately 100) up to the 5′-end, and the obtained short reads are aligned to constructs a PWM (Position Weight Matrix) on the 3′-side of the matching k-mer. Using very strict thresholds, seed sequence is extended based on the PWM until there is a split in the graph; i.e., neighboring repeat is not resolvable. By repeating this overlap-based extension algorithm, we can obtain the full length subsets of the repeat units. Finally, these pre-assembled repeat units are mapped onto error-corrected long reads obtained from the direct sequencing of the genomic DNA or RNA.

### Expression analyses

Transcript abundances were estimated by kallisto version 0.42.2.1^58^ in transcripts per million (TPM)^59^. Each transcriptome data set was obtained from the whole body and individual abdominal silk glands, and our *A. ventricosus* genome and predicted genes were used as the references.

### Phylogenetic analyses

The phylogenetic trees for the N, C-terminus domains of spidroin genes in the family Araneidae (Fig. 2b) were constructed using known domains (Table S6). N, C-terminus domains were determined by BLASTP. The phylogenetic tree in Fig. 3 was constructed using the existing transcriptome data (Table S8) collected via the NCBI SRA database (http://www.ncbi.nlm.nih.gov/sra), in addition to newly sequenced transcriptome data in *Neoscona theisi* (DRR129306), *Gasteracantha kuhli* (DRR129307), *Argiope aemula* (DRR129308), *Cyrtophora unicolor* (DRR129309), *Zygiella dispar* (DRR129310), *Araneus seminiger* (DRR129311), and *Cyclosa octotuberculata* (DRR129312) according to transcriptome analysis method as described above. Furthermore, in addition to the above samples, the 156 spider transcriptome data sets collected by Fernandez and colleagues^60^ were assembled and used for the comprehensive *MaSp3* gene conservation analysis (Table S11). The *de novo* transcriptome assembly was performed using Bridger^61^, with the following options: pair_gap_length = 0 and k-mer = 31. The assembled contigs were validated with BUSCO^51^. The 4,934 spider-specific gene set previously used in spider phylogenetic tree^30^ was obtained from assembled transcriptome contigs using HMMER version 3.1b2^55^.

Collected orthologue genes were aligned with MAFFT version 7.309^62^ (mafft -auto–localpair–maxiterate 1,000) and then trimmed with trimAl version 1.2rev59^63^. Bootstrap analysis was performed using RAxML version 8.2.11^64^, and the phylogenetic tree was drawn using FigTree version 1.4.3 (http://tree.bio.ed.ac.uk/software/figtree/).

### Dragline silk collection and proteome analysis

Dragline silk was reeled directly from adult *A. ventricosus* restrained using two pieces of sponge and locked with rubber bands (avoiding any kind of harm to the spider). First, dragline silk was meticulously removed from the spinnerets of the spider with a couple of tweezers and this silk was reeled using a reeling machine developed by Spiber Inc. in an aluminium bobbin at a constant speed of 1.28 m/min for 1 h.

A silk sample was gently washed with 100 µL Base buffer [50 mM Ammonium carbonate in distilled water] with 0.1% SDS per 0.5–1.0 mg of silk at RT for 1–2 min. After the supernatant removal, silk samples immersed into 46 µL Base buffer and 4 µL 500 mM DTT (Dithiothreitol) mixture. The silk solution was incubated for 1 h at 60 °C and left to stand until cool at RT. The supernatant was discarded, and 46 µL Base buffer and 4 µL 500 mM IAA (Iodoacetamide) mixture were added. The silk solution was incubated for 30 min at RT in dark, and the supernatant was discarded. Spider silk was washed with Base buffer three times. Peptide digestion of the washed silk sample was performed by 50 µL trypsin (10 ng/µL) at 37 °C overnight. Digested peptides were mixed with 250 µL of 0.5% formic acid and incubated at RT for 15 min with a rotator. Peptide samples were desalted using MonoSpin C18 (GL Sciences) and dried at RT.

### Starvation test

The investigation of the impact of nutrition condition on the mechanical property was implemented by controlling the timing of the feeding. Over the course of two weeks, spiders were fed at day 1, day 4, and day 7, and dragline silks were sampled at day 2 (control 1: for 1 day after feeding), day 5 (control 2: for 1 day after feeding), and day 14 (starvation: for 1 week after feeding). This experiment was then replicated with 10 individuals (Tables S12, S13).

### Mechanical property of dragline silks

The silk was carefully removed from the aluminium bobbins (without adding too much stress to the silk). Samples of 2 cm were taken and located into a paper template where the silk was attached with cyanoacrylate CA-156 (CEMEDINE CO., LTD.). For each specimen used, 10–15 testing pieces of silk were made. To determine the mechanical properties, we determined the diameter of the testing pieces by microscopic observation (Nikon eclipse LV100ND, lens 150x0). Three regions of the sample silk were selected and measured using NIS-Elements D 4.20.00 64-bit (Nikon). Tensile strength was measured using an Instron 3342 machine (Analysis program Bluehill lite Version 2.32 Instron 2005). The length of the testing pieces was set to 20 mm, and the testing speed was set to 10 mm/sec.

### Liquid chromatography mass spectrometry analysis

Each sample for proteome analysis was dissolved with 12 µL of 0.5% acetic acid 5% acetonitrile, and 5 µL of the solution was loaded on hand-made spray needle column (Reprosil-Pur C18 materials, 100 µm i.d. Dr. Maisch GmbH, Germany, 5 µm tip i.d., 130 mm length) using a HTC-PAL autosampler (CTC Analytics, Zwingen, Switzerland). The peptide fragments in the samples were separated through the column by reversed phase chromatography of linear gradient mode using UltiMate 3000 nanoLC Pump (Dionex Co., Sunnyvale, CA, USA). As the mobile phases, (A) acetic acid/water (0.5:100, v/v), (B) acetic acid/acetonitrile (0.5:100, v/v) and (C) acetic acid/dimethyl sulfoxide (0.5:100, v/v) were mixed keeping the flow rate of 500 nL/min. The composition was changed as follow: (A) + (B) = 96%, (C) = 4%, (B) 0–4% (0–5 min), 4–24% (5–65 min), 24–76% (65–70 min), 76% (70–80 min), and 0% (80.1–120 min). The separated peptides were ionized at 2400 V by positive electrospray method, injected into LTQ orbitrap XL ETD (Thermo Electron, San Jose, CA, USA) and detected as peptide ions (scan range: *m/z*300–1500, mass resolution: 60000 at *m/z* 400). Top 10 peaks of multiple charged peptide ions were subjected to collision-induced dissociation (isolation width: 2, normalized collision energy: 35 V, activation Q: 0.25, activation time: 30 s) to identify the amino acid sequence.

### Database search for protein identification

The peak lists were created from LC-MS raw data files with msconvert.exe provided from ProteoWizard^65^, and analyzed with Mascot server version 2.5 (Matrix Science, Boston, MA, USA)^66^ for identification of peptides and proteins in each samples. For the analysis, our *A. ventricosus* genome sequence was used with the following conditions: Precursor mass tolerance; 6 ppm, Product ion mass tolerance; 0.5 Da, Enzyme; Trypsin, Max missed coverages; 2, Fixed modification; carbamidomethylation at Cys, Variable modification; *N*-acetylation at protein N-term and oxidation at Met, Criteria for identification; p < 0.05 (MS/MS ion search).

### Computational analysis and statistics

All computational data curation, treatment, and basic analysis were performed using Perl custom scripts with the G-language Genome Analysis Environment version 1.9.1^67^. Statistical analyses were implemented using R package version 3.2.1. For the global spidroin category, the networks were constructed based on the sequence similarity among all the spidroin genes. The sequence similarity was calculated as a bit score with all-against-all BLASTP. The scores were normalised to 0.0–1.0 using a previously described normalisation method^68^. Using the normalised scores, all spidroin genes were clustered by spectral clustering with clusterx version 0.9.8^69^, and the clustering results were drawn by Cytoscape (v. 3.5.1), with a force-directed layout. Sequence logo was constructed by WebLogo 3^70^. PCA (principal component analysis) was calculated based on the correlation matrix and performed using JMP software version 13.2.0 (SAS Institute).

## Data Access

Raw sequence reads used for genome assembly and expression analysis have been submitted to DDBJ SRA (sequence read archive). Accession numbers of the whole body transcriptome are DRR129306 (*Neoscona theisi*), DRR129307 (*Gasteracantha kuhli*), DRR129308 (*Argiope aemula*), DRR129309 (*Cyrtophora unicolor*), DRR129310 (*Zygiella dispar*), DRR129311 (*Araneus seminiger*), DRR129312 (*Cyclosa octotuberculata*), and DRR129313-DRR129317 (*Araneus ventricosus*). Accession numbers of silk gland transcriptome in *Araneus ventricosus* are DRR138403-DRR138405 (major ampullate), DRR138406-138408 (minor ampullate), and DRR138409-138411 (other silk glands). Accession numbers of MinION sequencing for direct-RNA is DRR138400 (*Araneus ventricosus*) and direct-DNA is DRR138402 (*Araneus ventricosus*). Accession number of GemCoded sequencing in *Araneus ventricosus* is DRR138401 (Tables S4, S8). Assembled files have been submitted to figshare.com (Table S8). The whole genome sequence is available at the Whole-Genome Shotgun (WGS) database in DDBJ under accession number of BGPR01000001-BGPR01300721.

## Supplementary information

Supplementary Info
