# Supplementary material for: Orb-weaving spider Araneus ventricosus genome elucidates the spidroin gene catalogue
Source: Sci Rep. 2019 Jun 10;9:8380. doi: 10.1038/s41598-019-44775-2 (PMC6557832; doi:10.1038/s41598-019-44775-2)
Supplement: Supplementary file 1 — Supplementary Info [file 41598_2019_44775_MOESM1_ESM.docx]

**Supplemental Materials for**

Orb-weaving spider *Araneus ventricosus* genome elucidates the spidroin gene catalogue Nobuaki Kono, Hiroyuki Nakamura, Rintaro Ohtoshi, Daniel A. Pedrazzoli Moran, Asaka Shinohara, Yuki Yoshida, Masayuki Fujiwara, Masaru Mori, Masaru Tomita, Kazuharu Arakawa

**This Supplementary Material include:**

Figs S1 to S14, Tables S1 to S13

**Supplemental Figure S1**

Estimated gene number from expression level and functional annotation. Red area on the Venn diagram represents the intersection of the gene set (TPM > 0.1 and annotated by UniProt and Pfam), and contains 14,767 genes (conservative estimate of gene sets). Union of the circles represented in blue and red shows a more comprehensive estimate of up to 29,380 genes.

**Supplemental Figure S2**

Functional annotation of predicted genes in *A. ventricosus*. Comparison of GO term mapping distributions of *A. ventricosus* that belong to three top-level GO categories (green: Biological Process, blue: Molecular Function, and yellow: Cellular Component).

**Supplemental Figure S3**

Genomic location of MaSp2AB genes. Dotplot constructed by assembled contig named scaffold_4523. On this scaffold, mainly six repetitive areas are represented, and these areas include *MaSp2A* and *MaSp2B* genes.

**Supplemental Figure S4**

Sequence alignments of *AcSp*. Alignment results with previously reported *AcSp* sequence in *A. ventricosus* (MG021196).

**Supplemental Figure S5**

Sequence alignments of *Flag*. Alignment results with previously reported *Flag* C-terminus region sequence in *A. ventricosus* (EF025541).

**Supplemental Figure S6**

Sequence alignments of *CySp*. Alignment results with previously reported *CySp* sequence in *A. ventricosus* (MF192838).

**Supplemental Figure S7**

Sequence alignments of *MiSp*. Alignment results with previously reported *MiSp* sequence in *A. ventricosus* (JX513956).

**Supplemental Figure S8**

Expression profiles of spidroin genes in each tissue. Gene expression profiles of spidroin genes in the whole body and each abdominal silk gland with three biological replicates per sample. The pictures in each graph are representative images of the samples. Other glands include multiple silk glands other than major ampullate and minor ampullate gland.

**Supplemental Figure S9**

Differentially expressed genes among each gland and body. Pairwise MA plots for expression levels among glands (major ampullate gland, minor ampullate gland, and other spidroin gland) and whole body. Each number represents the total number of DEGs identified based on FDR < 5%.

**Supplemental Figure S10**

Detected peptides in MaSp3 protein sequence. Red box amino acids represent the detected peptides from dragline silk by mass spectrometry analysis.

**Supplemental Figure S11**

Sampling point of starvation test. Over the course of two weeks, spiders were fed at day 1 (Oct 12), day 4 (Oct 15), and day 7 (Oct 18), and the dragline silks were sampled at day 2 (Oct 13; Feeding control 1: for 1 day after feeding), day 5 (Oct 16; Feeding control 2: for 1 day after feeding), and day 14 (Oct 25; Starvation one week: for 1 week after feeding).

**Supplemental Figure S12**

Expression level of CRP-like genes. Expression level calculated as TPM of four CRP-like genes in abdominal spidroin glands (major ampullate, minor ampullate, and other sipdroin gland) and whole body. Each color represents the predicted CRP-like gene. Expression levels were represented as the median among biological replicates.

**Supplemental Figure S13**

Amino acid frequency of SpICE proteins and spidroins. This heatmap shows the calculated amino acid compositions of SpICE proteins (g149799.t1, g149801.t1, g22833.t1, and g160600.t1) and spidroins (MaSp1, 2AB, 3, and MiSpAB) in *A. ventricosus*.

**Supplemental Figure S14**

The figure represents a phylogenetic summary of the known spidroins. The report information is automatically obtained from NCBI Databases. The major ampullate gland silk related proteins (MaSp) are widely found. The gray box means family specific or uncategorized spidroins. This phylogenetic tree is based on Garrison, *et al*., 2016^1^.


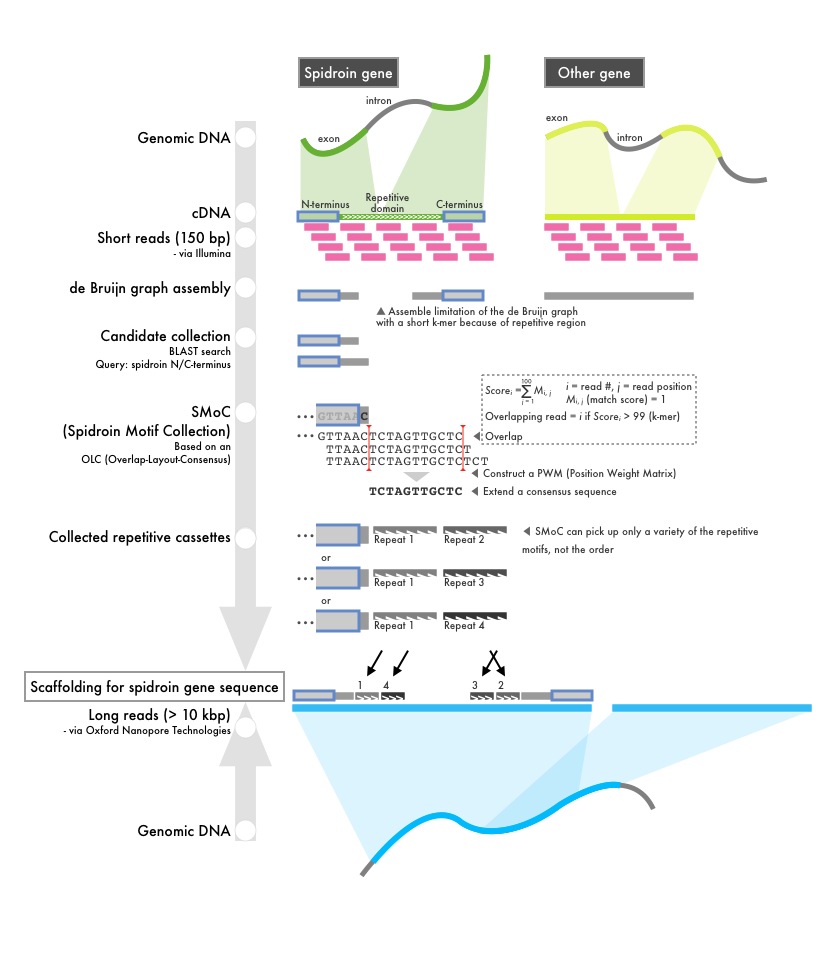


**Supplemental Figure S15**

The conceptual diagram of the spidroin gene curation. The SMoC algorithm first picks up the spidroin gene N/C-terminus candidates (non-repetitive region) with BLAST search from assembled genomic contigs, and repetitive regions from transcriptome assembly. These candidates are used as seed sequences for a screening of the short reads harboring an exact match of extremely large k-mer (approximately 100) up to the 5’-end, and the obtained short reads are aligned to constructs a PWM (Position Weight Matrix) on the 3’-side of the matching k-mer. Using very strict thresholds, seed sequence is extended based on the PWM until there is a split in the graph; i.e., neighboring repeat is not resolvable. By repeating this overlap-based extension algorithm, We can obtain the full length subsets of the repeat units. Finally, these pre-assembled repeat units are mapped onto error-corrected long reads obtained from the direct sequencing of the genomic DNA or RNA.

**Supplemental Table S1**

Summary statistics of MinION reads

| **Direct-DNA** |  | DRR138402 |
| --- | --- | --- |
|  | Read number | 5,545,821 |
|  | Total read length (bp) | 21,314,632,516 |
|  | Longest read length (bp) | 765,336 |
|  | N50 (bp) | 7,386 |
|  | N90 (bp) | 1,631 |
| **Direct-RNA** |  | DRR138400 |
|  | Read number | 156,143 |
|  | Total read length (bp) | 106,714,745 |
|  | Longest read length (bp) | 66,918 |
|  | N50 (bp) | 950 |
|  | N90 (bp) | 356 |

**Supplemental Table S2**

GenomeScope results

| **Property** | **min** | **max** |
| --- | --- | --- |
| Heterozygosity | 2.60% | 2.62% |
| Genome Haploid Length (bp) | 2,150,970,794 | 2,159,369,778 |
| Genome Repeat Length (bp) | 804,143,288 | 807,283,259 |
| Genome Unique Length (bp) | 1,346,827,506 | 1,352,086,519 |
| Model Fit | 94.55% | 99.07% |
| Read Error Rate | 1.37% | 1.37% |

**Supplemental Table S3**

Summary statistics of repeat elements

| **bases masked:** |  | **1869730663** | **bp** | **51.13** | **%** |
| --- | --- | --- | --- | --- | --- |
|  | **Number of elements^*^** | **Length occupied** | | **Percentage of sequence** | |
| **SINEs:** | 55179 | 17271709 | bp | 0.47 | % |
| ALUs | 0 | 0 | bp | 0 |  |
| MIRs | 0 | 0 | bp | 0 |  |
|  |  |  |  |  |  |
| **LINEs:** | 196675 | 68190993 | bp | 1.86 | % |
| LINE1 | 0 | 0 | bp | 0 | % |
| LINE2 | 997 | 832036 | bp | 0.02 | % |
| L3/CR1 | 40660 | 8312765 | bp | 0.23 | % |
|  |  |  |  |  |  |
| **LTR elements:** | 75722 | 42714172 | bp | 1.17 | % |
| ERVL | 0 | 0 | bp | 0 | % |
| ERVL-MaLRs | 0 | 0 | bp | 0 | % |
| ERV_classI | 0 | 0 | bp | 0 | % |
| ERV_classII | 0 | 0 | bp | 0 | % |
|  |  |  |  |  |  |
| **DNA elements:** | 1391644 | 378426369 | bp | 10.35 | % |
| hAT-Charlie | 153632 | 34387212 | bp | 0.94 | % |
| TcMar-Tigger | 87648 | 18178710 | bp | 0.5 | % |
|  |  |  |  |  |  |
| **Unclassified** | 5547427 | 1315241482 | bp | 35.97 | % |
|  |  |  |  |  |  |
| **Total interspersed repeats:** | | 1821844725 | bp | 49.82 | % |
|  |  |  |  |  |  |
|  |  |  |  |  |  |
| **Small RNA:** | 6128 | 1260615 | bp | 0.03 | % |
|  |  |  |  |  |  |
| **Satellites:** | 104322 | 25037518 | bp | 0.68 | % |
| **Simple repeats:** | 456804 | 21283859 | bp | 0.58 | % |
| **Low complexity:** | 105168 | 5075781 | bp | 0.14 | % |
|  |  |  |  |  |  |
| * most repeats fragmented by insertions or deletions have been counted as one element. | | | | |  |

**Supplemental Table S4**

Summary of *Araneus ventricosus* samples

| **Spider sample ID** | **Geographical coordinate** | **Purpose** | **Accession number** |
| --- | --- | --- | --- |
| 5789 | 38.746634, 139.824507 | GemCoded sequencing of genomic DNA | DRR138401 |
|  |  | Direct-DNA sequencing of genomic DNA | DRR138402 |
|  |  | Direct-RNA sequencing of mRNA | DRR138400 |
| 254 | 38.752695,139.742315 | cDNA sequencing of mRNA in whole body | DRR129313 |
| 722 | 32.864505,130.910161 | cDNA sequencing of mRNA in whole body | DRR129314 |
| 778 | 32.914196,130.983727 | cDNA sequencing of mRNA in whole body | DRR129315 |
| 638 | 32.919101,131.073098 | cDNA sequencing of mRNA in whole body | DRR129316 |
| 780 | 32.914196,130.983727 | cDNA sequencing of mRNA in whole body | DRR129317 |
| 4161, 4184, 5761-5790 | 39.881729, 140.051056 | cDNA sequencing of mRNA in major ampullate | DRR138403-5 |
|  |  | cDNA sequencing of mRNA in minor ampullate | DRR138406-8 |
|  |  | cDNA sequencing of mRNA in other spidroin glands | DRR138409-11 |
| **Spider sample ID** | **Geographical coordinate** | **Purpose** | **Data list** |
| 4152,4153,4155,4160,4162,4169,4170,4171,4180,4181,4184 | 39.881729, 140.051056 | Dragline silk for mechacnical property and proteome analysis | table 11 |

**Supplemental Table S5**

Spidroin genes in genomic location

| **Spidroin name** | **ID** | **Scaffold** | **Location** |
| --- | --- | --- | --- |
| MiSpA | g264011.t1 | scaffold_4145 | 39598..53768 |
| MiSpB | g186744.t1 | scaffold_4122 | 7810..27717 |
| MaSp1 | g121555.t1 | scaffold_1468 | 174745..184572 |
| MaSp2A | g171745.t1 | scaffold_4523 | 61303..70712 |
| MaSp2B | g171746.t1 | scaffold_4523 | 92693..110583 |
| MaSp3 | g7867.t1 | scaffold_7272 | 36131..43987 |
| Flag | g100465.t1 | scaffold_6609 | 9400..62610 |
| PySp | g16081.t1 | scaffold_22312 | 9208..19770 |
| CySp | g18336.t1 | scaffold_1782 | 7025..14902 |
| AgSp | g118932.t1 | scaffold_123 | 327264..360638 |
| AcSp | g246635.t1 | scaffold_15691 | 12390..22727 |

**Supplemental Table S6**

Summary of known spidroin genes

| **Organism** | **ID** | **Description** | **Database** |
| --- | --- | --- | --- |
| *Araneus bicentenarius* | ABU20328 | spidroin 2 gene, partial cds. | NCBI |
| *Araneus bicentenarius* | ABU03847 | spidroin gene, partial cds. | NCBI |
| *Araneus diadematus* | MF955809 | Major ampullate Spidroin 2 C-terminal variant 1 | Ayoub et al. 2007 |
| *Araneus diadematus* | MF955810 | Major ampullate Spidroin 2 C-terminal variant 2 | Ayoub et al. 2007 |
| *Araneus diadematus* | MF955811 | Major ampullate Spidroin 2 C-terminal variant 3 | Ayoub et al. 2007 |
| *Araneus diadematus* | MF955691 | Major ampullate Spidroin 3 N-terminal variant 1 | Ayoub et al. 2007 |
| *Araneus diadematus* | ADU47853 | fibroin-1 (ADF-1) mRNA, partial cds. | NCBI |
| *Araneus diadematus* | ADU47854 | fibroin-2 (ADF-2) mRNA, partial cds. | NCBI |
| *Araneus diadematus* | ADU47855 | fibroin-3 (ADF-3) mRNA, partial cds. | NCBI |
| *Araneus diadematus* | ADU47856 | fibroin-4 mRNA, partial cds. | NCBI |
| *Araneus gemmoides* | AY855100 | isolate 1 tubuliform spidroin mRNA, partial cds. | NCBI |
| *Araneus gemmoides* | AY855101 | isolate 2 tubuliform spidroin mRNA, partial cds. | NCBI |
| *Araneus gemmoides* | JF827034 | piriform spidroin gene, partial cds. | NCBI |
| *Araneus ventricosus* | AY177203 | major ampullate gland dragline silk protein-2 (F2) mRNA, partial cds. | NCBI |
| *Araneus ventricosus* | JN857964 | major ampullate spidroin 1 silk protein (MaSp1) mRNA, partial cds. | NCBI |
| *Araneus ventricosus* | HQ008714.1 | aciniform spidroin 1 (AcSp1) | NCBI |
| *Araneus ventricosus* | JX513954 | clone AvMiSp1 minor ampullate spidroin mRNA, partial cds. | NCBI |
| *Araneus ventricosus* | JX513955 | clone AvMiSp2 minor ampullate spidroin mRNA, partial cds. | NCBI |
| *Araneus ventricosus* | JX513956 | clone F29-0811 minor ampullate spidroin gene, complete cds. | NCBI |
| *Araneus ventricosus* | EF025541 | flagelliform fibroin mRNA, complete cds. | NCBI |
| *Araneus ventricosus* | AY587193 | flagelliform silk protein-1 (Ff1) mRNA, partial cds. | NCBI |
| *Araneus ventricosus* | AY940091 | major ampullate gland dragline silk protein (F1s) mRNA, partial cds. | NCBI |
| *Araneus ventricosus* | AY945306 | major ampullate gland dragline silk protein (FU5) mRNA, partial cds. | NCBI |
| *Araneus ventricosus* | EF025086 | major ampullate gland dragline silk protein (Sds) gene, partial cds. | NCBI |
| *Araneus ventricosus* | DQ536503 | major ampullate gland dragline silk protein 2 (GF2) gene, partial cds. | NCBI |
| *Araneus ventricosus* | AY174110 | major ampullate gland dragline silk protein-1 (F1) mRNA, partial cds. | NCBI |
| *Araneus ventricosus* | This study | MaSp1 | This study |
| *Araneus ventricosus* | This study | MaSp2A | This study |
| *Araneus ventricosus* | This study | MaSp2B | This study |
| *Araneus ventricosus* | This study | MaSp3 | This study |
| *Araneus ventricosus* | This study | MiSpA | This study |
| *Araneus ventricosus* | This study | MiSpB | This study |
| *Araneus ventricosus* | This study | AcSp | This study |
| *Araneus ventricosus* | This study | PySp | This study |
| *Araneus ventricosus* | This study | CySp | This study |
| *Araneus ventricosus* | This study | AgSp | This study |
| *Araneus ventricosus* | This study | Flag | This study |
| *Argiope amoena* | AY365015 | clone 12 major ampullate spidroin-2 (MaSp2) mRNA, partial cds. | NCBI |
| *Argiope amoena* | AY365016 | clone 2 major ampullate spidroin-2 (MaSp2) mRNA, partial cds. | NCBI |
| *Argiope amoena* | AY365017 | clone 25 major ampullate spidroin-2 (MaSp2) mRNA, partial cds. | NCBI |
| *Argiope amoena* | AY365018 | clone 3 major ampullate spidroin-2 (MaSp2) mRNA, partial cds. | NCBI |
| *Argiope amoena* | AY365019 | clone 5 major ampullate spidroin-2 (MaSp2) mRNA, partial cds. | NCBI |
| *Argiope amoena* | AY365021 | clone 6 major ampullate spidroin-2 (MaSp2) mRNA, partial cds. | NCBI |
| *Argiope amoena* | AY365020 | clone 7 major ampullate spidroin-2 (MaSp2) mRNA, partial cds. | NCBI |
| *Argiope amoena* | AY263390 | major ampullate spidroin-1 (MaSp1) mRNA, complete cds. | NCBI |
| *Argiope amoena* | HQ008715 | aciniform spidroin 1 (AcSp1) mRNA, partial cds. | NCBI |
| *Argiope amoena* | AY453691 | silk gland protein 1 mRNA, complete cds. | NCBI |
| *Argiope amoena* | JQ291306 | tubuliform spidroin 1 (TuSp1) mRNA, partial cds. | NCBI |
| *Argiope argentata* | MF955690 | Major ampullate Spidroin 3 C-terminal variant 1 | Ayoub et al. 2007 |
| *Argiope argentata* | MF955785 | Major ampullate Spidroin 3 N-terminal variant 1 | Ayoub et al. 2007 |
| *Argiope argentata* | KJ206597.1 | aciniform spidrion 1 | NCBI |
| *Argiope argentata* | KJ206600.1 | aciniform spidroin 1 C-terminal region variant 1 | NCBI |
| *Argiope argentata* | KJ206601.1 | aciniform spidroin 1 C-terminal region variant 2 | NCBI |
| *Argiope argentata* | KJ206602.1 | aciniform spidroin 1 C-terminal region variant 3 | NCBI |
| *Argiope argentata* | KJ206603.1 | aciniform spidroin 1 C-terminal region variant 4 | NCBI |
| *Argiope argentata* | KJ206604.1 | aciniform spidroin 1 C-terminal region variant 5 | NCBI |
| *Argiope argentata* | KJ206605.1 | aciniform spidroin 1 C-terminal region variant 6 | NCBI |
| *Argiope argentata* | KJ206606.1 | aciniform spidroin 1 C-terminal region variant 7 | NCBI |
| *Argiope argentata* | KJ206570.1 | aciniform spidroin 1 N-terminal region variant 1 | NCBI |
| *Argiope argentata* | KJ206571.1 | aciniform spidroin 1 N-terminal region variant 2 | NCBI |
| *Argiope argentata* | KJ206572.1 | aciniform spidroin 1 N-terminal region variant 3 | NCBI |
| *Argiope argentata* | KJ206573.1 | aciniform spidroin 1 N-terminal region variant 4 | NCBI |
| *Argiope argentata* | KJ206574.1 | aciniform spidroin 1 N-terminal region variant 5 | NCBI |
| *Argiope argentata* | KJ206575.1 | aciniform spidroin 1 N-terminal region variant 6 | NCBI |
| *Argiope argentata* | KJ206576.1 | aciniform spidroin 1 N-terminal region variant 7 | NCBI |
| *Argiope argentata* | KJ206620.1 | clone 169K24 aciniform spidroin 1 | NCBI |
| *Argiope argentata* | JQ713003 | clone AAMIN10 minor ampullate spidroin (MiSp) mRNA, partial cds. | NCBI |
| *Argiope argentata* | JQ713004 | clone AAMIN11 minor ampullate spidroin (MiSp) mRNA, partial cds. | NCBI |
| *Argiope argentata* | HM752577 | clone AT29F tubuliform spidroin 1 mRNA, partial cds. | NCBI |
| *Argiope argentata* | AY953084 | isolate AarC tubuliform spidroin 1 gene, partial cds. | NCBI |
| *Argiope argentata* | AY953091 | isolate AarR tubuliform spidroin 1 gene, partial cds. | NCBI |
| *Argiope argentata* | AY953071 | isolate AT69 tubuliform spidroin 1 mRNA, partial cds. | NCBI |
| *Argiope aurantia* | AF350263.1 | major ampullate spidroin 2 (MaSp2) | NCBI |
| *Argiope aurantia* | KJ206607.1 | aciniform spidroin 1 C-terminal region variant 1 | NCBI |
| *Argiope aurantia* | KJ206608.1 | aciniform spidroin 1 C-terminal region variant 2 | NCBI |
| *Argiope aurantia* | KJ206577.1 | aciniform spidroin 1 N-terminal region variant 1 | NCBI |
| *Argiope aurantia* | KJ206578.1 | aciniform spidroin 1 N-terminal region variant 2 | NCBI |
| *Argiope aurantia* | KJ206579.1 | aciniform spidroin 1 N-terminal region variant 3 | NCBI |
| *Argiope aurantia* | KJ206580.1 | aciniform spidroin 1 N-terminal region variant 4 | NCBI |
| *Argiope aurantia* | KJ206581.1 | aciniform spidroin 1 N-terminal region variant 5 | NCBI |
| *Argiope aurantia* | KJ206582.1 | aciniform spidroin 1 N-terminal region variant 6 | NCBI |
| *Argiope aurantia* | AY855098 | isolate 1 tubuliform spidroin mRNA, partial cds. | NCBI |
| *Argiope aurantia* | AY855099 | isolate 2 tubuliform spidroin mRNA, partial cds. | NCBI |
| *Argiope aurantia* | AY953081 | isolate AauC tubuliform spidroin 1 gene, partial cds. | NCBI |
| *Argiope aurantia* | AY953092 | isolate AauR tubuliform spidroin 1 gene, partial cds. | NCBI |
| *Argiope aurantia* | AF350262 | major ampullate spidroin 1 (MaSp1) gene, partial cds. | NCBI |
| *Argiope bruennichi* | JX112871 | major ampullate silk protein 1 (MASP1) mRNA, partial cds. | NCBI |
| *Argiope bruennichi* | JX112872 | major ampullate silk protein 2 (MASP2) mRNA, complete cds. | NCBI |
| *Argiope bruennichi* | JX202781 | major ampullate silk protein 2 partial | NCBI |
| *Argiope bruennichi* | AB242144 | CySp1_C | NCBI |
| *Argiope bruennichi* | AB242145 | CySp2_C | NCBI |
| *Argiope trifasciata* | AF350266 | major ampullate spidroin 1 (MaSp1) mRNA, partial cds. | NCBI |
| *Argiope trifasciata* | DQ059137.1 | major ampullate spidroin 2 | NCBI |
| *Argiope trifasciata* | AF350267 | major ampullate spidroin 2 (MaSp2) mRNA, partial cds. | NCBI |
| *Argiope trifasciata* | DQ059136S2 | major ampullate spidroin 2 | NCBI |
| *Argiope trifasciata* | DQ059136S1 | major ampullate spidroin 2 | NCBI |
| *Argiope trifasciata* | AH015065.1 | major ampullate spidroin 2 s | NCBI |
| *Argiope trifasciata* | AH015065.1 | major ampullate spidroin 2 s | NCBI |
| *Argiope trifasciata* | KJ206599.1 | aciniform spidrion 1 | NCBI |
| *Argiope trifasciata* | KJ206609.1 | aciniform spidroin 1 C-terminal region variant 1 | NCBI |
| *Argiope trifasciata* | KJ206618.1 | aciniform spidroin 1 C-terminal region variant 10 | NCBI |
| *Argiope trifasciata* | KJ206619.1 | aciniform spidroin 1 C-terminal region variant 11 | NCBI |
| *Argiope trifasciata* | KJ206610.1 | aciniform spidroin 1 C-terminal region variant 2 | NCBI |
| *Argiope trifasciata* | KJ206611.1 | aciniform spidroin 1 C-terminal region variant 3 | NCBI |
| *Argiope trifasciata* | KJ206612.1 | aciniform spidroin 1 C-terminal region variant 4 | NCBI |
| *Argiope trifasciata* | KJ206613.1 | aciniform spidroin 1 C-terminal region variant 5 | NCBI |
| *Argiope trifasciata* | KJ206614.1 | aciniform spidroin 1 C-terminal region variant 6 | NCBI |
| *Argiope trifasciata* | KJ206615.1 | aciniform spidroin 1 C-terminal region variant 7 | NCBI |
| *Argiope trifasciata* | KJ206616.1 | aciniform spidroin 1 C-terminal region variant 8 | NCBI |
| *Argiope trifasciata* | KJ206617.1 | aciniform spidroin 1 C-terminal region variant 9 | NCBI |
| *Argiope trifasciata* | AY426339 | aciniform spidroin 1 mRNA, partial cds. | NCBI |
| *Argiope trifasciata* | KJ206583.1 | aciniform spidroin 1 N-terminal region variant 1 | NCBI |
| *Argiope trifasciata* | KJ206592.1 | aciniform spidroin 1 N-terminal region variant 10 | NCBI |
| *Argiope trifasciata* | KJ206593.1 | aciniform spidroin 1 N-terminal region variant 11 | NCBI |
| *Argiope trifasciata* | KJ206594.1 | aciniform spidroin 1 N-terminal region variant 12 | NCBI |
| *Argiope trifasciata* | KJ206595.1 | aciniform spidroin 1 N-terminal region variant 13 | NCBI |
| *Argiope trifasciata* | KJ206596.1 | aciniform spidroin 1 N-terminal region variant 14 | NCBI |
| *Argiope trifasciata* | KJ206584.1 | aciniform spidroin 1 N-terminal region variant 2 | NCBI |
| *Argiope trifasciata* | KJ206585.1 | aciniform spidroin 1 N-terminal region variant 3 | NCBI |
| *Argiope trifasciata* | KJ206586.1 | aciniform spidroin 1 N-terminal region variant 4 | NCBI |
| *Argiope trifasciata* | KJ206587.1 | aciniform spidroin 1 N-terminal region variant 5 | NCBI |
| *Argiope trifasciata* | KJ206588.1 | aciniform spidroin 1 N-terminal region variant 6 | NCBI |
| *Argiope trifasciata* | KJ206589.1 | aciniform spidroin 1 N-terminal region variant 7 | NCBI |
| *Argiope trifasciata* | KJ206590.1 | aciniform spidroin 1 N-terminal region variant 8 | NCBI |
| *Argiope trifasciata* | KJ206591.1 | aciniform spidroin 1 N-terminal region variant 9 | NCBI |
| *Argiope trifasciata* | AF350264.1 | flagelliform silk protein (Flag) | NCBI |
| *Argiope trifasciata* | AF350265 | flagelliform silk protein (Flag) mRNA, partial cds. | NCBI |
| *Argiope trifasciata* | AF350268 | major ampullate spidroin 2-like protein gene, partial cds. | NCBI |
| *Argiope trifasciata* | GQ980328 | piriform spidroin mRNA, partial cds. | NCBI |
| *Cyrtophora moluccensis* | KF032719 | major ampullate spidroin 1 short (MaSp1s) gene, complete cds. | NCBI |
| *Cyrtophora moluccensis* | AY666061 | clone CM05-PS09 dragline silk spidroin 1 gene, partial cds. | NCBI |
| *Cyrtophora moluccensis* | AY666062 | clone CM05-PS11 dragline silk spidroin 1 gene, partial cds. | NCBI |
| *Cyrtophora moluccensis* | AY666060 | clone CM06-SSA04 dragline silk spidroin 1 gene, partial cds. | NCBI |
| *Cyrtophora moluccensis* | AY666063 | clone CM07-SS01 dragline silk spidroin 1 gene, partial cds. | NCBI |
| *Cyrtophora moluccensis* | AY666078 | clone CM091-RTPS02 dragline silk spidroin 1 mRNA, partial cds. | NCBI |
| *Cyrtophora moluccensis* | AY666079 | clone CM091-RTPS06 dragline silk spidroin 1 mRNA, partial cds. | NCBI |
| *Cyrtophora moluccensis* | AY666080 | clone CM091-RTPS07 dragline silk spidroin 1 mRNA, partial cds. | NCBI |
| *Cyrtophora moluccensis* | AY666081 | clone CM092-RTPS01 dragline silk spidroin 1 mRNA, partial cds. | NCBI |
| *Cyrtophora moluccensis* | AY953083 | isolate CmolC tubuliform spidroin 1 gene, partial cds. | NCBI |
| *Gasteracantha cancriformis* | AF350272 | major ampullate spidroin 2 (MaSp2) gene, partial cds. | NCBI |
| *Gea heptagon* | AY953082 | isolate GhepC tubuliform spidroin 1 gene, partial cds. | NCBI |
| *Gea heptagon* | AY953093 | isolate GhepR tubuliform spidroin 1 gene, partial cds. | NCBI |
| *Latrodectus geometricus* | JX978182 | clone LgSD7 aciniform spidroin 1 (AcSp1) gene, partial cds. | NCBI |
| *Latrodectus geometricus* | JX978181 | clone LgSD7-C aciniform spidroin 1 (AcSp1) gene, partial cds. | NCBI |
| *Latrodectus geometricus* | JX978180.1 | clone LgSD7-N aciniform spidroin 1 (AcSp1) | NCBI |
| *Latrodectus geometricus* | AY953079 | isolate LgeoC tubuliform spidroin 1 gene, partial cds. | NCBI |
| *Latrodectus geometricus* | AY953089 | isolate LgeoR tubuliform spidroin 1 gene, partial cds. | NCBI |
| *Latrodectus geometricus* | AF350273.1 | major ampullate spidroin 1 (MaSp1) | NCBI |
| *Latrodectus geometricus* | DQ059134.1 | major ampullate spidroin 1-like | NCBI |
| *Latrodectus geometricus* | DQ059133S1 | major ampullate spidroin 1-like gene, partial cds. | NCBI |
| *Latrodectus geometricus* | AH015064.1 | major ampullate spidroin 1-like s | NCBI |
| *Latrodectus geometricus* | AH015064.1 | major ampullate spidroin 1-like s | NCBI |
| *Latrodectus geometricus* | AF350275.1 | major ampullate spidroin 2 (MaSp2) | NCBI |
| *Latrodectus geometricus* | EU177657 | major ampullate spidroin 2 (MaSp2) gene, partial cds. | NCBI |
| *Latrodectus geometricus* | AF350274 | major ampullate spidroin 2 (MaSp2) mRNA, partial cds. | NCBI |
| *Latrodectus geometricus* | AY685201 | major ampullate spidroin-like protein mRNA, partial cds. | NCBI |
| *Latrodectus geometricus* | EU177660.1 | voucher LgSD7 major ampullate spidroin 1 locus 3 (MaSp1) | NCBI |
| *Latrodectus geometricus* | EU177666 | voucher LgSD7 major ampullate spidroin 1 variant 1 locus 1 (MaSp1) gene, partial cds. | NCBI |
| *Latrodectus geometricus* | EU177668 | voucher LgSD7 major ampullate spidroin 1 variant 1 locus 2 (MaSp1) gene, partial cds. | NCBI |
| *Latrodectus geometricus* | EU177667 | voucher LgSD7 major ampullate spidroin 1 variant 2 locus 1 (MaSp1) gene, partial cds. | NCBI |
| *Latrodectus geometricus* | EU177669 | voucher LgSD7 major ampullate spidroin 1 variant 2 locus 2 (MaSp1) gene, partial cds. | NCBI |
| *Latrodectus geometricus* | DQ059133S2 | MaSp1-like_C | NCBI |
| *Latrodectus hasselti* | AY953080 | isolate LhasC tubuliform spidroin 1 gene, partial cds. | NCBI |
| *Latrodectus hasselti* | AY953088 | isolate LhasR tubuliform spidroin 1 gene, partial cds. | NCBI |
| *Latrodectus hesperus* | KX584003 | 133N14 minor apmulate spidroin | NCBI |
| *Latrodectus hesperus* | EU025854 | aciniform spidroin 1-like mRNA, partial cds. | NCBI |
| *Latrodectus hesperus* | EF153411.1 | aqueous glue droplet peptide (SCP-1) | NCBI |
| *Latrodectus hesperus* | EF153412.1 | aqueous glue droplet peptide (SCP-2) | NCBI |
| *Latrodectus hesperus* | EU177649 | clone 110A1 major ampullate spidroin 1 locus 1 (MaSp1) gene, partial cds. | NCBI |
| *Latrodectus hesperus* | EU177649 | clone 110A1 major ampullate spidroin 1 locus 1 (MaSp1) gene, partial cds. | NCBI |
| *Latrodectus hesperus* | EU177648.1 | clone 113P20 major ampullate spidroin 1 locus 3 (MaSp1) | NCBI |
| *Latrodectus hesperus* | EU177648.1 | clone 113P20 major ampullate spidroin 1 locus 3 (MaSp1) | NCBI |
| *Latrodectus hesperus* | EU177655 | clone 11E24 major ampullate spidroin 1 locus 1 (MaSp1) gene, partial cds. | NCBI |
| *Latrodectus hesperus* | EU177655 | clone 11E24 major ampullate spidroin 1 locus 1 (MaSp1) gene, partial cds. | NCBI |
| *Latrodectus hesperus* | EU177654 | clone 14C24 major ampullate spidroin 1 locus 1 (MaSp1) gene, partial cds. | NCBI |
| *Latrodectus hesperus* | EU177654 | clone 14C24 major ampullate spidroin 1 locus 1 (MaSp1) gene, partial cds. | NCBI |
| *Latrodectus hesperus* | EU177653 | clone 14I6 major ampullate spidroin 1 locus 2 (MaSp1) gene, partial cds. | NCBI |
| *Latrodectus hesperus* | EU177653 | clone 14I6 major ampullate spidroin 1 locus 2 (MaSp1) gene, partial cds. | NCBI |
| *Latrodectus hesperus* | JX262192.1 | clone 2525 aggregate gland silk factor 2 | NCBI |
| *Latrodectus hesperus* | JX978171 | clone 28K13 aciniform spidroin 1 (AcSp1) gene, complete cds. | NCBI |
| *Latrodectus hesperus* | JX978172 | clone 315N21 aciniform spidroin 1 (AcSp1) gene, partial cds. | NCBI |
| *Latrodectus hesperus* | JX978173 | clone 333E15 aciniform spidroin 1 (AcSp1) gene, partial cds. | NCBI |
| *Latrodectus hesperus* | JX978174.1 | clone 368N11 aciniform spidroin 1 (AcSp1) | NCBI |
| *Latrodectus hesperus* | EU177652 | clone 38E21 major ampullate spidroin 2 (MaSp2) gene, partial cds. | NCBI |
| *Latrodectus hesperus* | EU177652 | clone 38E21 major ampullate spidroin 2 (MaSp2) gene, partial cds. | NCBI |
| *Latrodectus hesperus* | JX262189.1 | clone 44 aggregate spider glue 2-like protein | NCBI |
| *Latrodectus hesperus* | JX262195.1 | clone 549 aggregate gland silk factor 1 | NCBI |
| *Latrodectus hesperus* | EU177651 | clone 63L5 major ampullate spidroin 1 locus 2 (MaSp1) gene, partial cds. | NCBI |
| *Latrodectus hesperus* | EU177651 | clone 63L5 major ampullate spidroin 1 locus 2 (MaSp1) gene, partial cds. | NCBI |
| *Latrodectus hesperus* | JX978175 | clone 79D23 aciniform spidroin 1 (AcSp1) gene, partial cds. | NCBI |
| *Latrodectus hesperus* | EU177650 | clone 89K13 major ampullate spidroin 1 locus 3 (MaSp1) gene, partial cds. | NCBI |
| *Latrodectus hesperus* | EU177650 | clone 89K13 major ampullate spidroin 1 locus 3 (MaSp1) gene, partial cds. | NCBI |
| *Latrodectus hesperus* | HQ005885.1 | clone CV115 egg case fibroin | NCBI |
| *Latrodectus hesperus* | HQ005889.1 | clone CV119 tubuliform spidroin 1 | NCBI |
| *Latrodectus hesperus* | HQ005791.1 | clone CV21 pyriform spidroin 1 | NCBI |
| *Latrodectus hesperus* | HQ005804.1 | clone CV34 major ampullate spidroin 1 | NCBI |
| *Latrodectus hesperus* | HQ005853.1 | clone CV83 egg case silk protein 2 | NCBI |
| *Latrodectus hesperus* | HM752571 | clone LM39 minor ampullate spidroin mRNA, partial cds. | NCBI |
| *Latrodectus hesperus* | HM752570 | clone LM61 minor ampullate spidroin mRNA, partial cds. | NCBI |
| *Latrodectus hesperus* | JX978179 | clone marbled aciniform spidroin 1 (AcSp1) gene, partial cds. | NCBI |
| *Latrodectus hesperus* | JX978176 | clone UCR_Lh10 aciniform spidroin 1 (AcSp1) gene, partial cds. | NCBI |
| *Latrodectus hesperus* | JX978177 | clone UCR_Lh11 aciniform spidroin 1 (AcSp1) gene, partial cds. | NCBI |
| *Latrodectus hesperus* | JX978178 | clone UCR_Lh12 aciniform spidroin 1 (AcSp1) gene, partial cds. | NCBI |
| *Latrodectus hesperus* | MF955692 | Major ampullate Spidroin 3 C-terminal variant 1 | Ayoub et al. 2007 |
| *Latrodectus hesperus* | MF955786 | Major ampullate Spidroin 3 N-terminal variant 1 | Ayoub et al. 2007 |
| *Latrodectus hesperus* | DQ109035 | egg case fibroin mRNA, partial cds. | NCBI |
| *Latrodectus hesperus* | DQ341220 | egg case silk protein 2 (ECP-2) mRNA, complete cds. | NCBI |
| *Latrodectus hesperus* | AY994149 | egg case silk protein-1 (ECP-1) mRNA, complete cds. | NCBI |
| *Latrodectus hesperus* | AY953076.1 | isolate LhesmarC tubuliform spidroin 1 | NCBI |
| *Latrodectus hesperus* | AY953086 | isolate LhesmarR tubuliform spidroin 1 gene, partial cds. | NCBI |
| *Latrodectus hesperus* | AY953070.1 | isolate LT103 tubuliform spidroin 1 | NCBI |
| *Latrodectus hesperus* | AY953074 | isolate LT51 major ampullate spidroin 1 mRNA, partial cds. | NCBI |
| *Latrodectus hesperus* | AY953075.1 | isolate LT57 major ampullate spidroin 2 | NCBI |
| *Latrodectus hesperus* | EU177658 | isolate marbled major ampullate spidroin 1 locus 1 (MaSp1) gene, partial cds. | NCBI |
| *Latrodectus hesperus* | EU177665.1 | isolate marbled major ampullate spidroin 1 locus 2 (MaSp1) | NCBI |
| *Latrodectus hesperus* | DQ409057.1 | major ampullate spidroin 1 (MaSp1) | NCBI |
| *Latrodectus hesperus* | EF595247 | major ampullate spidroin 1 (MaSp1) mRNA, partial cds. | NCBI |
| *Latrodectus hesperus* | DQ379381 | major ampullate spidroin 1 gene, partial cds. | NCBI |
| *Latrodectus hesperus* | EU177656 | major ampullate spidroin 2 (MaSp2) gene, partial cds. | NCBI |
| *Latrodectus hesperus* | DQ409058 | major ampullate spidroin 2 (MaSp2) mRNA, partial cds. | NCBI |
| *Latrodectus hesperus* | EF595248 | major ampullate spidroin 2 (MaSp2) mRNA, partial cds. | NCBI |
| *Latrodectus hesperus* | DQ379382 | major ampullate spidroin 2 gene, partial cds. | NCBI |
| *Latrodectus hesperus* | EU394445 | minor ampullate spidroin 1-like protein mRNA, partial cds. | NCBI |
| *Latrodectus hesperus* | FJ973621 | pyriform spidroin 1 mRNA, partial cds. | NCBI |
| *Latrodectus hesperus* | EU177661 | voucher UCR_Lh10 major ampullate spidroin 1 locus 1 (MaSp1) gene, partial cds. | NCBI |
| *Latrodectus hesperus* | EU177662 | voucher UCR_Lh10 major ampullate spidroin 1 locus 2 (MaSp1) gene, partial cds. | NCBI |
| *Latrodectus hesperus* | EU177659 | voucher UCR_Lh10 major ampullate spidroin 1 locus 3 (MaSp1) gene, partial cds. | NCBI |
| *Latrodectus hesperus* | EU177663 | voucher UCR_Lh11 major ampullate spidroin 1 locus 1 (MaSp1) gene, partial cds. | NCBI |
| *Latrodectus hesperus* | EU177664 | voucher UCR_Lh11 major ampullate spidroin 1 locus 2 (MaSp1) gene, partial cds. | NCBI |
| *Latrodectus hesperus* | DQ379383 | tubuliform spidroin 1 | NCBI |
| *Latrodectus hesperus* | EF595245 | major ampullate spidroin 2 | NCBI |
| *Latrodectus hesperus* | EF595246 | major ampullate spidroin 1 | NCBI |
| *Latrodectus mactans* | AY953077 | isolate LatDRC tubuliform spidroin 1 gene, partial cds. | NCBI |
| *Latrodectus mactans* | AY953085 | isolate LatDRR tubuliform spidroin 1 gene, partial cds. | NCBI |
| *Latrodectus mactans* | HM752779 | major ampullate spidroin 1 gene, partial cds. | NCBI |
| *Latrodectus tredecimguttatus* | AY953078 | isolate Ltre2C tubuliform spidroin 1 gene, partial cds. | NCBI |
| *Latrodectus tredecimguttatus* | AY953087 | isolate LtreR tubuliform spidroin 1 gene, partial cds. | NCBI |
| *Metepeira grandiosa* | HM752569 | clone MF15 minor ampullate spidroin mRNA, partial cds. | NCBI |
| *Metepeira grandiosa* | HM752575 | clone MF6F minor ampullate spidroin mRNA, partial cds. | NCBI |
| *Metepeira grandiosa* | HM752575 | clone MF6F minor ampullate spidroin mRNA, partial cds. | NCBI |
| *Nephila antipodiana* | DQ338461 | clone D938 major ampullate fibroin 1 mRNA, partial cds. | NCBI |
| *Nephila antipodiana* | DQ338462 | clone 145 minor ampullate fibroin 1 mRNA, partial cds. | NCBI |
| *Nephila antipodiana* | EU730637 | clone TuNRP1 eggcase silk protein mRNA, partial cds. | NCBI |
| *Nephila clavata* | AB218973 | CySp1 for cylindrical silk protein 1 3' region | NCBI |
| *Nephila clavata* | AB218974 | CySp1 for cylindrical silk protein 1 5' region | NCBI |
| *Nephila clavata* | AF441245 | dragline silk protein spidroin 2 mRNA, partial cds. | NCBI |
| *Nephila clavipes* | AY654288 | clone var1 major ampullate spidroin 1 gene, partial cds. | NCBI |
| *Nephila clavipes* | AY654293 | clone var1 major ampullate spidroin 2 gene, partial cds. | NCBI |
| *Nephila clavipes* | AY654289 | clone var2 major ampullate spidroin 1 gene, partial cds. | NCBI |
| *Nephila clavipes* | AY654294 | clone var2 major ampullate spidroin 2 gene, partial cds. | NCBI |
| *Nephila clavipes* | AY654290 | clone var3 major ampullate spidroin 1 gene, partial cds. | NCBI |
| *Nephila clavipes* | AY654295 | clone var3 major ampullate spidroin 2 gene, partial cds. | NCBI |
| *Nephila clavipes* | AY654291 | clone var4 major ampullate spidroin 1 gene, partial cds. | NCBI |
| *Nephila clavipes* | AY654296 | clone var4 major ampullate spidroin 2 gene, partial cds. | NCBI |
| *Nephila clavipes* | AY654292 | clone var5 major ampullate spidroin 1 gene, partial cds. | NCBI |
| *Nephila clavipes* | EU617338 | clone var6 major ampullate spidroin 1 mRNA, partial cds. | NCBI |
| *Nephila clavipes* | AY654297.1 | clone var5 major ampullate spidroin 2 | NCBI |
| *Nephila clavipes* | Babb et al. 2017 | MaSp-g | Babb et al. 2017 |
| *Nephila clavipes* | Babb et al. 2017 | MaSp-h | Babb et al. 2017 |
| *Nephila clavipes* | Babb et al. 2017 | MaSp-b | Babb et al. 2017 |
| *Nephila clavipes* | Babb et al. 2017 | MaSp-d | Babb et al. 2017 |
| *Nephila clavipes* | Babb et al. 2017 | MaSp-f-1 | Babb et al. 2017 |
| *Nephila clavipes* | Babb et al. 2017 | MaSp-a | Babb et al. 2017 |
| *Nephila clavipes* | Babb et al. 2017 | MaSp-c | Babb et al. 2017 |
| *Nephila clavipes* | EU599238 | major ampullate spidroin 1A precursor (MaSp1A) gene, partial cds. | NCBI |
| *Nephila clavipes* | EU599239 | major ampullate spidroin 1B precursor (MaSp1B) gene, partial cds. | NCBI |
| *Nephila clavipes* | EU599240 | major ampullate spidroin 2 precursor (MaSp2) gene, partial cds. | NCBI |
| *Nephila clavipes* | EU780014.1 | aggregate spider glue 1 | NCBI |
| *Nephila clavipes* | EU780015.1 | aggregate spider glue 2 | NCBI |
| *Nephila clavipes* | M37137.2 | dragline silk fibroin | NCBI |
| *Nephila clavipes* | NEPDSF | dragline silk fibroin mRNA, partial cds. | NCBI |
| *Nephila clavipes* | AY061814 | dragline silk protein gene, partial cds. | NCBI |
| *Nephila clavipes* | NCU37520 | dragline silk protein spidroin 1 gene, partial cds. | NCBI |
| *Nephila clavipes* | AF027972 | flagelliform silk protein (Flag) | NCBI |
| *Nephila clavipes* | AF027973.1 | flagelliform silk protein (Flag) | NCBI |
| *Nephila clavipes* | AF218622.1 | flagelliform silk protein (Flag) downstream | NCBI |
| *Nephila clavipes* | AF218621.1 | flagelliform silk protein (Flag) upstream | NCBI |
| *Nephila clavipes* | AH009146.1 | flagelliform silk protein (Flag) and flagelliform silk protein (Flag) | NCBI |
| *Nephila clavipes* | AH009146.1 | flagelliform silk protein (Flag) and flagelliform silk protein (Flag) | NCBI |
| *Nephila clavipes* | EU599241 | major ampullate spidroin 1A precursor (MaSp1A) mRNA, partial cds. | NCBI |
| *Nephila clavipes* | EU599242.1 | major ampullate spidroin 1B precursor (MaSp1B) | NCBI |
| *Nephila clavipes* | EU599243 | major ampullate spidroin 2 precursor (MaSp2) mRNA, partial cds. | NCBI |
| *Nephila clavipes* | AF027735 | minor ampullate silk protein MiSp1 mRNA, partial cds. | NCBI |
| *Nephila clavipes* | AF027737 | minor ampullate silk protein MiSp2 mRNA, partial cds. | NCBI |
| *Nephila clavipes* | AF027736 | minor ampullate silk protein mRNA, partial cds. | NCBI |
| *Nephila clavipes* | GQ980330 | piriform spidroin gene, partial cds. | NCBI |
| *Nephila clavipes* | GQ980329 | piriform spidroin mRNA, partial cds. | NCBI |
| *Nephila clavipes* | HM020705 | pyriform spidroin 2 (PySp2) mRNA, partial cds. | NCBI |
| *Nephila clavipes* | NCU20329 | spidroin 1 mRNA, partial cds. | NCBI |
| *Nephila clavipes* | NCU03848 | spidroin gene, partial cds. | NCBI |
| *Nephila clavipes* | AY855102 | tubuliform spidroin mRNA, partial cds. | NCBI |
| *Nephila clavipes* | Babb et al. 2017 | Sp-1339 | Babb et al. 2017 |
| *Nephila clavipes* | Babb et al. 2017 | AgSp-d | Babb et al. 2017 |
| *Nephila clavipes* | Babb et al. 2017 | MiSp-d | Babb et al. 2017 |
| *Nephila clavipes* | Babb et al. 2017 | AcSp | Babb et al. 2017 |
| *Nephila clavipes* | Babb et al. 2017 | PiSp | Babb et al. 2017 |
| *Nephila clavipes* | Babb et al. 2017 | MiSp-c | Babb et al. 2017 |
| *Nephila clavipes* | Babb et al. 2017 | Sp-8175 | Babb et al. 2017 |
| *Nephila clavipes* | Babb et al. 2017 | MaSp-f-2 | Babb et al. 2017 |
| *Nephila clavipes* | Babb et al. 2017 | CySp | Babb et al. 2017 |
| *Nephila clavipes* | Babb et al. 2017 | AgSp-c | Babb et al. 2017 |
| *Nephila clavipes* | Babb et al. 2017 | MiSp-a | Babb et al. 2017 |
| *Nephila clavipes* | Babb et al. 2017 | FLAG-b | Babb et al. 2017 |
| *Nephila clavipes* | Babb et al. 2017 | AgSp-a | Babb et al. 2017 |
| *Nephila clavipes* | Babb et al. 2017 | Sp-907 | Babb et al. 2017 |
| *Nephila clavipes* | Babb et al. 2017 | MaSp-e | Babb et al. 2017 |
| *Nephila clavipes* | Babb et al. 2017 | MiSp-b | Babb et al. 2017 |
| *Nephila clavipes* | Babb et al. 2017 | AgSp-b | Babb et al. 2017 |
| *Nephila clavipes* | Babb et al. 2017 | Sp-14910-A | Babb et al. 2017 |
| *Nephila clavipes* | Babb et al. 2017 | FLAG-a | Babb et al. 2017 |
| *Nephila clavipes* | Babb et al. 2017 | Sp-14910-B | Babb et al. 2017 |
| *Nephila clavipes* | Babb et al. 2017 | Sp-5803 | Babb et al. 2017 |
| *Nephila clavipes* | Babb et al. 2017 | Sp-74867 | Babb et al. 2017 |
| *Nephila clavipes* | M92913.1 | dragline silk fibroin | NCBI |
| *Nephila clavipes* | NEPFIBPR | dragline silk fibroin | NCBI |
| *Nephila inaurata* | AF350277 | major ampullate spidroin 1 (MaSp1) gene, partial cds. | NCBI |
| *Nephila inaurata* | AH009147.2 | flagelliform silk protein (Flag) | NCBI |
| *Nephila inaurata* | AH009147.2 | flagelliform silk protein (Flag) | NCBI |
| *Nephila inaurata* | AF218623.1 | flagelliform silk protein (Flag) | NCBI |
| *Nephila inaurata* | AF218624.1 | flagelliform silk protein (Flag) | NCBI |
| *Nephila inaurata* | AF350278 | major ampullate spidroin 2 (MaSp2) gene, partial cds. | NCBI |
| *Nephila inaurata* | DQ059135.1 | major ampullate spidroin 2-like | NCBI |
| *Nephila inaurata* | AF350276 | major ampullate spidroin 2-like protein gene, partial cds. | NCBI |
| *Nephila pilipes* | AY666069 | clone NP-RTPD01 dragline silk spidroin 1 mRNA, partial cds. | NCBI |
| *Nephila pilipes* | AY666070 | clone NP-RTPD03 dragline silk spidroin 1 mRNA, partial cds. | NCBI |
| *Nephila pilipes* | AY666071 | clone NP-RTPD08 dragline silk spidroin 1 mRNA, partial cds. | NCBI |
| *Nephila pilipes* | AY666049 | clone NP02-PS02 dragline silk spidroin 1 gene, partial cds. | NCBI |
| *Nephila pilipes* | AY666050 | clone NP02-PS08 dragline silk spidroin 1 gene, partial cds. | NCBI |
| *Nephila pilipes* | AY666072 | clone NP072-RTPS01 dragline silk spidroin 1 mRNA, partial cds. | NCBI |
| *Nephila pilipes* | AY666073 | clone NP072-RTPS02 dragline silk spidroin 1 mRNA, partial cds. | NCBI |
| *Nephila pilipes* | AY666077 | clone NP072-RTPS10 dragline silk spidroin 1 mRNA, partial cds. | NCBI |
| *Nephila pilipes* | AY666074 | clone NP072-RTPS35 dragline silk spidroin 1 mRNA, partial cds. | NCBI |
| *Nephila pilipes* | AY666075 | clone NP072-RTPS39 dragline silk spidroin 1 mRNA, partial cds. | NCBI |
| *Nephila pilipes* | AY666076 | clone NP072-RTPS40 dragline silk spidroin 1 mRNA, partial cds. | NCBI |
| *Nephila pilipes* | AY666051 | clone NP11-SS01 dragline silk spidroin 1 gene, partial cds. | NCBI |
| *Nephila pilipes* | AY666052 | clone NP11-SS05 dragline silk spidroin 1 gene, partial cds. | NCBI |
| *Nephila pilipes* | AY666053 | clone NP11-SS08 dragline silk spidroin 1 gene, partial cds. | NCBI |
| *Nephila pilipes* | AY666054 | clone NP13-SS01 dragline silk spidroin 1 gene, partial cds. | NCBI |
| *Nephila pilipes* | AY666055 | clone NP13-SS04 dragline silk spidroin 1 gene, partial cds. | NCBI |
| *Nephila pilipes* | AY666056 | clone NP13-SS05 dragline silk spidroin 1 gene, partial cds. | NCBI |
| *Nephila pilipes* | AY666048 | clone NP13-SS07 dragline silk spidroin 1 gene, partial cds. | NCBI |
| *Nephila senegalensis* | AF350279 | major ampullate spidroin 1 (MaSp1) gene, partial cds. | NCBI |
| *Nephila senegalensis* | AF350280.1 | major ampullate spidroin 2 (MaSp2) | NCBI |
| *Nephila senegalensis* | AF516694 | major ampullate gland peroxidase mRNA, complete cds. | NCBI |
| *Nephilingis cruentata* | EF638446 | major ampullate spidroin-like protein mRNA, partial cds. | NCBI |
| *Nephilingis cruentata* | EF638448 | clone 06A01 minor ampullate spidroin-like protein mRNA, partial cds. | NCBI |
| *Nephilingis cruentata* | EF638449 | clone 11F12 minor ampullate spidroin-like protein mRNA, partial cds. | NCBI |
| *Nephilingis cruentata* | EF638447 | clone 11H11 minor ampullate spidroin-like protein mRNA, partial cds. | NCBI |
| *Nephilingis cruentata* | EF638444 | flagelliform spidroin-like protein mRNA, partial cds. | NCBI |
| *Nephilingis cruentata* | GU062417 | piriform-like spidroin mRNA, partial cds. | NCBI |
| *Nephilingis cruentata* | EF638445 | tubuliform spidroin-like protein mRNA, partial cds. | NCBI |
| *Parasteatoda tepidariorum* | MF955693 | Major ampullate Spidroin 3 C-terminal variant 1 | Ayoub et al. 2007 |
| *Steatoda grossa* | AY953090 | isolate SgrosR tubuliform spidroin 1 gene, partial cds. | NCBI |

**Supplemental Table S7**

Results of spectral clustering of spidroin genes in Araneoidea for Fig. 2c

| % area fraction: 0.192548 |  |  |  |
| --- | --- | --- | --- |
| % mass fraction: 0.992447 |  |  |  |
| % modularity: 0.652046 |  |  |  |
| % number of clusters: 9 |  |  |  |
| **Spidroin subset name\|Accession No,\|Organism\|Family** | **Cluster No.** | **Category name** | **Memo** |
| MaSp3\|g7867.t1\|MaSp3\|Araneus_ventricosus\|Araneidae | 1 | **MaSp-MiSp** | This study |
| MaSp2A\|g171745.t1\|MaSp2A\|Araneus_ventricosus\|Araneidae | 1 | **MaSp-MiSp** | This study |
| MaSp2B\|g171746.t1\|MaSp2B\|Araneus_ventricosus\|Araneidae | 1 | **MaSp-MiSp** | This study |
| MaSp1\|g121555.t1\|Araneus_ventricosus\|Araneidae | 1 | **MaSp-MiSp** | This study |
| MiSpA\|g264011.t1\|MiSpA\|Araneus_ventricosus\|Araneidae | 1 | **MaSp-MiSp** | This study |
| MiSpB\|g186744.t1\|MiSpB\|Araneus_ventricosus\|Araneidae | 1 | **MaSp-MiSp** | This study |
| Dragline\|AY666074\|Nephila_pilipes\|Nephilidae | 1 | **MaSp-MiSp** |  |
| MaSp1\|HQ005804.1_ADV40100.1\|Latrodectus_hesperus\|Theridiidae | 1 | **MaSp-MiSp** |  |
| MaSp1\|EU177650_ABY67405.1\|Latrodectus_hesperus\|Theridiidae | 1 | **MaSp-MiSp** |  |
| MaSp1\|AF350279\|Nephila_senegalensis\|Nephilidae | 1 | **MaSp-MiSp** |  |
| MaSp1\|KF032719\|Cyrtophora_moluccensis\|Araneidae | 1 | **MaSp-MiSp** |  |
| MaSp1\|AH015064.1_AAZ15321.1\|Latrodectus_geometricus\|Theridiidae | 1 | **MaSp-MiSp** |  |
| Dragline\|AY666055\|Nephila_pilipes\|Nephilidae | 1 | **MaSp-MiSp** |  |
| MaSp1\|AF350273\|Latrodectus_geometricus\|Theridiidae | 1 | **MaSp-MiSp** |  |
| MaSp1\|AY654288\|Nephila_clavipes\|Nephilidae | 1 | **MaSp-MiSp** |  |
| MaSp1\|DQ059133S2\|Latrodectus_geometricus\|Theridiidae | 1 | **MaSp-MiSp** |  |
| MaSpb\|Babb_MaSp-b\|Nephila_clavipes\|Nephilidae | 1 | **MaSp-MiSp** |  |
| Masp2\|AF350278\|Nephila_inaurata\|Nephilidae | 1 | **MaSp-MiSp** |  |
| MaSp1\|EU177648.1_ABY67401.1\|Latrodectus_hesperus\|Theridiidae | 1 | **MaSp-MiSp** |  |
| Dragline\|AY666060\|Cyrtophora_moluccensis\|Araneidae | 1 | **MaSp-MiSp** |  |
| Dragline\|AY061814\|Nephila_clavipes\|Nephilidae | 1 | **MaSp-MiSp** |  |
| MiSp\|EF638447\|Nephilingis_cruentata\|Nephilidae | 1 | **MaSp-MiSp** |  |
| Dragline\|NCU37520\|Nephila_clavipes\|Nephilidae | 1 | **MaSp-MiSp** |  |
| Spidroin1\|NCU20329\|Nephila_clavipes\|Nephilidae | 1 | **MaSp-MiSp** |  |
| MaSp1\|JN857964\|Araneus_ventricosus\|Araneidae | 1 | **MaSp-MiSp** |  |
| MaSp2\|MF955811\|Araneus_diadematus\|Araneidae | 1 | **MaSp-MiSp** |  |
| MaSp1\|EU177649_ABY67403.1\|Latrodectus_hesperus\|Theridiidae | 1 | **MaSp-MiSp** |  |
| Dragline\|AY666077\|Nephila_pilipes\|Nephilidae | 1 | **MaSp-MiSp** |  |
| MaSp1\|DQ059134.1_AAZ15321.1\|Latrodectus_geometricus\|Theridiidae | 1 | **MaSp-MiSp** |  |
| MaSp1\|AF350262\|Argiope_aurantia\|Araneidae | 1 | **MaSp-MiSp** |  |
| Dragline\|AY666075\|Nephila_pilipes\|Nephilidae | 1 | **MaSp-MiSp** |  |
| MaSp1\|DQ338461\|Nephila_antipodiana\|Nephilidae | 1 | **MaSp-MiSp** |  |
| MaSp1\|DQ409057.1_ABD66602.1\|Latrodectus_hesperus\|Theridiidae | 1 | **MaSp-MiSp** |  |
| MaSp2\|AF350272\|Gasteracantha_cancriformis\|Araneidae | 1 | **MaSp-MiSp** |  |
| MaSp2\|EF595245\|Latrodectus_hesperus\|Theridiidae | 1 | **MaSp-MiSp** |  |
| Dragline\|AY666054\|Nephila_pilipes\|Nephilidae | 1 | **MaSp-MiSp** |  |
| Dragline\|AY666051\|Nephila_pilipes\|Nephilidae | 1 | **MaSp-MiSp** |  |
| MaSp2\|DQ059136S2\|Argiope_trifasciata\|Araneidae | 1 | **MaSp-MiSp** |  |
| Dragline\|NEPFIBPR\|Nephila_clavipes\|Nephilidae | 1 | **MaSp-MiSp** |  |
| Dragline\|AY666079\|Cyrtophora_moluccensis\|Araneidae | 1 | **MaSp-MiSp** |  |
| MaSp1\|EU617338\|Nephila_clavipes\|Nephilidae | 1 | **MaSp-MiSp** |  |
| MaSp1\|AF350277\|Nephila_inaurata\|Nephilidae | 1 | **MaSp-MiSp** |  |
| MaSp2\|AY365017\|Argiope_amoena\|Araneidae | 1 | **MaSp-MiSp** |  |
| MaSp\|AY654291\|Nephila_clavipes\|Nephilidae | 1 | **MaSp-MiSp** |  |
| MaSpa\|Babb_MaSp-a\|Nephila_clavipes\|Nephilidae | 1 | **MaSp-MiSp** |  |
| MaSp2s\|AH015065.1_AAZ15372.1\|Argiope_trifasciata\|Araneidae | 1 | **MaSp-MiSp** |  |
| MaSp\|EU177653_ABY67411.1\|Latrodectus_hesperus\|Theridiidae | 1 | **MaSp-MiSp** |  |
| MaSp1\|AY654290\|Nephila_clavipes\|Nephilidae | 1 | **MaSp-MiSp** |  |
| MaSp2\|AY953075\|Latrodectus_hesperus\|Theridiidae | 1 | **MaSp-MiSp** |  |
| Fibroin2\|ADU47854\|Araneus_diadematus\|Araneidae | 1 | **MaSp-MiSp** |  |
| MaSp1\|AY654289\|Nephila_clavipes\|Nephilidae | 1 | **MaSp-MiSp** |  |
| Dragline\|AY666081\|Cyrtophora_moluccensis\|Araneidae | 1 | **MaSp-MiSp** |  |
| MaSp2\|DQ409058\|Latrodectus_hesperus\|Theridiidae | 1 | **MaSp-MiSp** |  |
| Dragline\|AY666076\|Nephila_pilipes\|Nephilidae | 1 | **MaSp-MiSp** |  |
| MaSp1\|AY263390\|Argiope_amoena\|Araneidae | 1 | **MaSp-MiSp** |  |
| MaSp2\|AY365016\|Argiope_amoena\|Araneidae | 1 | **MaSp-MiSp** |  |
| Dragline\|M92913.1_AAA29381.1\|Nephila_clavipes\|Nephilidae | 1 | **MaSp-MiSp** |  |
| Fibroin4\|ADU47856\|Araneus_diadematus\|Araneidae | 1 | **MaSp-MiSp** |  |
| MaSp2\|AY365021\|Argiope_amoena\|Araneidae | 1 | **MaSp-MiSp** |  |
| MaSpc\|Babb_MaSp-c\|Nephila_clavipes\|Nephilidae | 1 | **MaSp-MiSp** |  |
| Dragline\|NEPDSF\|Nephila_clavipes\|Nephilidae | 1 | **MaSp-MiSp** |  |
| Dragline\|AF441245\|Nephila_clavata\|Nephilidae | 1 | **MaSp-MiSp** |  |
| Dragline\|AY666061\|Cyrtophora_moluccensis\|Araneidae | 1 | **MaSp-MiSp** |  |
| Dragline\|AY666072\|Nephila_pilipes\|Nephilidae | 1 | **MaSp-MiSp** |  |
| MaSp2\|AF350263\|Argiope_aurantia\|Araneidae | 1 | **MaSp-MiSp** |  |
| MaSp2\|JX112872\|Argiope_bruennichi\|Araneidae | 1 | **MaSp-MiSp** |  |
| Spidroin\|NCU03848\|Nephila_clavipes\|Nephilidae | 1 | **MaSp-MiSp** |  |
| MaSp\|EF638446\|Nephilingis_cruentata\|Nephilidae | 1 | **MaSp-MiSp** |  |
| MaSp2\|AY654295\|Nephila_clavipes\|Nephilidae | 1 | **MaSp-MiSp** |  |
| Dragline\|AY666080\|Cyrtophora_moluccensis\|Araneidae | 1 | **MaSp-MiSp** |  |
| Spidroin\|ABU03847\|Araneus_bicentenarius\|Araneidae | 1 | **MaSp-MiSp** |  |
| MaSpf1\|Babb_MaSp-f-1\|Nephila_clavipes\|Nephilidae | 1 | **MaSp-MiSp** |  |
| MaSp1\|AF350266\|Argiope_trifasciata\|Araneidae | 1 | **MaSp-MiSp** |  |
| Spidroin2\|ABU20328\|Araneus_bicentenarius\|Araneidae | 1 | **MaSp-MiSp** |  |
| Dragline\|AY666073\|Nephila_pilipes\|Nephilidae | 1 | **MaSp-MiSp** |  |
| MaSp1\|EF595246\|Latrodectus_hesperus\|Theridiidae | 1 | **MaSp-MiSp** |  |
| MaSp2\|AF350275\|Latrodectus_geometricus\|Theridiidae | 1 | **MaSp-MiSp** |  |
| MaSp2\|AY365018\|Argiope_amoena\|Araneidae | 1 | **MaSp-MiSp** |  |
| Dragline\|AY666063\|Cyrtophora_moluccensis\|Araneidae | 1 | **MaSp-MiSp** |  |
| MaSp\|AY654292\|Nephila_clavipes\|Nephilidae | 1 | **MaSp-MiSp** |  |
| MaSp2\|DQ059137.1_AAZ15372.1\|Argiope_trifasciata\|Araneidae | 1 | **MaSp-MiSp** |  |
| Dragline\|AY666053\|Nephila_pilipes\|Nephilidae | 1 | **MaSp-MiSp** |  |
| MaSp2\|AY365015\|Argiope_amoena\|Araneidae | 1 | **MaSp-MiSp** |  |
| Dragline\|AY666062\|Cyrtophora_moluccensis\|Araneidae | 1 | **MaSp-MiSp** |  |
| MaSp2\|AY365020\|Argiope_amoena\|Araneidae | 1 | **MaSp-MiSp** |  |
| MaSp2\|AY654293\|Nephila_clavipes\|Nephilidae | 1 | **MaSp-MiSp** |  |
| MaSp3\|MF955692\|Latrodectus_hesperus\|Theridiidae | 1 | **MaSp-MiSp** |  |
| MaSp2\|AY654296\|Nephila_clavipes\|Nephilidae | 1 | **MaSp-MiSp** |  |
| MaSp2\|AF350280\|Nephila_senegalensis\|Nephilidae | 1 | **MaSp-MiSp** |  |
| MaSp2\|AY365019\|Argiope_amoena\|Araneidae | 1 | **MaSp-MiSp** |  |
| Dragline\|AY666050\|Nephila_pilipes\|Nephilidae | 1 | **MaSp-MiSp** |  |
| Dragline\|AY666078\|Cyrtophora_moluccensis\|Araneidae | 1 | **MaSp-MiSp** |  |
| Dragline\|M37137.2_AAA29380.2\|Nephila_clavipes\|Nephilidae | 1 | **MaSp-MiSp** |  |
| MaSpg\|Babb_MaSp-g\|Nephila_clavipes\|Nephilidae | 1 | **MaSp-MiSp** |  |
| MaSp2\|AY654297\|Nephila_clavipes\|Nephilidae | 1 | **MaSp-MiSp** |  |
| MaSp1\|JX112871\|Argiope_bruennichi\|Araneidae | 1 | **MaSp-MiSp** |  |
| Dragline\|AY666049\|Nephila_pilipes\|Nephilidae | 1 | **MaSp-MiSp** |  |
| MaSp1\|AY953074\|Latrodectus_hesperus\|Theridiidae | 1 | **MaSp-MiSp** |  |
| MaSph\|Babb_MaSp-h\|Nephila_clavipes\|Nephilidae | 1 | **MaSp-MiSp** |  |
| Dragline\|AY666048\|Nephila_pilipes\|Nephilidae | 1 | **MaSp-MiSp** |  |
| Dragline\|AY666056\|Nephila_pilipes\|Nephilidae | 1 | **MaSp-MiSp** |  |
| Dragline\|AY666052\|Nephila_pilipes\|Nephilidae | 1 | **MaSp-MiSp** |  |
| Fibroin3\|ADU47855\|Araneus_diadematus\|Araneidae | 1 | **MaSp-MiSp** |  |
| MaSp2\|AY654294\|Nephila_clavipes\|Nephilidae | 1 | **MaSp-MiSp** |  |
| MaSp2\|AF350267\|Argiope_trifasciata\|Araneidae | 1 | **MaSp-MiSp** |  |
| MaSp1\|EU177655_ABY67415.1\|Latrodectus_hesperus\|Theridiidae | 1 | **MaSp-MiSp** |  |
| MaSp2\|JX202781\|Argiope_bruennichi\|Araneidae | 1 | **MaSp-MiSp** |  |
| MaSp1\|EU177651_ABY67407.1\|Latrodectus_hesperus\|Theridiidae | 1 | **MaSp-MiSp** |  |
| MaSp1\|EU177654_ABY67413.1\|Latrodectus_hesperus\|Theridiidae | 1 | **MaSp-MiSp** |  |
| MaSp1\|EU177650_ABY67404.1\|Latrodectus_hesperus\|Theridiidae | 1 | **MaSp-MiSp** |  |
| MaSp1\|EU177669\|Latrodectus_geometricus\|Theridiidae | 1 | **MaSp-MiSp** |  |
| MaSp2\|EU177652_ABY67408.1\|Latrodectus_hesperus\|Theridiidae | 1 | **MaSp-MiSp** |  |
| MaSp1\|EU177663\|Latrodectus_hesperus\|Theridiidae | 1 | **MaSp-MiSp** |  |
| MaSp1A\|EU599241\|Nephila_clavipes\|Nephilidae | 1 | **MaSp-MiSp** |  |
| MaSp\|EU177653_ABY67410.1\|Latrodectus_hesperus\|Theridiidae | 1 | **MaSp-MiSp** |  |
| MaSp1\|EU177648.1_ABY67400.1\|Latrodectus_hesperus\|Theridiidae | 1 | **MaSp-MiSp** |  |
| MaSp1\|EU177660.1_ABY67420.1\|Latrodectus_geometricus\|Theridiidae | 1 | **MaSp-MiSp** |  |
| MaSp1\|EU177664\|Latrodectus_hesperus\|Theridiidae | 1 | **MaSp-MiSp** |  |
| MaSp1\|EU177667\|Latrodectus_geometricus\|Theridiidae | 1 | **MaSp-MiSp** |  |
| MaSp1\|EU177668\|Latrodectus_geometricus\|Theridiidae | 1 | **MaSp-MiSp** |  |
| MaSp1\|DQ379381\|Latrodectus_hesperus\|Theridiidae | 1 | **MaSp-MiSp** |  |
| MaSp1\|HM752779\|Latrodectus_mactans\|Theridiidae | 1 | **MaSp-MiSp** |  |
| MaSp1\|EU177654_ABY67412.1\|Latrodectus_hesperus\|Theridiidae | 1 | **MaSp-MiSp** |  |
| MaSp1\|EU177666\|Latrodectus_geometricus\|Theridiidae | 1 | **MaSp-MiSp** |  |
| MaSp1\|EU177651_ABY67406.1\|Latrodectus_hesperus\|Theridiidae | 1 | **MaSp-MiSp** |  |
| MaSp1\|EU177649_ABY67402.1\|Latrodectus_hesperus\|Theridiidae | 1 | **MaSp-MiSp** |  |
| MaSp2\|EF595248\|Latrodectus_hesperus\|Theridiidae | 1 | **MaSp-MiSp** |  |
| MaSp1\|EU177659\|Latrodectus_hesperus\|Theridiidae | 1 | **MaSp-MiSp** |  |
| MaSp1\|EU177655_ABY67414.1\|Latrodectus_hesperus\|Theridiidae | 1 | **MaSp-MiSp** |  |
| MaSp1\|EU177658\|Latrodectus_hesperus\|Theridiidae | 1 | **MaSp-MiSp** |  |
| MaSp3\|MF955786\|Latrodectus_hesperus\|Theridiidae | 1 | **MaSp-MiSp** |  |
| MaSp1\|EF595247\|Latrodectus_hesperus\|Theridiidae | 1 | **MaSp-MiSp** |  |
| MaSp1\|EU177661\|Latrodectus_hesperus\|Theridiidae | 1 | **MaSp-MiSp** |  |
| MaSp1\|EU177665.1_ABY67425.1\|Latrodectus_hesperus\|Theridiidae | 1 | **MaSp-MiSp** |  |
| MaSp3\|MF955693\|Parasteatoda_tepidariorum\|Theridiidae | 1 | **MaSp-MiSp** |  |
| MiSp\|AF027736\|Nephila_clavipes\|Nephilidae | 1 | **MaSp-MiSp** |  |
| MiSp\|Babb_MiSp-c\|Nephila_clavipes\|Nephilidae | 1 | **MaSp-MiSp** |  |
| MaSp2\|MF955809\|Araneus_diadematus\|Araneidae | 1 | **MaSp-MiSp** |  |
| MiSp\|JX513956\|Araneus_ventricosus\|Araneidae | 1 | **MaSp-MiSp** |  |
| MaSp2\|EU177657\|Latrodectus_geometricus\|Theridiidae | 1 | **MaSp-MiSp** |  |
| MaSp1A\|EU599238\|Nephila_clavipes\|Nephilidae | 1 | **MaSp-MiSp** |  |
| MaSp1\|EU177662\|Latrodectus_hesperus\|Theridiidae | 1 | **MaSp-MiSp** |  |
| MaSp2\|DQ379382\|Latrodectus_hesperus\|Theridiidae | 1 | **MaSp-MiSp** |  |
| MaSp1B\|EU599242.1_ACF19415.1\|Nephila_clavipes\|Nephilidae | 1 | **MaSp-MiSp** |  |
| MaSp1\|AH015064.1_AAZ15320.1\|Latrodectus_geometricus\|Theridiidae | 1 | **MaSp-MiSp** |  |
| MaSp1B\|EU599239\|Nephila_clavipes\|Nephilidae | 1 | **MaSp-MiSp** |  |
| MaSp\|DQ059133S1\|Latrodectus_geometricus\|Theridiidae | 1 | **MaSp-MiSp** |  |
| MaSp2\|EU599243\|Nephila_clavipes\|Nephilidae | 1 | **MaSp-MiSp** |  |
| MiSp\|HM752569\|Metepeira_grandiosa\|Araneidae | 1 | **MaSp-MiSp** |  |
| MaSp2\|EU599240\|Nephila_clavipes\|Nephilidae | 1 | **MaSp-MiSp** |  |
| MaSp2s\|AH015065.1_AAZ15371.1\|Argiope_trifasciata\|Araneidae | 1 | **MaSp-MiSp** |  |
| MaSp2\|DQ059136S1\|Argiope_trifasciata\|Araneidae | 1 | **MaSp-MiSp** |  |
| MaSp2\|DQ059135\|Nephila_inaurata\|Nephilidae | 1 | **MaSp-MiSp** |  |
| MaSpf2\|Babb_MaSp-f-2\|Nephila_clavipes\|Nephilidae | 1 | **MaSp-MiSp** |  |
| Fibroin1\|ADU47853\|Araneus_diadematus\|Araneidae | 1 | **MaSp-MiSp** |  |
| MiSp\|EF638449\|Nephilingis_cruentata\|Nephilidae | 1 | **MaSp-MiSp** |  |
| MiSpb\|Babb_MiSp-b\|Nephila_clavipes\|Nephilidae | 1 | **MaSp-MiSp** |  |
| MiSp1\|AF027735\|Nephila_clavipes\|Nephilidae | 1 | **MaSp-MiSp** |  |
| MiSp1\|DQ338462\|Nephila_antipodiana\|Nephilidae | 1 | **MaSp-MiSp** |  |
| MiSpa\|Babb_MiSp-a\|Nephila_clavipes\|Nephilidae | 1 | **MaSp-MiSp** |  |
| MiSpd\|Babb_MiSp-d\|Nephila_clavipes\|Nephilidae | 1 | **MaSp-MiSp** |  |
| MiSp\|HM752571\|Latrodectus_hesperus\|Theridiidae | 1 | **MaSp-MiSp** |  |
| MiSp\|KX584003_ARA91152.1\|Latrodectus_hesperus\|Theridiidae | 1 | **MaSp-MiSp** |  |
| MiSp1\|EU394445\|Latrodectus_hesperus\|Theridiidae | 1 | **MaSp-MiSp** |  |
| MaSp2\|MF955810\|Araneus_diadematus\|Araneidae | 1 | **MaSp-MiSp** |  |
| MiSp\|JQ713003\|Argiope_argentata\|Araneidae | 1 | **MaSp-MiSp** |  |
| MaSp2\|AY177203\|Araneus_ventricosus\|Araneidae | 1 | **MaSp-MiSp** |  |
| MaSp2\|AF350276\|Nephila_inaurata\|Nephilidae | 1 | **MaSp-MiSp** |  |
| MaSpd\|Babb_MaSp-d\|Nephila_clavipes\|Nephilidae | 1 | **MaSp-MiSp** |  |
| MiSp2\|JX513955\|Araneus_ventricosus\|Araneidae | 1 | **MaSp-MiSp** |  |
| MiSp1\|JX513954\|Araneus_ventricosus\|Araneidae | 1 | **MaSp-MiSp** |  |
| MiSp\|HM752570\|Latrodectus_hesperus\|Theridiidae | 1 | **MaSp-MiSp** |  |
| MaSp2\|EU177656\|Latrodectus_hesperus\|Theridiidae | 1 | **MaSp-MiSp** |  |
| MaSp2\|EU177652_ABY67409.1\|Latrodectus_hesperus\|Theridiidae | 1 | **MaSp-MiSp** |  |
| MaSp3\|MF955691\|Araneus_diadematus\|Araneidae | 1 | **MaSp-MiSp** |  |
| MaSp2\|AF350274\|Latrodectus_geometricus\|Theridiidae | 1 | **MaSp-MiSp** |  |
| MiSp\|JQ713004\|Argiope_argentata\|Araneidae | 1 | **MaSp-MiSp** |  |
| AcSp\|g246635.t1\|Araneus_ventricosus\|Araneidae | 2 | **AcSp** | This study |
| AcSp1\|KJ206613.1_AHK09806.1\|Argiope_trifasciata\|Araneidae | 2 | **AcSp** |  |
| AcSp1\|KJ206604.1_AHK09797.1\|Argiope_argentata\|Araneidae | 2 | **AcSp** |  |
| AcSp1\|KJ206605.1_AHK09798.1\|Argiope_argentata\|Araneidae | 2 | **AcSp** |  |
| AcSp1\|KJ206612.1_AHK09805.1\|Argiope_trifasciata\|Araneidae | 2 | **AcSp** |  |
| AcSp1\|KJ206602.1_AHK09795.1\|Argiope_argentata\|Araneidae | 2 | **AcSp** |  |
| AcSp1\|KJ206609.1_AHK09802.1\|Argiope_trifasciata\|Araneidae | 2 | **AcSp** |  |
| AcSp1\|KJ206619.1_AHK09812.1\|Argiope_trifasciata\|Araneidae | 2 | **AcSp** |  |
| AcSp1\|KJ206572.1_AHK09765.1\|Argiope_argentata\|Araneidae | 2 | **AcSp** |  |
| AcSp1\|KJ206608.1_AHK09801.1\|Argiope_aurantia\|Araneidae | 2 | **AcSp** |  |
| AcSp1\|KJ206576.1_AHK09769.1\|Argiope_argentata\|Araneidae | 2 | **AcSp** |  |
| AcSp1\|KJ206603.1_AHK09796.1\|Argiope_argentata\|Araneidae | 2 | **AcSp** |  |
| AcSp1\|KJ206615.1_AHK09808.1\|Argiope_trifasciata\|Araneidae | 2 | **AcSp** |  |
| AcSp\|AY426339\|Argiope_trifasciata\|Araneidae | 2 | **AcSp** |  |
| AcSp1\|KJ206620.1_AHK09813.1\|Argiope_argentata\|Araneidae | 2 | **AcSp** |  |
| AcSp1\|KJ206597.1_AHK09790.1\|Argiope_argentata\|Araneidae | 2 | **AcSp** |  |
| AcSp1\|KJ206600.1_AHK09793.1\|Argiope_argentata\|Araneidae | 2 | **AcSp** |  |
| AcSp1\|KJ206616.1_AHK09809.1\|Argiope_trifasciata\|Araneidae | 2 | **AcSp** |  |
| AcSp1\|KJ206571.1_AHK09764.1\|Argiope_argentata\|Araneidae | 2 | **AcSp** |  |
| AcSp1\|KJ206610.1_AHK09803.1\|Argiope_trifasciata\|Araneidae | 2 | **AcSp** |  |
| AcSp1\|KJ206607.1_AHK09800.1\|Argiope_aurantia\|Araneidae | 2 | **AcSp** |  |
| AcSp1\|KJ206618.1_AHK09811.1\|Argiope_trifasciata\|Araneidae | 2 | **AcSp** |  |
| AcSp1\|KJ206614.1_AHK09807.1\|Argiope_trifasciata\|Araneidae | 2 | **AcSp** |  |
| AcSp1\|KJ206611.1_AHK09804.1\|Argiope_trifasciata\|Araneidae | 2 | **AcSp** |  |
| AcSp1\|KJ206617.1_AHK09810.1\|Argiope_trifasciata\|Araneidae | 2 | **AcSp** |  |
| AcSp1\|KJ206606.1_AHK09799.1\|Argiope_argentata\|Araneidae | 2 | **AcSp** |  |
| AcSp1\|JX978178\|Latrodectus_hesperus\|Theridiidae | 2 | **AcSp** |  |
| AcSp1\|JX978175\|Latrodectus_hesperus\|Theridiidae | 2 | **AcSp** |  |
| AcSp1\|JX978181\|Latrodectus_geometricus\|Theridiidae | 2 | **AcSp** |  |
| AcSp1\|JX978176\|Latrodectus_hesperus\|Theridiidae | 2 | **AcSp** |  |
| AcSp1\|JX978177\|Latrodectus_hesperus\|Theridiidae | 2 | **AcSp** |  |
| AcSp1\|JX978172\|Latrodectus_hesperus\|Theridiidae | 2 | **AcSp** |  |
| AcSp\|EU025854\|Latrodectus_hesperus\|Theridiidae | 2 | **AcSp** |  |
| AcSp1\|JX978182\|Latrodectus_geometricus\|Theridiidae | 2 | **AcSp** |  |
| AcSp1\|JX978171\|Latrodectus_hesperus\|Theridiidae | 2 | **AcSp** |  |
| AcSp1\|JX978174.1_AFX83560.1\|Latrodectus_hesperus\|Theridiidae | 2 | **AcSp** |  |
| AcSp1\|JX978173\|Latrodectus_hesperus\|Theridiidae | 2 | **AcSp** |  |
| AcSp1\|JX978179\|Latrodectus_hesperus\|Theridiidae | 2 | **AcSp** |  |
| AcSp1\|KJ206594.1_AHK09787.1\|Argiope_trifasciata\|Araneidae | 2 | **AcSp** |  |
| AcSp1\|HQ008715\|Argiope_amoena\|Araneidae | 2 | **AcSp** |  |
| AcSp1\|KJ206595.1_AHK09788.1\|Argiope_trifasciata\|Araneidae | 2 | **AcSp** |  |
| AcSp1\|KJ206590.1_AHK09783.1\|Argiope_trifasciata\|Araneidae | 2 | **AcSp** |  |
| AcSp1\|KJ206588.1_AHK09781.1\|Argiope_trifasciata\|Araneidae | 2 | **AcSp** |  |
| AcSp1\|KJ206592.1_AHK09785.1\|Argiope_trifasciata\|Araneidae | 2 | **AcSp** |  |
| AcSp1\|KJ206599.1_AHK09792.1\|Argiope_trifasciata\|Araneidae | 2 | **AcSp** |  |
| AcSp1\|KJ206591.1_AHK09784.1\|Argiope_trifasciata\|Araneidae | 2 | **AcSp** |  |
| AcSp1\|KJ206573.1_AHK09766.1\|Argiope_argentata\|Araneidae | 2 | **AcSp** |  |
| AcSp1\|KJ206574.1_AHK09767.1\|Argiope_argentata\|Araneidae | 2 | **AcSp** |  |
| AcSp1\|KJ206589.1_AHK09782.1\|Argiope_trifasciata\|Araneidae | 2 | **AcSp** |  |
| AcSp1\|KJ206584.1_AHK09777.1\|Argiope_trifasciata\|Araneidae | 2 | **AcSp** |  |
| AcSp\|Babb_AcSp\|Nephila_clavipes\|Nephilidae | 2 | **AcSp** |  |
| AcSp1\|KJ206581.1_AHK09774.1\|Argiope_aurantia\|Araneidae | 2 | **AcSp** |  |
| AcSp1\|KJ206582.1_AHK09775.1\|Argiope_aurantia\|Araneidae | 2 | **AcSp** |  |
| AcSp1\|KJ206596.1_AHK09789.1\|Argiope_trifasciata\|Araneidae | 2 | **AcSp** |  |
| AcSp1\|KJ206585.1_AHK09778.1\|Argiope_trifasciata\|Araneidae | 2 | **AcSp** |  |
| AcSp1\|KJ206577.1_AHK09770.1\|Argiope_aurantia\|Araneidae | 2 | **AcSp** |  |
| AcSp1\|KJ206586.1_AHK09779.1\|Argiope_trifasciata\|Araneidae | 2 | **AcSp** |  |
| AcSp1\|KJ206570.1_AHK09763.1\|Argiope_argentata\|Araneidae | 2 | **AcSp** |  |
| AcSp1\|KJ206587.1_AHK09780.1\|Argiope_trifasciata\|Araneidae | 2 | **AcSp** |  |
| AcSp1\|KJ206580.1_AHK09773.1\|Argiope_aurantia\|Araneidae | 2 | **AcSp** |  |
| AcSp1\|KJ206575.1_AHK09768.1\|Argiope_argentata\|Araneidae | 2 | **AcSp** |  |
| AcSp1\|KJ206593.1_AHK09786.1\|Argiope_trifasciata\|Araneidae | 2 | **AcSp** |  |
| AcSp1\|KJ206579.1_AHK09772.1\|Argiope_aurantia\|Araneidae | 2 | **AcSp** |  |
| AcSp1\|KJ206583.1_AHK09776.1\|Argiope_trifasciata\|Araneidae | 2 | **AcSp** |  |
| AcSp1\|KJ206578.1_AHK09771.1\|Argiope_aurantia\|Araneidae | 2 | **AcSp** |  |
| AcSp1\|JX978180.1_AFX83566.1\|Latrodectus_geometricus\|Theridiidae | 2 | **AcSp** |  |
| AcSp1\|HQ008714.1_ADM35668.1\|Araneus_ventricosus\|Araneidae | 2 | **AcSp** |  |
| AcSp1\|KJ206601.1_AHK09794.1\|Argiope_argentata\|Araneidae | 2 | **AcSp** |  |
| CySp\|g18336.t1\|CySp\|Araneus_ventricosus\|Araneidae | 3 | **CySp** | This study |
| TuSp\|AY953081\|Argiope_aurantia\|Araneidae | 3 | **CySp** |  |
| TuSp\|AY953082\|Gea_heptagon\|Araneidae | 3 | **CySp** |  |
| TuSp\|AY953093\|Gea_heptagon\|Araneidae | 3 | **CySp** |  |
| TuSp\|AY855099\|Argiope_aurantia\|Araneidae | 3 | **CySp** |  |
| CySp2\|AB242145\|Argiope_bruennichi\|Araneidae | 3 | **CySp** |  |
| TuSp\|HM752577\|Argiope_argentata\|Araneidae | 3 | **CySp** |  |
| TuSp\|AY953084\|Argiope_argentata\|Araneidae | 3 | **CySp** |  |
| TuSp1\|JQ291306\|Argiope_amoena\|Araneidae | 3 | **CySp** |  |
| TuSp\|AY953085\|Latrodectus_mactans\|Theridiidae | 3 | **CySp** |  |
| TuSp\|AY855098\|Argiope_aurantia\|Araneidae | 3 | **CySp** |  |
| TuSp1\|AY953083\|Cyrtophora_moluccensis\|Araneidae | 3 | **CySp** |  |
| TuSp\|AY855100\|Araneus_gemmoides\|Araneidae | 3 | **CySp** |  |
| TuSp1\|AY953071\|Argiope_argentata\|Araneidae | 3 | **CySp** |  |
| Egg\|EU730637\|Nephila_antipodiana\|Nephilidae | 3 | **CySp** |  |
| TuSp1\|AY953092\|Argiope_aurantia\|Araneidae | 3 | **CySp** |  |
| CySp1\|AB242144\|Argiope_bruennichi\|Araneidae | 3 | **CySp** |  |
| TuSp\|AY953086\|Latrodectus_hesperus\|Theridiidae | 3 | **CySp** |  |
| TuSp1\|AY953091\|Argiope_argentata\|Araneidae | 3 | **CySp** |  |
| TuSp1\|AY953090\|Steatoda_grossa\|Theridiidae | 3 | **CySp** |  |
| TuSp\|DQ379383\|Latrodectus_hesperus\|Theridiidae | 3 | **CySp** |  |
| TuSp\|AY855101\|Araneus_gemmoides\|Araneidae | 3 | **CySp** |  |
| TuSp1\|AY953088\|Latrodectus_hasselti\|Theridiidae | 3 | **CySp** |  |
| TuSp\|AY953089\|Latrodectus_geometricus\|Theridiidae | 3 | **CySp** |  |
| TuSp\|EF638445\|Nephilingis_cruentata\|Nephilidae | 3 | **CySp** |  |
| Egg\|DQ109035\|Latrodectus_hesperus\|Theridiidae | 3 | **CySp** |  |
| TuSp\|AY953087\|Latrodectus_tredecimguttatus\|Theridiidae | 3 | **CySp** |  |
| TuSp1\|AY953070\|Latrodectus_hesperus\|Theridiidae | 3 | **CySp** |  |
| TuSp\|AY953077\|Latrodectus_mactans\|Theridiidae | 3 | **CySp** |  |
| TuSp\|AY953078\|Latrodectus_tredecimguttatus\|Theridiidae | 3 | **CySp** |  |
| TuSp\|AY953076\|Latrodectus_hesperus\|Theridiidae | 3 | **CySp** |  |
| TuSp\|AY953079\|Latrodectus_geometricus\|Theridiidae | 3 | **CySp** |  |
| Egg\|HQ005885.1_ADV40181.1\|Latrodectus_hesperus\|Theridiidae | 3 | **CySp** |  |
| TuSp1\|AY953080\|Latrodectus_hasselti\|Theridiidae | 3 | **CySp** |  |
| TuSp1\|HQ005889.1_ADV40185.1\|Latrodectus_hesperus\|Theridiidae | 3 | **CySp** |  |
| CySp1\|AB218974\|Nephila_clavata\|Nephilidae | 3 | **CySp** |  |
| CySp1\|AB218973\|Nephila_clavata\|Nephilidae | 3 | **CySp** |  |
| TuSp\|AY855102\|Nephila_clavipes\|Nephilidae | 3 | **CySp** |  |
| CySp\|Babb_CySp\|Nephila_clavipes\|Nephilidae | 3 | **CySp** |  |
| Flag\|g100465.t1\|Araneus_ventricosus\|Araneidae | 4 | **Flag** | This study |
| Flag\|AF218622.1_AAF36089.1\|Nephila_clavipes\|Nephilidae | 4 | **Flag** |  |
| Flag\|EF638444\|Nephilingis_cruentata\|Nephilidae | 4 | **Flag** |  |
| Flag\|AH009147.2_AAF36092.1\|Nephila_inaurata\|Nephilidae | 4 | **Flag** |  |
| Flag\|EF025541\|Araneus_ventricosus\|Araneidae | 4 | **Flag** |  |
| Flag\|AH009147.2_AAF36091.1\|Nephila_inaurata\|Nephilidae | 4 | **Flag** |  |
| Flag\|AF027972\|Nephila_clavipes\|Nephilidae | 4 | **Flag** |  |
| Flaga\|Babb_FLAG-a\|Nephila_clavipes\|Nephilidae | 4 | **Flag** |  |
| Flag\|AF218623.1_AAF36091.1\|Nephila_inaurata\|Nephilidae | 4 | **Flag** |  |
| Flag\|AF218621S2\|Nephila_clavipes\|Nephilidae | 4 | **Flag** |  |
| Flag\|AF218624.1_AAF36092.1\|Nephila_inaurata\|Nephilidae | 4 | **Flag** |  |
| Flag\|AH009146.1_AAF36090.1\|Nephila_clavipes\|Nephilidae | 4 | **Flag** |  |
| Flag\|AF027973.1_AAC38847.1\|Nephila_clavipes\|Nephilidae | 4 | **Flag** |  |
| Flag\|AH009146.1_AAF36089.1\|Nephila_clavipes\|Nephilidae | 4 | **Flag** |  |
| Flag\|Babb_FLAG-b\|Nephila_clavipes\|Nephilidae | 4 | **Flag** |  |
| MaSp1\|AY174110\|Araneus_ventricosus\|Araneidae | 4 | **Flag** |  |
| MaSp\|AY945306\|Araneus_ventricosus\|Araneidae | 4 | **Flag** |  |
| MaSp2\|AF350264.1_AAK30593.1\|Argiope_trifasciata\|Araneidae | 4 | **Flag** |  |
| Flag\|AY587193\|Araneus_ventricosus\|Araneidae | 4 | **Flag** |  |
| PySp\|g16081.t1\|Araneus_ventricosus\|Araneidae | 5 | **PySp** | This study |
| PySp\|FJ973621\|Latrodectus_hesperus\|Theridiidae | 5 | **PySp** |  |
| PySp1\|HQ005791.1_ADV40087.1\|Latrodectus_hesperus\|Theridiidae | 5 | **PySp** |  |
| PySp\|GQ980328\|Argiope_trifasciata\|Araneidae | 5 | **PySp** |  |
| PySp\|GQ980330\|Nephila_clavipes\|Nephilidae | 5 | **PySp** |  |
| PySp\|Babb_PiSp\|Nephila_clavipes\|Nephilidae | 5 | **PySp** |  |
| PySp\|GU062417\|Nephilingis_cruentata\|Nephilidae | 5 | **PySp** |  |
| PySp2\|HM020705\|Nephila_clavipes\|Nephilidae | 5 | **PySp** |  |
| PySp\|JF827034\|Araneus_gemmoides\|Araneidae | 5 | **PySp** |  |
| Agg\|g118932.t1\|Araneus_ventricosus\|Araneidae | 8 | **AgSp** | This study |
| Agg2\|EU780015.1_ACI41239.1\|Nephila_clavipes\|Nephilidae | 8 | **AgSp** |  |
| AgSpc\|Babb_AgSp-c\|Nephila_clavipes\|Nephilidae | 8 | **AgSp** |  |
| Dragline\|AY666071\|Nephila_pilipes\|Nephilidae | 6 | **Spidroin6** |  |
| Dragline\|AY666070\|Nephila_pilipes\|Nephilidae | 6 | **Spidroin6** |  |
| Dragline\|AY666069\|Nephila_pilipes\|Nephilidae | 6 | **Spidroin6** |  |
| Egg2\|HQ005853.1_ADV40149.1\|Latrodectus_hesperus\|Theridiidae | 7 | **Spidroin7** |  |
| Egg\|DQ341220\|Latrodectus_hesperus\|Theridiidae | 7 | **Spidroin7** |  |
| Egg\|AY994149\|Latrodectus_hesperus\|Theridiidae | 7 | **Spidroin7** |  |
| Sp-74867\|Babb_Sp-74867\|Nephila_clavipes\|Nephilidae | 9 | **Spidroin9** |  |
| Sp-907\|Babb_Sp-907\|Nephila_clavipes\|Nephilidae | 9 | **Spidroin9** |  |

**Supplemental Table S8**

Sampling and sequencing information in Fig. 3

| **Family** | **Species** | **Sampling and sequencing references** | **Geographical coordinate** | **Japanese Prefecture** | **# of reads** | **Accession number** | **Assembled file at figshare** |
| --- | --- | --- | --- | --- | --- | --- | --- |
| Araneidae | *Gasteracantha kuhli* | This study | 40.936767,140.322398 | Aomori | 31,727,686 | DRR129307 | https://figshare.com/s/a3dae7e69c39fdd21a77 |
| Araneidae | *Argiope aemula* | This study | 26.784902,128.284998 | Okinawa | 37,612,900 | DRR129308 | https://figshare.com/s/40eb4010e3ff62b7292a |
| Araneidae | *Cyrtophora unicolor* | This study | 26.784902,128.284998 | Okinawa | 37,297,000 | DRR129309 | https://figshare.com/s/7ae476cf096b14e2e296 |
| Araneidae | *Zygiella dispar* | This study | 43.297955,143.116996 | Hokkaido | 44,536,676 | DRR129310 | https://figshare.com/s/bba73a2f1554105caeec |
| Araneidae | *Araneus seminiger* | This study | 26.802394,128.271628 | Okinawa | 37,089,200 | DRR129311 | https://figshare.com/s/b27bc971099ae73a3ca9 |
| Araneidae | *Cyclosa octotuberculata* | This study | 38.752695,139.742315 | Yamagata | 55,152,252 | DRR129312 | https://figshare.com/s/d72e28beb5ce4ff994e8 |
| Araneidae | *Gasteracantha hasselti* | SRX360523 | - | - | 12,564,452 | SRR1048659 | - |
| Araneidae | *Macracantha arcuata* | SRX360519 | - | - | 17,523,883 | SRR1048826 | - |
| Araneidae | *Micrathena gracilis* | SRX652498 | - | - | 56,963,267 | SRR1514882 | - |
| Araneidae | *Verrucosa arenata* | SRX1560156 | - | - | 14,468,378 | SRR3144087 | - |
| Araneidae | *Nephila clavipes* | SRX2458092 | - | - | 94,012,993 | SRR5139327 | - |
| Buthidae | *Centruroides vittatus* | SRX2635386 | - | - | 23,410,736 | SRR5338072 | - |
| Linyphiidae | *Frontinella communis* | SRX451004 | - | - | 33,091,580 | SRR1145739 | - |
| Nesticidae | *Neoscona theisi* | This study | 26.784902,128.284998 | Okinawa | 28,853,116 | DRR129306 | https://figshare.com/s/c521469f649d420cb988 |
| Nesticidae | *Nesticus cooperi* | SRX652508 | - | - | 20,188,741 | SRR1514892 | - |
| Pimoidae | *Pimoa* sp. NG-2016 | SRX1560152 | - | - | 46,352,103 | SRR3144083 | - |

**Supplemental Table S9**

Summary statistics of *de novo* transcriptome assembly in Fig. 3

| **Family** | **Species** | **Scaffold number** | **Total scaffold length (bp)** | **Average scaffold length (bp)** | **Longest scaffold (bp)** | **N50 (bp) (# of scaffolds in N50)** | **N90 (bp) (# of scaffolds in N90)** |
| --- | --- | --- | --- | --- | --- | --- | --- |
| Araneidae | *Gasteracantha kuhli* | 67,029 | 55,013,603 | 820 | 15,650 | 1,545 (#10,278) | 292 (#43,749) |
| Araneidae | *Argiope aemula* | 76,016 | 61,246,979 | 805 | 10,537 | 1,492 (#11,904) | 294 (#50,086) |
| Araneidae | *Cyrtophora unicolor* | 65,863 | 36,832,143 | 559 | 11,718 | 810 (#11,017) | 236 (#48,804) |
| Araneidae | *Zygiella dispar* | 91,870 | 86,861,545 | 945 | 11,846 | 1,975 (#12,780) | 331 (#57,013) |
| Araneidae | *Araneus seminiger* | 77,232 | 54,169,854 | 701 | 12,279 | 1,237 (#11,473) | 261 (#53,102) |
| Araneidae | *Cyclosa octotuberculata* | 132,346 | 98,498,160 | 744 | 13,938 | 1,451 (#18,032) | 265 (#89,015) |
| Araneidae | *Gasteracantha hasselti* | 65,748 | 66,126,472 | 1,005 | 22,443 | 1,785 (#10,454) | 389 (#41,543) |
| Araneidae | *Macracantha arcuata* | 45,468 | 39,676,790 | 872 | 15,476 | 1,436 (#8,118) | 352 (#30,263) |
| Araneidae | *Micrathena gracilis* | 32,919 | 33,299,371 | 1011 | 30,147 | 1,646 (#5,503) | 415 (#21,704) |
| Araneidae | *Verrucosa arenata* | 61,239 | 44,944,211 | 733 | 26,669 | 1,226 (#9,046) | 281 (#41,996) |
| Araneidae | *Nephila clavipes* | 94,439 | 72,506,863 | 767 | 40,000 | 1,534 (#11,983) | 273 (#62,842) |
| Buthidae | *Centruroides vittatus* | 49,358 | 31,713,982 | 642 | 9,380 | 994 (#8,642) | 262 (#35,403) |
| Linyphiidae | *Frontinella communis* | 90,898 | 53,658,660 | 590 | 15,077 | 824 (#16,508) | 252 (#66,763) |
| Nesticidae | *Neoscona theisi* | 84,574 | 67,335,381 | 796 | 11,991 | 1,491 (#12,690) | 283 (#55,695) |
| Nesticidae | *Nesticus cooperi* | 29,177 | 25,877,469 | 886 | 14,414 | 1,359 (#5,382) | 378 (#19,937) |
| Pimoidae | *Pimoa* sp. NG-2016 | 72,445 | 43,313,757 | 597 | 23,267 | 827 (#12,530) | 254 (#53,090) |

**Supplemental Table S10**

BUSCO result of *de novo* transcriptome assembly in Fig. 3

| **Family** | **Species** | **Complete BUSCOs (%)** | **Complete and single-copy BUSCOs (%)** | **Complete and duplicated BUSCOs (%)** | **Fragmented BUSCOs (%)** | **Missing BUSCOs (%)** | **Total BUSCO groups searched** |
| --- | --- | --- | --- | --- | --- | --- | --- |
| Araneidae | *Gasteracantha kuhli* | 94.1 | 67.7 | 26.4 | 5.3 | 0.6 | 303 |
| Araneidae | *Argiope aemula* | 97.7 | 76.6 | 21.1 | 1.7 | 0.6 | 303 |
| Araneidae | *Cyrtophora unicolor* | 68.3 | 54.8 | 13.5 | 23.8 | 7.9 | 303 |
| Araneidae | *Zygiella dispar* | 97.4 | 61.4 | 36 | 2.6 | 0 | 303 |
| Araneidae | *Araneus seminiger* | 90.1 | 73.3 | 16.8 | 8.9 | 1 | 303 |
| Araneidae | *Cyclosa octotuberculata* | 98 | 66.3 | 31.7 | 0.7 | 1.3 | 303 |
| Araneidae | *Gasteracantha hasselti* | 96 | 70.3 | 25.7 | 3 | 1 | 303 |
| Araneidae | *Macracantha arcuata* | 93.1 | 77.6 | 15.5 | 5.6 | 1 | 303 |
| Araneidae | *Micrathena gracilis* | 82.5 | 72.6 | 9.9 | 9.6 | 8 | 303 |
| Araneidae | *Verrucosa arenata* | 79.5 | 69.6 | 9.9 | 15.8 | 5 | 303 |
| Araneidae | *Nephila clavipes* | 99.4 | 83.2 | 16.2 | 0.3 | 0 | 303 |
| Buthidae | *Centruroides vittatus* | 77.9 | 67.3 | 10.6 | 18.2 | 4 | 303 |
| Linyphiidae | *Frontinella communis* | 84.5 | 62.7 | 21.8 | 14.9 | 1 | 303 |
| Nesticidae | *Neoscona theisi* | 94.7 | 66 | 28.7 | 4.3 | 1 | 303 |
| Nesticidae | *Nesticus cooperi* | 80.2 | 66 | 14.2 | 13.9 | 6 | 303 |
| Pimoidae | *Pimoa* sp. NG-2016 | 73.6 | 62.7 | 10.9 | 22.1 | 4 | 303 |

**Supplemental Table S11**

MaSp3 conservation analysis in spiders

| **Family** | **Species** |  | **Accession number** | **MaSp3 BLASTP search results** | |
| --- | --- | --- | --- | --- | --- |
|  |  |  |  | **head** | **tail** |
| Agelenidae | *Agelenopsis emertoni* |  | SRR1514895 |  |  |
| Agelenidae | *Agelenopsis pennsylvanica* |  | SRR1329248 |  |  |
| Agelenidae | *Agelenopsis pennsylvanica* |  | SRR1329250 |  |  |
| Agelenidae | *Neoramia* sp. RK-2018 |  | SRR6998660 |  |  |
| Amaurobiidae | *Callobius* sp. NG-2016 |  | SRR3144088 |  |  |
| Amphinectidae | *Amphinecta* sp. RK-2018 |  | SRR6997628 |  |  |
| Amphinectidae | *Metaltella simoni* |  | SRR3144079 |  |  |
| Anapidae | *Novanapis* sp. RK-2018 |  | SRR6998663 | 5.00E-16 |  |
| Anapidae | *Spinanapis* sp. RK-2018 |  | SRR6998907 | 3.00E-22 |  |
| Antrodiaetidae | *Aliatypus coylei* |  | SRR1514876 | 4.00E-11 |  |
| Antrodiaetidae | *Antrodiaetus unicolor* |  | SRR1514897 |  |  |
| Araneidae | *Araneus marmoreus* |  | SRR6997754 |  |  |
| Araneidae | *Araneus seminiger* | *** | DRR129311 | 8.00E-73 | 1.00E-38 |
| Araneidae | *Argiope aemula* | *** | DRR129308 | 1.00E-40 | 7.00E-12 |
| Araneidae | *Arkys* sp. RK-2018 |  | SRR6997752 |  |  |
| Araneidae | *Cyclosa octotuberculata* | *** | DRR129312 | 4.00E-27 | 6.00E-08 |
| Araneidae | *Cyrtophora* sp. RK-2018 |  | SRR6997738 |  |  |
| Araneidae | *Cyrtophora unicolor* | *** | DRR129309 | 1.00E-39 | 5.00E-11 |
| Araneidae | *Demadiana* sp. RK-2018 |  | SRR6997740 | 3.00E-20 |  |
| Araneidae | *Gasteracantha hasselti* |  | SRR1048659 | 4.00E-31 | 4.00E-14 |
| Araneidae | *Gasteracantha kuhli* | *** | DRR129307 | 1.00E-27 | 2.00E-09 |
| Araneidae | *Macracantha arcuata* |  | SRR1048826 | 8.00E-31 | 7.00E-13 |
| Araneidae | *Micrathena gracilis* |  | SRR1514882 |  |  |
| Araneidae | *Neoscona arabesca* |  | SRR1145741 |  |  |
| Araneidae | *Nephila clavipes* |  | SRR5139327 |  |  |
| Araneidae | *Verrucosa arenata* |  | SRR3144087 |  |  |
| Araneidae | *Zygiella dispar* | *** | DRR129310 |  |  |
| Araneidae | *Neoscona theisi* | *** | DRR129306 | 3.00E-23 | 8.00E-14 |
| Archaeidae | *Austrarchaea* sp. RK-2018 |  | SRR6997750 |  |  |
| Atypidae | *Sphodros rufipes* |  | SRR1514908 |  |  |
| Austrochilidae | *Austrochilus forsteri* |  | SRR6997749 |  |  |
| Austrochilidae | *Hickmania troglodytes* |  | SRR6997862 |  |  |
| Caponiidae | *Calponia harrisonfordi* |  | SRR3144089 |  |  |
| Clubionidae | *Clubiona* sp. RK-2018 |  | SRR6997741 | 1.00E-19 |  |
| Corinnidae | *Falconina gracilis* |  | SRR6997866 |  |  |
| Corinnidae | *Nyssus* sp. RK-2018 |  | SRR6998658 |  |  |
| Ctenidae | *Anahita punctulata* |  | SRR3144072 |  |  |
| Ctenidae | *Cupiennius coccineus* |  | SRR7028538 |  |  |
| Ctenizidae | *Cyclocosmia truncata* |  | SRR1514884 |  |  |
| Ctenizidae | *Hebestatis theveneti* |  | SRR1514887 |  |  |
| Cyatholipidae | *Tekelloides* sp. 1 RK-2018 |  | SRR6998919 |  |  |
| Cyatholipidae | *Tekelloides* sp. 2 RK-2018 |  | SRR6998920 | 2.00E-16 |  |
| Cycloctenidae | *Cycloctenus* sp. RK-2018 |  | SRR6997743 |  |  |
| Deinopidae | *Deinopis longipes* |  | SRR1514879 |  |  |
| Deinopidae | *Deinopis* sp. RK-2018 |  | SRR6997737 |  |  |
| Deinopidae | *Menneus* sp. RK-2018 |  | SRR6997878 |  |  |
| Desidae | *Paramatachia* sp. RK-2018 |  | SRR6998654 |  |  |
| Dictynidae | *Cicurina travisae* |  | SRR1654705 |  |  |
| Dictynidae | *Cicurina vibora* |  | SRR1514883 |  |  |
| Diguetidae | *Diguetia* sp. NG-2016 |  | SRR3144093 |  |  |
| Dipluridae | *Microhexura montivaga* |  | SRR1514890 |  |  |
| Drymusidae | *Drymusa* sp. RK-2018 |  | SRR6997739 |  |  |
| Dysderidae | *Dysdera crocata* |  | SRR1328258 |  |  |
| Eresidae | *Stegodyphus mimosarum* |  | SRR1015313 |  |  |
| Euctenizidae | *Aptostichus stephencolberti* |  | SRR1514874 |  |  |
| Euctenizidae | *Promyrmekiaphila clathrata* |  | SRR1514896 |  |  |
| Filistatidae | *Filistata insidiatrix* |  | SRR6997865 |  |  |
| Filistatidae | *Kukulcania hibernalis* |  | SRR1514878 |  |  |
| Gnaphosidae | *Anzacia* sp. RK-2018 |  | SRR6997629 |  |  |
| Gnaphosidae | *Sergiolus capulatus* |  | SRR1514903 |  |  |
| Gradungulidae | *Gradungula sorenseni* |  | SRR6997863 |  |  |
| Gradungulidae | *Pianoa isolata* |  | SRR6998914 |  |  |
| Gradungulidae | *Progradungula otwayensis* |  | SRR6998916 |  |  |
| Gradungulidae | *Tarlina* sp. RK-2018 |  | SRR6998918 |  |  |
| Hahniidae | *Calymmaria persica* |  | SRR3144091 | 3.00E-14 |  |
| Hahniidae | *Calymmaria* sp. RK-2018 |  | SRR6997746 | 4.00E-12 |  |
| Hersiliidae | *Tamopsis* sp. RK-2018 |  | SRR6998917 |  |  |
| Hexathelidae | *Macrothele calpeiana* |  | SRR6994009 |  |  |
| Hexathelidae | *Porrhothele* sp. RK-2018 |  | SRR6997604 |  |  |
| Homalonychidae | *Homalonychus theologus* |  | SRR3144075 |  |  |
| Huttoniidae | *Huttonia palpimanoides* |  | SRR6997861 |  |  |
| Hypochilidae | *Hypochilus gertschi* |  | SRR6997860 |  |  |
| Hypochilidae | *Hypochilus pococki* |  | SRR1514889 |  |  |
| Idiopidae | *Idiops bersebaensis* |  | SRR1514907 |  |  |
| Lamponidae | *Lampona* sp. RK-2018 |  | SRR6997872 |  |  |
| Leptonetidae | *Archoleptoneta schusteri* |  | SRR6997753 |  |  |
| Leptonetidae | *Calileptoneta californica* |  | SRR3144085 |  |  |
| Linyphiidae | *Frontinella communis* |  | SRR1145739 | 6.00E-16 |  |
| Linyphiidae | *Laminacauda parvipalpis* |  | SRR6997873 |  |  |
| Linyphiidae | *Novafroneta gladiatrix* |  | SRR6998662 |  |  |
| Liocranidae | Unclassified Liocranidae |  | SRR6997875 |  |  |
| Liphistiidae | *Liphistius malayanus* |  | SRR1145736 |  |  |
| Liphistiidae | *Liphistius* sp. |  | SRR1514873 |  |  |
| Lycosidae | *Allocosa alticeps* |  | SRR6997626 |  |  |
| Lycosidae | *Schizocosa rovneri* |  | SRR1514894 |  |  |
| Malkaridae | *Perissopmeros* sp. RK-2018 |  | SRR6998657 |  |  |
| Malkaridae | Unclassified Malkaridae |  | SRR6997874 |  |  |
| Malkaridae | Unclassified Malkaridae |  | SRR6997869 | 3.00E-11 |  |
| Mecicobothriidae | *Megahexura fulva* |  | SRR1514891 |  |  |
| Mecysmaucheniidae | *Mecysmauchenius* sp. RK-2018 |  | SRR6997871 |  |  |
| Mimetidae | *Australomimetus* sp. RK-2018 |  | SRR6997751 | 9.00E-18 |  |
| Mimetidae | *Ero leonina* |  | SRR1514886 | 2.00E-20 |  |
| Miturgidae | *Teminius* sp. RK-2018 |  | SRR6998921 |  |  |
| Mysmenidae | *Microdipoena guttata* |  | SRR1333842 | 5.00E-16 |  |
| Mysmenidae | *Microdipoena jobi* |  | SRR6998666 | 3.00E-17 |  |
| Mysmenidae | *Mysmena leichhardti* |  | SRR6998665 | 1.00E-19 |  |
| Nemesiidae | *Calisoga longitarsis* |  | SRR1514875 |  |  |
| Nemesiidae | *Damarchus* sp. NG-2016 |  | SRR3144092 |  |  |
| Nemesiidae | *Pionothele* sp. NG-2014 |  | SRR1514906 |  |  |
| Nemesiidae | *Stanwellia* sp. RK-2018 |  | SRR6997603 | 2.00E-12 |  |
| Nephilidae | *Nephila clavipes* |  | SRR1514901 |  |  |
| Nesticidae | *Nesticus bishopi* |  | SRR1655191 | 9.00E-29 |  |
| Nesticidae | *Nesticus cooperi* |  | SRR1514892 | 7.00E-29 |  |
| Nicodamidae | *Megadictyna thilenii* |  | SRR6997870 |  |  |
| Nicodamidae | Unclassified Nicodamidae |  | SRR7028539 |  |  |
| Nicodamidae | Unclassified Nicodamidae |  | SRR6998661 |  |  |
| Ochyroceratidae | *Ochyrocera* sp. RK-2018 |  | SRR7028536 |  |  |
| Oecobiidae | *Oecobius cellariorum* |  | SRR1365089 |  |  |
| Oonopidae | *Ischnothyreus* sp. RK-2018 |  | SRR6997859 |  |  |
| Oonopidae | *Opopaea* sp. RK-2018 |  | SRR6998659 |  |  |
| Orsolobidae | *Maoriata* sp. RK-2018 |  | SRR6997868 |  |  |
| Orsolobidae | Unclassified Orsolobidae |  | SRR6998651 |  |  |
| Oxyopidae | *Peucetia longipalpis* |  | SRR1514898 |  |  |
| Palpimanidae | *Otiothops birabeni* |  | SRR6998652 |  |  |
| Palpimanidae | *Palpimanus gibbulus* |  | SRR6998653 |  |  |
| Pararchaeidae | *Pararchaea alba* |  | SRR6998655 | 1.00E-21 |  |
| Paratropididae | *Paratropis* sp. |  | SRR1514893 |  |  |
| Periegopidae | *Periegops suteri* |  | SRR6998656 |  |  |
| Pholcidae | *Pholcus phalangioides* |  | SRR3144082 |  |  |
| Pimoidae | *Pimoa* sp. 2 RK-2018 |  | SRR6998915 |  |  |
| Pimoidae | *Pimoa* sp. NG-2016 |  | SRR3144083 | 1.00E-18 |  |
| Pisauridae | *Dolomedes triton* |  | SRR3144094 |  |  |
| Prodidomidae | *Molycria* sp. RK-2018 |  | SRR6998664 | 1.00E-14 |  |
| Salticidae | *Habronattus signatus* |  | SRR1514888 | 3.00E-14 |  |
| Salticidae | *Habronattus ustulatus* |  | SRR1656783 | 4.00E-13 |  |
| Scytodidae | *Scytodes globula* |  | SRR6998911 |  |  |
| Scytodidae | *Scytodes thoracica* |  | SRR1514872 |  |  |
| Segestriidae | *Segestria* sp. NG-2016 |  | SRR3144084 |  |  |
| Selenopidae | *Karaops raveni* |  | SRR6997858 |  |  |
| Senoculidae | *Senoculus* sp. RK-2018 |  | SRR7028535 |  |  |
| Sicariidae | Loxosceles deserta |  | SRR3144077 |  |  |
| Sparassidae | *Caayguara ybytyriguara* |  | SRR6997747 | 4.00E-19 |  |
| Stiphidiidae | *Cambridgea* sp. RK-2018 |  | SRR6997745 |  |  |
| Synaphridae | *Cepheia longiseta* |  | SRR6997742 |  |  |
| Synotaxidae | *Meringa* sp. RK-2018 |  | SRR6997877 | 3.00E-17 |  |
| Synotaxidae | *Physoglenes* sp. RK-2018 |  | SRR6998913 | 7.00E-16 |  |
| Synotaxidae | *Runga* sp. RK-2018 |  | SRR6998910 | 3.00E-17 |  |
| Synotaxidae | *Synotaxus turbinatus* |  | SRR6998908 | 2.00E-22 |  |
| Tetragnathidae | *Leucauge venusta* |  | SRR1145740 | 2.00E-23 |  |
| Tetragnathidae | *Meta ovalis* |  | SRR6997876 |  |  |
| Tetragnathidae | *Nanometa* sp. RK-2018 |  | SRR6998667 |  |  |
| Tetragnathidae | *Tetragnatha tantalus* |  | SRR1427108 |  |  |
| Theraphosidae | *Acanthoscurria geniculata* |  | SRR1024076 |  |  |
| Theraphosidae | *Aphonopelma iviei* |  | SRR1514871 |  |  |
| Theraphosidae | *Trichopelma laselva* |  | SRR1514881 |  |  |
| Theridiidae | *Anelosimus eximius* |  | SRR6997627 | 5.00E-21 |  |
| Theridiidae | *Euryopis* sp. RK-2018 |  | SRR6997867 |  |  |
| Theridiidae | *Latrodectus tredecimguttatus* |  | SRR954929 |  |  |
| Theridiidae | *Theridion* sp. NG-2014 |  | SRR1514902 |  |  |
| Theridiosomatidae | *Baalzebub* sp. RK-2018 |  | SRR6997748 | 2.00E-23 |  |
| Theridiosomatidae | *Theridiosoma gemmosum* |  | SRR6998922 |  |  |
| Theridiosomatidae | *Theridiosoma savannum* |  | SRR7028533 | 6.00E-19 |  |
| Thomisidae | *Misumenoides formosipes* |  | SRR3144080 |  |  |
| Thomisidae | *Sidymella* sp. RK-2018 |  | SRR6998912 |  |  |
| Trachelidae | *Trachelas tranquillus* |  | SRR1329247 | 1.00E-17 |  |
| Trochanteriidae | *Rebilus* sp. RK-2018 |  | SRR6998909 |  |  |
| Uloboridae | *Philoponella herediae* |  | SRR1514880 | 1.00E-19 |  |
| Uloboridae | *Uloborus glomosus* |  | SRR1328334 | 9.00E-19 |  |
| Uloboridae | *Zosis* sp. RK-2018 |  | SRR6998924 |  |  |
| Zodariidae | *Cybaeodamus taim* |  | SRR6997744 |  |  |
| Zodariidae | *Forsterella* sp. RK-2018 |  | SRR6997864 |  |  |
| Zoropsidae | *Tengella radiata* |  | SRR7028532 |  |  |
| Zoropsidae | *Uliodon* sp. RK-2018 |  | SRR6998923 |  |  |
| * Sequenced by this study | |  |  | Blast E-value | Blast E-value |

**Supplemental Table S12**

Proteome data

| **ID** | **4152-C2-1** | **4153-C1-1** | **4160-C1-1** | **4162-C1-1** | **4162-C2-1** | **4169-C1-1** | **4169-C2-1** | **4180-C1-1** | **4181-C2-1** | **4184-C1-1** | **4184-C2-1** |
| --- | --- | --- | --- | --- | --- | --- | --- | --- | --- | --- | --- |
| g10361.t1 | 0.34 | 0.22 | 0.22 | 0.63 | 0.41 | 0.35 | 0.28 | 0.28 | 0.71 | 0.28 | 0.34 |
| g133719.t1 | 0.11 | 0.31 | 0.17 | 0.38 | 0.05 | 0.18 | 0.11 | 0.17 | 0.45 | 0.06 | 0.11 |
| g147648.t1 | 0.31 | 0.15 | 0.98 | 0.73 | 0.98 | 0.07 | 0.07 | 0.61 | 0.61 | 0.07 | 0.07 |
| g160600.t1 | 1.18 | 0.68 | 0.68 | 0.68 | 0.3 | 1.22 | 0.3 | 1.83 | 0.68 | 0.3 | 0.3 |
| g171745.t1 (MaSp2A) | 0.05 | 0.06 | 0.06 | 0.05 | 0.1 | 0.05 | 0.03 | 0.06 | 0.05 | 0.03 | 0.05 |
| g190809.t1 | 0.41 | 0.41 | 0.26 | 0.42 | 0.42 | 0.43 | 0.41 | 0.41 | 0.41 | 0.43 | 0.42 |
| g205672.t1 | 3.87 | 2.28 | 6.23 | 2.28 | 3.88 | 2.34 | 1.21 | 3.87 | 3.87 | 0.49 | 1.21 |
| g244936.t1 | 0.11 | 0.24 | 0.24 | 0.24 | 0.24 | 0.12 | 0.24 | 0.24 | 0.24 | 0.12 | 0.12 |
| g42745.t1 | 0.41 | 0.41 | 0.41 | 0.67 | 0.67 | 0.69 | 0.41 | 0.67 | 0.67 | 0.42 | 0.41 |
| g51538.t1 | 0.12 | 0.44 | 0.48 | 0.13 | 0.51 | 0.08 | 0.12 | 0.65 | 0.97 | 0.19 | 0.09 |
| g7867.t1 (MaSp3) | 0.2 | 0.24 | 0.66 | 0.43 | 0.45 | 0.25 | 0.24 | 0.24 | 0.63 | 0.25 | 0.23 |
| g80451.t1 | 0.98 | 0.98 | 2.42 | 1.28 | 0.98 | 0.32 | 0.31 | 0.98 | 1.6 | 0.32 | 0.32 |
| g163827.t1 | 0.12 | 0.06 | 0.06 | 0.26 | 0.19 | 0.06 | 0.06 | 0.12 | 0.19 | 0.06 | 0.06 |
| g246635.t1 (AcSp) | 0.19 | 0.05 | 0.03 | 0.03 | 0.03 | 0.07 | 0.01 | 0.07 | 0.13 | 0.01 | 0.02 |
| g60584.t1 | 0.2 | 0.2 | 0.2 | 0.2 | 0.2 | 0.2 | 0.2 | 0.2 | 0.2 | 0.2 | 0.2 |
| g13605.t1 | 0.34 | 0.34 | --- | 0.34 | 0.34 | 0.35 | 0.34 | 0.34 | 0.34 | 0.35 | 0.34 |
| g149801.t1 | 0.12 | 0.12 | 0.08 | 0.21 | --- | 0.08 | 0.08 | 0.17 | 0.26 | 0.08 | 0.04 |
| g228053.t1 | 0.18 | 0.53 | --- | 0.4 | 0.4 | 0.19 | 0.09 | 0.4 | 0.53 | 0.09 | 0.09 |
| g57794.t1 | 0.84 | 1.49 | 2.38 | 1.49 | --- | 0.86 | 0.36 | 1.49 | 1.49 | 1.53 | 0.84 |
| g190808.t1 | 1.21 | 0.49 | --- | 0.42 | 0.55 | 0.64 | 1.02 | 0.77 | 0.25 | 0.8 | 0.86 |
| g238778.t1 | 1.54 | 1.54 | 3.04 | 1.54 | 1.54 | 1.57 | 0.59 | 1.54 | 3.04 | --- | 1.55 |
| g248427.t1 | 0.1 | 1.07 | --- | 0.1 | 0.1 | 0.2 | 0.1 | 0.73 | 1.27 | 0.45 | 0.2 |
| g25303.t1 | 0.15 | 0.15 | 0.32 | --- | 0.15 | 0.15 | 0.15 | 0.15 | 0.74 | 0.15 | 0.15 |
| g121555.t1 (MaSp1) | 0.02 | 0.02 | 0.07 | 0.02 | --- | 0.02 | 0.02 | 0.02 | 0.02 | 0.02 | 0.02 |
| g163247.t1 | 0.86 | 0.15 | 0.07 | 0.15 | 0.74 | 0.24 | 0.15 | 0.23 | 0.99 | --- | 0.15 |
| g107621.t1 | 0.07 | 0.14 | --- | 0.23 | 0.23 | 0.15 | 0.14 | 0.14 | --- | 0.15 | 0.15 |
| g264011.t1 (MiSpA) | 0.16 | 0.13 | --- | 0.18 | 0.22 | 0.14 | 0.11 | 0.09 | --- | 0.1 | 0.1 |
| g52760.t1 | 0.06 | 0.06 | 0.26 | 0.33 | 0.06 | --- | 0.06 | 0.06 | 0.41 | --- | 0.06 |
| g6858.t1 | 0.26 | 0.12 | 0.12 | 0.42 | 0.26 | 0.13 | 0.12 | 0.12 | 0.26 | --- | --- |
| g22833.t1 | 0.28 | 0.65 | 0.65 | 0.28 | 0.65 | --- | 0.28 | 0.65 | 0.28 | --- | --- |
| g246443.t1 | 0.38 | --- | 1.12 | 0.71 | 1.64 | 0.39 | --- | 0.71 | 1.36 | 0.39 | --- |
| g197129.t1 | --- | 0.54 | 0.86 | --- | 1.54 | 0.07 | 0.06 | 0.54 | 1.69 | --- | 0.06 |
| g23090.t1 | 0.6 | 0.6 | 0.6 | 0.6 | 0.6 | --- | --- | 0.6 | 0.6 | --- | 0.61 |
| g40108.t1 | 0.22 | 1.22 | 0.49 | --- | 0.82 | --- | 0.49 | 0.82 | 1.22 | 0.23 | --- |
| g82874.t1 | --- | 0.44 | 0.44 | 0.44 | 0.44 | 0.45 | 0.44 | --- | --- | 0.45 | 0.44 |
| g48230.t1 | --- | 0.62 | 1.07 | --- | --- | 0.64 | 0.62 | 0.27 | 0.27 | 0.28 | 0.63 |
| g18254.t1 | 0.11 | 0.11 | --- | --- | --- | 0.11 | 0.11 | 0.11 | 0.11 | 0.11 | 0.11 |
| g149799.t1 | 0.7 | 0.42 | 0.7 | 0.7 | 0.42 | --- | 0.68 | --- | 0.42 | --- | --- |
| g16081.t1 (PySp) | 0.02 | 0.02 | 0.04 | 0.02 | 0.06 | --- | --- | 0.02 | 0.06 | --- | --- |
| g53882.t1 | --- | 0.53 | 1.35 | --- | 2.61 | --- | --- | 0.24 | 4.51 | 0.24 | 0.24 |
| g71039.t1 | 0.16 | 0.16 | --- | --- | 0.08 | 0.08 | 0.16 | 0.08 | --- | --- | 0.08 |
| g127164.t1 | --- | 0.15 | --- | --- | 0.24 | --- | 0.04 | 0.19 | 0.47 | 0.11 | 0.11 |
| g184723.t1 | --- | 0.19 | 0.19 | --- | 0.42 | --- | --- | 0.19 | 0.41 | 0.19 | 0.19 |
| g91085.t1 | --- | 0.17 | 0.17 | --- | 0.23 | --- | 0.05 | 0.29 | 0.36 | 0.11 | --- |
| g205695.t1 | 0.45 | 0.45 | --- | 0.45 | --- | 0.46 | 0.45 | --- | 0.45 | --- | 0.45 |
| g57564.t1 | --- | 0.07 | 0.07 | 0.07 | 0.07 | --- | 0.07 | --- | --- | 0.07 | 0.07 |
| g11399.t1 | --- | 1.51 | --- | 1.51 | --- | --- | --- | 1.51 | 1.51 | 0.59 | 0.59 |
| g108866.t1 | --- | 0.17 | --- | --- | --- | 0.18 | --- | 0.17 | 0.6 | 0.18 | 0.17 |
| g130607.t1 | --- | 1.05 | 1.31 | --- | 1.31 | --- | --- | 0.27 | 2.31 | 0.13 | --- |
| g230993.t1 | --- | 0.45 | --- | 0.45 | 0.45 | 0.46 | 0.45 | 0.45 | --- | --- | --- |
| g232218.t1 | --- | --- | 0.06 | 0.06 | --- | 0.06 | 0.06 | 0.06 | 0.06 | --- | --- |
| g113762.t1 | --- | 0.79 | 0.34 | --- | 0.34 | --- | --- | 0.62 | 1.39 | 0.49 | --- |
| g163633.t1 | 0.32 | --- | 1.01 | 0.52 | 0.75 | --- | --- | 0.32 | 1.31 | --- | --- |
| g168068.t1 | 0.23 | --- | 0.23 | 0.23 | 0.23 | --- | --- | 0.23 | 0.23 | --- | --- |
| g257199.t1 | --- | --- | --- | 0.14 | --- | 0.14 | 0.14 | --- | 0.14 | 0.14 | 0.14 |
| g42351.t1 | 0.33 | 0.07 | --- | 0.33 | --- | 0.08 | --- | 0.15 | 0.53 | --- | --- |
| g84419.t1 | --- | 1 | --- | --- | --- | 0.42 | --- | 0.42 | 1 | 0.42 | 0.42 |
| g45546.t1 | --- | --- | 1 | 0.42 | 1.01 | --- | --- | 0.42 | 0.42 | --- | --- |
| g10406.t1 | 0.23 | 0.23 | 0.23 | 0.23 | 0.23 | --- | --- | --- | --- | --- | --- |
| g184938.t1 | --- | 0.02 | --- | 0.04 | --- | 0.02 | 0.06 | 0.02 | --- | --- | --- |
| g230513.t1 | --- | 0.15 | --- | 0.15 | --- | 0.28 | 0.21 | 0.15 | --- | --- | --- |
| g60230.t1 | --- | 0.06 | --- | 0.06 | 0.06 | 0.06 | 0.13 | --- | --- | --- | --- |
| g70450.t1 | --- | 0.09 | 0.09 | 0.09 | --- | --- | --- | 0.09 | 0.09 | --- | --- |
| g127161.t1 | --- | 0.87 | 0.65 | --- | 0.65 | 0.47 | --- | 1.12 | --- | --- | --- |
| g228801.t1 | --- | 0.33 | --- | 0.33 | --- | --- | --- | 0.33 | 0.33 | 0.33 | --- |
| g619.t1 | 0.14 | 0.14 | 0.68 | 0.48 | --- | --- | --- | --- | --- | 0.14 | --- |
| g7111.t1 | 0.14 | 0.14 | --- | --- | 0.14 | --- | 0.14 | --- | 0.14 | --- | --- |
| g99511.t1 | 0.65 | --- | 0.65 | 0.65 | 0.65 | --- | --- | --- | 0.65 | --- | --- |
| g100864.t1 | 0.18 | --- | 0.18 | --- | --- | --- | 0.18 | 0.39 | 1.69 | --- | --- |
| g167690.t1 | --- | 0.04 | --- | 0.04 | --- | 0.04 | 0.04 | --- | --- | --- | 0.04 |
| g176359.t1 | --- | 0.31 | --- | 0.31 | --- | 1.27 | 0.31 | --- | 0.71 | --- | --- |
| g20321.t1 | 0.22 | --- | 0.35 | 0.82 | 0.65 | --- | --- | --- | 0.82 | --- | --- |
| g217492.t1 | --- | 0.38 | --- | --- | 0.36 | 0.39 | 0.38 | 0.38 | --- | --- | --- |
| g228458.t1 | 0.26 | --- | 0.26 | 0.26 | --- | 0.64 | --- | --- | 0.26 | --- | --- |
| g250740.t1 | 0.03 | 0.03 | --- | 0.03 | --- | --- | 0.03 | --- | --- | --- | 0.03 |
| g117149.t1 | --- | --- | --- | 0.13 | --- | --- | --- | 0.13 | --- | 0.13 | 0.13 |
| g157549.t1 | --- | --- | --- | 1.36 | 0.24 | --- | --- | 0.9 | --- | 0.55 | --- |
| g65976.t1 | 0.1 | 0.15 | --- | 0.15 | --- | --- | --- | 0.15 | --- | --- | --- |
| g107709.t1 | 0.98 | --- | 0.41 | --- | --- | --- | --- | 0.41 | --- | --- | 0.41 |
| g125042.t1 | 0.35 | 0.35 | --- | 0.35 | --- | --- | --- | --- | --- | --- | 0.35 |
| g154161.t1 | --- | --- | 0.04 | 0.04 | 0.04 | --- | --- | --- | 0.04 | --- | --- |
| g2315.t1 | --- | 0.33 | --- | 0.33 | --- | 0.33 | --- | 0.33 | --- | --- | --- |
| g48234.t1 | 0.06 | --- | 0.06 | 0.06 | --- | --- | --- | --- | 0.06 | --- | --- |
| g49484.t1 | --- | --- | --- | --- | 0.45 | --- | --- | 0.45 | 1.1 | 0.46 | --- |
| g109722.t1 | 0.07 | --- | --- | 0.15 | --- | --- | --- | --- | 0.07 | 0.15 | --- |
| g114860.t1 | --- | --- | 1.91 | 2.61 | 2.61 | --- | --- | 0.9 | --- | --- | --- |
| g145795.t1 | --- | --- | --- | --- | --- | --- | --- | 0.51 | 0.67 | 0.52 | 0.23 |
| g171746.t1 (MaSp2B) | --- | --- | 0.06 | 0.06 | 0.06 | --- | --- | --- | 0.06 | --- | --- |
| g205670.t1 | --- | 0.35 | --- | --- | 0.35 | --- | --- | 0.35 | 0.35 | --- | --- |
| g31787.t1 | --- | 1.21 | --- | 1.21 | 0.49 | --- | --- | 0.49 | --- | --- | --- |
| g59063.t1 | --- | --- | 0.27 | 0.27 | 0.13 | --- | --- | --- | 0.27 | --- | --- |
| g61017.t1 | --- | --- | --- | 0.26 | 0.26 | --- | --- | 0.26 | 0.26 | --- | --- |
| g632.t1 | 0.14 | 0.14 | --- | --- | 0.14 | --- | --- | --- | 0.14 | --- | --- |
| g6935.t1 | --- | 0.28 | 0.27 | --- | 1.04 | --- | --- | --- | 0.28 | --- | --- |
| g84068.t1 | --- | 0.22 | 0.22 | --- | --- | --- | --- | 0.1 | 0.35 | --- | --- |
| g142974.t1 | --- | --- | 0.36 | 0.36 | 0.36 | --- | --- | --- | 0.36 | --- | --- |
| g212182.t1 | --- | 0.18 | --- | --- | --- | 0.25 | 0.38 | --- | --- | --- | 0.31 |
| g228574.t1 | 0.32 | 0.32 | 0.32 | 0.32 | --- | --- | --- | --- | --- | --- | --- |
| g91084.t1 | 0.1 | --- | 0.34 | --- | 0.47 | --- | --- | --- | 0.47 | --- | --- |

There are no significant differences (t-test, *p* = 0.636)

**Supplemental Table S13**

Mechanical property

| **Spider sample ID** | **Nutrition status** | **Diameter (mm)** | **±** | **Ultimate strength (MPa)** | **±** | **Strain at break (%)** | **±** | **Toughness (MJ/m^3^)** | **±** |
| --- | --- | --- | --- | --- | --- | --- | --- | --- | --- |
| 4153 | Feeding control 1 | 4.47 | 0.58 | 930.74 | 314.43 | 26.22 | 10.17 | 82.29 | 62.45 |
| 4155 | Feeding control 1 | 5.28 | 0.76 | 993.25 | 266.79 | 23.40 | 7.13 | 125.24 | 47.07 |
| 4160 | Feeding control 1 | 4.27 | 0.39 | 835.07 | 200.45 | 14.61 | 3.03 | 50.09 | 18.27 |
| 4162 | Feeding control 1 | 4.96 | 1.36 | 948.19 | 291.63 | 24.05 | 6.98 | 89.24 | 75.23 |
| 4169 | Feeding control 1 | 6.11 | 1.85 | 764.87 | 298.59 | 18.26 | 6.96 | 58.28 | 46.52 |
| 4170 | Feeding control 1 | 4.73 | 0.90 | 773.59 | 125.64 | 20.67 | 14.81 | 77.01 | 50.67 |
| 4171 | Feeding control 1 | 4.35 | 0.45 | 1027.25 | 277.61 | 17.31 | 4.97 | 81.58 | 35.66 |
| 4180 | Feeding control 1 | 3.56 | 0.43 | 907.17 | 236.65 | 18.10 | 9.16 | 95.06 | 49.81 |
| 4181 | Feeding control 1 | 4.39 | 0.71 | 979.30 | 243.72 | 22.35 | 3.65 | 108.60 | 46.37 |
| 4184 | Feeding control 1 | 5.20 | 0.69 | 1054.73 | 336.41 | 19.33 | 7.47 | 107.15 | 49.97 |
| 4152 | Feeding control 2 | 3.95 | 0.51 | 1152.38 | 344.54 | 22.63 | 6.96 | 140.29 | 67.72 |
| 4153 | Feeding control 2 | 4.22 | 0.31 | 1034.55 | 230.86 | 26.29 | 8.33 | 119.85 | 51.30 |
| 4155 | Feeding control 2 | 4.54 | 1.22 | 987.04 | 207.11 | 20.73 | 9.49 | 104.12 | 29.94 |
| 4160 | Feeding control 2 | 4.36 | 0.23 | 797.14 | 189.40 | 14.66 | 4.78 | 56.79 | 27.84 |
| 4162 | Feeding control 2 | 4.08 | 0.53 | 908.30 | 401.04 | 18.81 | 4.12 | 80.91 | 103.63 |
| 4169 | Feeding control 2 | 6.60 | 2.35 | 871.69 | 269.06 | 25.22 | 6.04 | 84.92 | 50.38 |
| 4170 | Feeding control 2 | 4.54 | 0.55 | 878.79 | 196.53 | 21.09 | 6.16 | 67.55 | 39.60 |
| 4171 | Feeding control 2 | 3.39 | 0.41 | 680.08 | 215.89 | 8.80 | 4.92 | 31.83 | 32.42 |
| 4180 | Feeding control 2 | 4.06 | 1.02 | 900.96 | 187.68 | 16.96 | 5.08 | 70.17 | 35.74 |
| 4181 | Feeding control 2 | 3.48 | 0.24 | 948.25 | 220.55 | 16.94 | 10.35 | 85.11 | 77.23 |
| 4184 | Feeding control 2 | 4.66 | 1.82 | 1008.89 | 253.73 | 30.30 | 9.35 | 179.76 | 73.85 |
| 4152 | Starvation one week | 3.31 | 0.38 | 967.68 | 278.05 | 14.03 | 4.85 | 55.53 | 29.78 |
| 4153 | Starvation one week | 4.30 | 0.44 | 928.75 | 198.35 | 15.62 | 4.46 | 62.82 | 27.96 |
| 4155 | Starvation one week | 4.25 | 0.18 | 878.47 | 248.97 | 21.35 | 5.12 | 91.28 | 32.28 |
| 4160 | Starvation one week | 4.10 | 0.64 | 675.39 | 280.71 | 10.41 | 5.42 | 28.13 | 37.03 |
| 4162 | Starvation one week | 3.11 | 0.49 | 868.95 | 238.11 | 13.46 | 3.95 | 47.03 | 26.96 |
| 4169 | Starvation one week | 5.29 | 1.46 | 870.32 | 703.39 | 20.31 | 1.98 | 83.09 | 58.68 |
| 4170 | Starvation one week | 5.36 | 0.64 | 668.44 | 205.54 | 27.20 | 14.08 | 81.26 | 63.31 |
| 4171 | Starvation one week | 3.65 | 0.71 | 972.01 | 332.32 | 11.34 | 2.06 | 47.50 | 18.22 |
| 4180 | Starvation one week | 3.71 | 0.82 | 954.93 | 250.16 | 16.55 | 5.87 | 71.75 | 26.93 |
| 4181 | Starvation one week | 3.32 | 1.18 | 905.87 | 371.68 | 20.12 | 5.19 | 76.69 | 65.21 |
| 4184 | Starvation one week | 3.52 | 0.39 | 847.86 | 178.13 | 16.29 | 6.01 | 63.84 | 25.32 |

**References**

1 Garrison, N. L. *et al.* Spider phylogenomics: untangling the Spider Tree of Life. *PeerJ* **4**, e1719 (2016).
